# Supplementary material for: Dioxygen compatible electron donor-acceptor catalytic system and its enabled aerobic oxygenation
Source: Nat Commun. 2024 Feb 29;15:1886. doi: 10.1038/s41467-024-45866-z (PMC10904740; doi:10.1038/s41467-024-45866-z)
Supplement: Supplementary file 1 — Supplementary Information [file 41467_2024_45866_MOESM1_ESM.pdf]

Supplementary Information

Dioxygen Compatible Electron Donor-Acceptor Catalytic System and its Enabled Aerobic Oxygenation

Jialiang Wei[1,2], Junhong Meng[1], Caifang Zhang[1], Yameng Liu[1], Ning Jiao[1,2,3*]

[1] State Key Laboratory of Natural and Biomimetic Drugs, School of Pharmaceutical Sciences, Peking University, Xue Yuan Rd. 38, Beijing 100191

[2] Changping Laboratory, Yard 28, Science Park Road, Changping District, Beijing 102206, China.

[3] State Key Laboratory of Organometallic Chemistry Sciences, Chinese Academy of Sciences, Shanghai 200032

*Corresponding Author(s): jiaoning@pku.edu.cn.

Table of Contents

(A) General Remarks...S3

(B) Optimization of the Reaction Conditions...S6

(C) Representative Crude NMR Spectra of Reaction mixture..................S10

(D) Experimental Procedure and Analytical Data of Products...............S16

(E) Mechanistic Studies……………………...…………………….……..S30

(F) ^{1}H NMR, ^{13}C NMR Spectra of Products.…………………..…...…...…...S51

(G) Author Contributions…………………………………………………….S106

(H) References……………………………………………………………..…..S107

(A) General Remarks

All commercially available compounds were purchased from Sigma-Aldrich, Alfa-Aesar, Acros, Energy Chemical, Adamas, Bidepharmatech and Beijing Chemical Works, Ltd. Unless otherwise noted, materials obtained from commercial suppliers were used without further purification. Analysis of crude reaction mixture was done on an Agilent 7890 GC System with an Agilent 5975 Mass Selective Detector. Products were purified by flash chromatography on silica gel. ^{1}H-NMR spectra were recorded on Bruker AVANCE III-400 spectrometers. Chemical shifts (in ppm) were referenced TMS (0.00 ppm) or $CHCl_3$ (7.26 ppm) in $CDCl_3$. ^{13}C-NMR spectra were obtained by using the same NMR spectrometers and were calibrated with $CDCl_3$ (δ = 77.00 ppm). Mass spectra were recorded using a PE SCLEX QSTAR spectrometer. High resolution mass spectra were obtained with a Waters Xevo G2 Q-TOF mass spectrometer. Fluorescence spectra were obtained with a Cary Eclipse Agilent Technologies fluorescence spectrophotometer. UV-Vis absorption spectra were recorded using a Cary Eclipse Agilent Technologies Absorption spectrometer. Cyclic voltammetry (CV) experiments were performed on a CHI-660E electrochemical workstation by a three-electrode system, where glassy carbon electrode (GC, 0.07 cm^2) or GC disk as the work electrodes, platinum wire as counter electrode and 0.1 M $AgNO_3$/Ag electrode as reference electrode. All measured potentials in organic phase were corrected by ferrocene (*vs.* $Fc^{+/0}$), and potentials in aqueous solution were corrected by normal hydrogen electrode (*vs*. NHE). *n*-Bu_4NPF_6 were used as supporting electrolyte.

Photochemistry Reaction devices (Light source in detail)：

The light source used for photochemical experiments were blue LED light source with a power consumption of 72 watts (Supplementary Figure 1). For a single reaction, we used FaCai Blue LED (450 nm) light source (Supplementary Figure 1, left). For multi reactions, we used GeAo Chem Blue LED (450 nm) light source (Supplementary Figure 1, right).

It is noteworthy that the two light sources emit blue light at a wavelength of 450nm and have been measured to produce an optical power density of approximately 30 milliwatts per square centimeter. Furthermore, the reaction demonstrates a high level of consistency and repeatability.

The borosilicate glass reaction Schlenk tubes are purchased form Synthware Glass Co. LTD and no filters were applied. The distance from the light source to the irradiation vessel is 5-10 cm.

During the reaction process, due to the temperature control by the fan of the photoreactor, no significant temperature rise was observed in the entire reaction system, and the overall reaction temperature was maintained at a level close to room temperature (26 to 30 degrees Celsius).

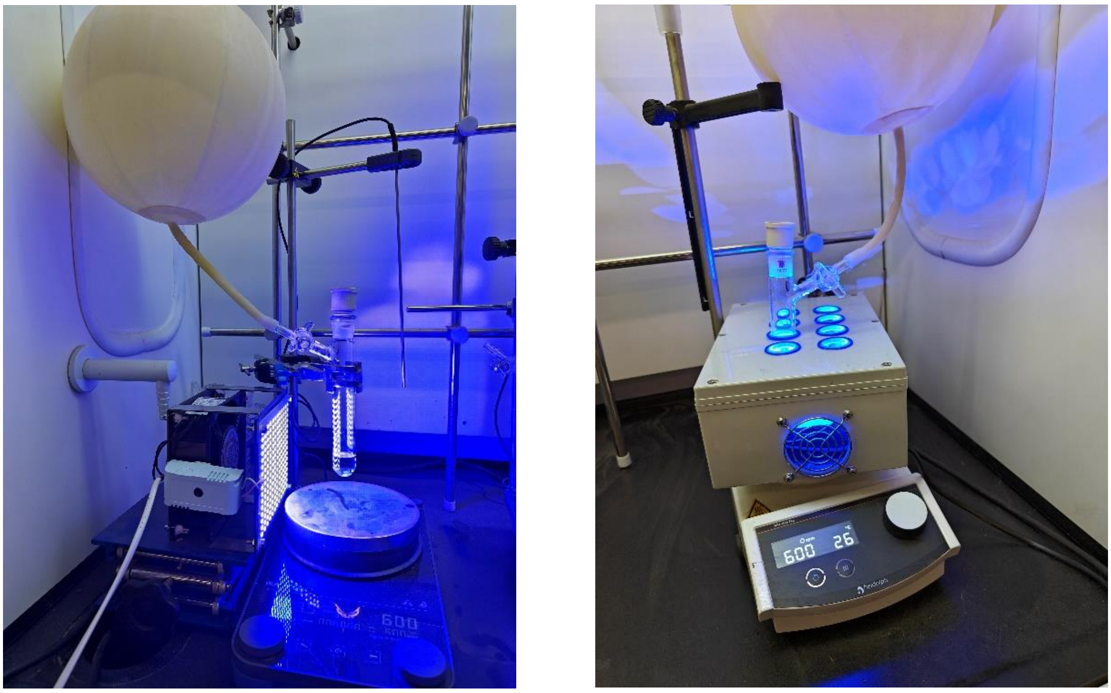

Supplementary Figure 1. Photochemistry Reaction devices for single reaction (left) and multi reactions (right)

(B) Optimization of the Reaction Conditions

Optimization of Donors

Reaction Conditions: (4-(tert-butyl) phenyl) boronic acid **1a** (0.5 mmol), different donor (0.5 mmol, 1.0 eq), 1,2-DNB acceptor (0.025 mmol, 5 mol%) in solvent anhydrous MeCN (4.0 mL) under oxygen (1 atm). Yields were determined by weighing of isolated product.

Supplementary Table 1. Optimization of Electron Donor[a]

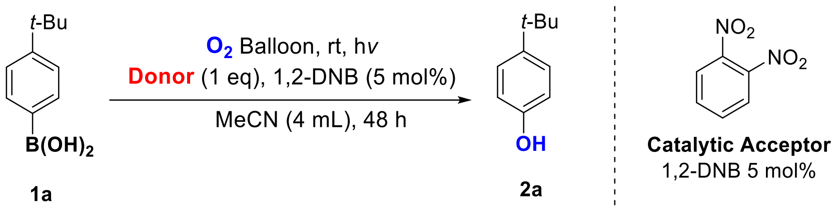

entry	Donor	Isolated Yield
**1**	**Tri-*n*-Butylamine**	**99%**
2	Tri-*n*-octylamine	29%
3	DABCO	Trace
4	Et_3N	28%
5	DIPEA	Trace
6	NPh_3	Trace
7	$PhNH_2$[b]	Trace
8	Pyrrolidine[b]	ND
9	Piperidine[b]	ND
10	Pyridine	ND
11	DMF	ND
12	PPh_3	ND
13	BINAP	ND
14	$P(OEt)_3$	ND
15	Tricyclohexyl phosphine	ND
16	Diphenyl sulfide	ND
17	Thioanisole	ND
18	Maleimide	ND

19	Succinimide	ND
20	*N,N*-dibutylbutyramide	ND
21	*N,N*-diethylacetamide	ND
22	*t*-BuOK	Complex Minxtre

[a] Reaction conditions: **1a** (0.5 mmol), donor (0.5 mmol), 1,2-DNB acceptor (0.025 mmol, 5 mol%) in solvent (4.0 mL) under oxygen (1 atm). [b] S_NAr side reaction with 1,2-DNB acceptor.

Optimization of Acceptors

Reaction Conditions: (4-(tert-butyl) phenyl) boronic acid **1a** (0.5 mmol), Tri-n-Butylamine (0.5 mmol, 1.0 eq), different acceptor (0.025 mmol, 5 mol%) in solvent anhydrous MeCN (4.0 mL) under oxygen (1 atm). Yields were determined by weighing of isolated product.

Supplementary Table 2. Optimization of Electron Acceptor[a]

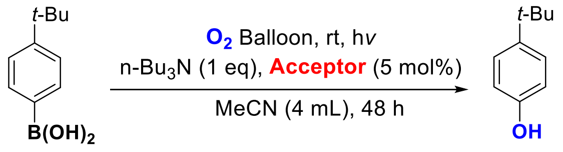

1a **2a**

entry	Acceptor	Isolated Yield
**1**	**1,2-DNB**	**99%**
2	1,3-DNB	34%
3	1,4-DNB	17%
4	Nitrobenzene	Trace
5	Benzenesulfonamide	Trace
6	Benzophenone	Trace
7	Methyl benzoate	ND
8	Benzoic acid	ND
9	Benzonitrile	ND

10	hexafluorobenzene	ND
11	PhF	ND
12	PhCl	ND
13	PhBr	ND
14	PhI	ND
15	Nitromethane	ND
16	Nitroethane	ND

[a] Reaction conditions: **1a** (0.5 mmol), Tri-*n*-Butylamine donor (0.5 mmol), acceptor (0.025 mmol, 5 mol%) in solvent (4.0 mL) under oxygen (1 atm).

Optimization of the amount of Electron Acceptors

Reaction Conditions: (4-(tert-butyl) phenyl) boronic acid **1a** (0.5 mmol), Tri-*n*-Butylamine (0.5 mmol, 1.0 eq), 1,2-DNB acceptor (different amounts) in solvent anhydrous MeCN (4.0 mL) under oxygen (1 atm). Yields were determined by weighing of isolated product.

Supplementary Table 3. Optimization of the amount of Electron Acceptor[a]

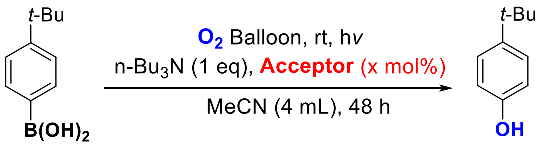

1a **2a**

entry	Amount of Acceptor 1,2-DNB	Isolated Yield
1	1 mol%	37%
2	1 mol%[b]	49%
3	2.5 mol%	46%
4	2.5 mol%[b]	85%
**5**	**5 mol%**	**99%**
6	10 mol%	99%

7	20 mol%	98%
8	50 mol%	96%
9	100 mol%	95%

[a] Reaction conditions: **1a** (0.5 mmol), Tri-*n*-Butylamine donor (0.5 mmol), acceptor (x mol%) in solvent (4.0 mL) under oxygen (1 atm). [b] The reaction time has been extended to 96 hours.

The 1,2-dinitrobenzene, serving as an acceptor, remained stable in this oxidation system and could continuously catalyze the reaction at a certain concentration. Therefore, despite the reduced reaction rate and subsequent decrease in yield resulting from the reduction in acceptor amount, extending the reaction time could further enhance the yield. However, due to the relatively mild nature of this oxygen activation system and the moderate rate of superoxide radical generation, considering the time cost, we ultimately opted for a catalyst dosage of 5% and a reaction time of 48 hours.

(C) Representative Crude NMR Spectra of Reaction mixture

Tips:

Due to the low molecular weight and low boiling points of some products, which make them volatile, accurate yields could not be obtained through conventional purification methods (column chromatography and then the use of a rotary evaporator under reduced pressure).

Therefore, for the specific product mentioned above, we instead reacted directly in a deuterated acetonitrile solution and added an internal standard (1,1,2,2-tetrachloroethane, TCE) after the reaction was complete for quantitative yield calculation.

For crude product with internal standard tetrachloroethane:

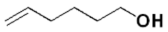

2aa

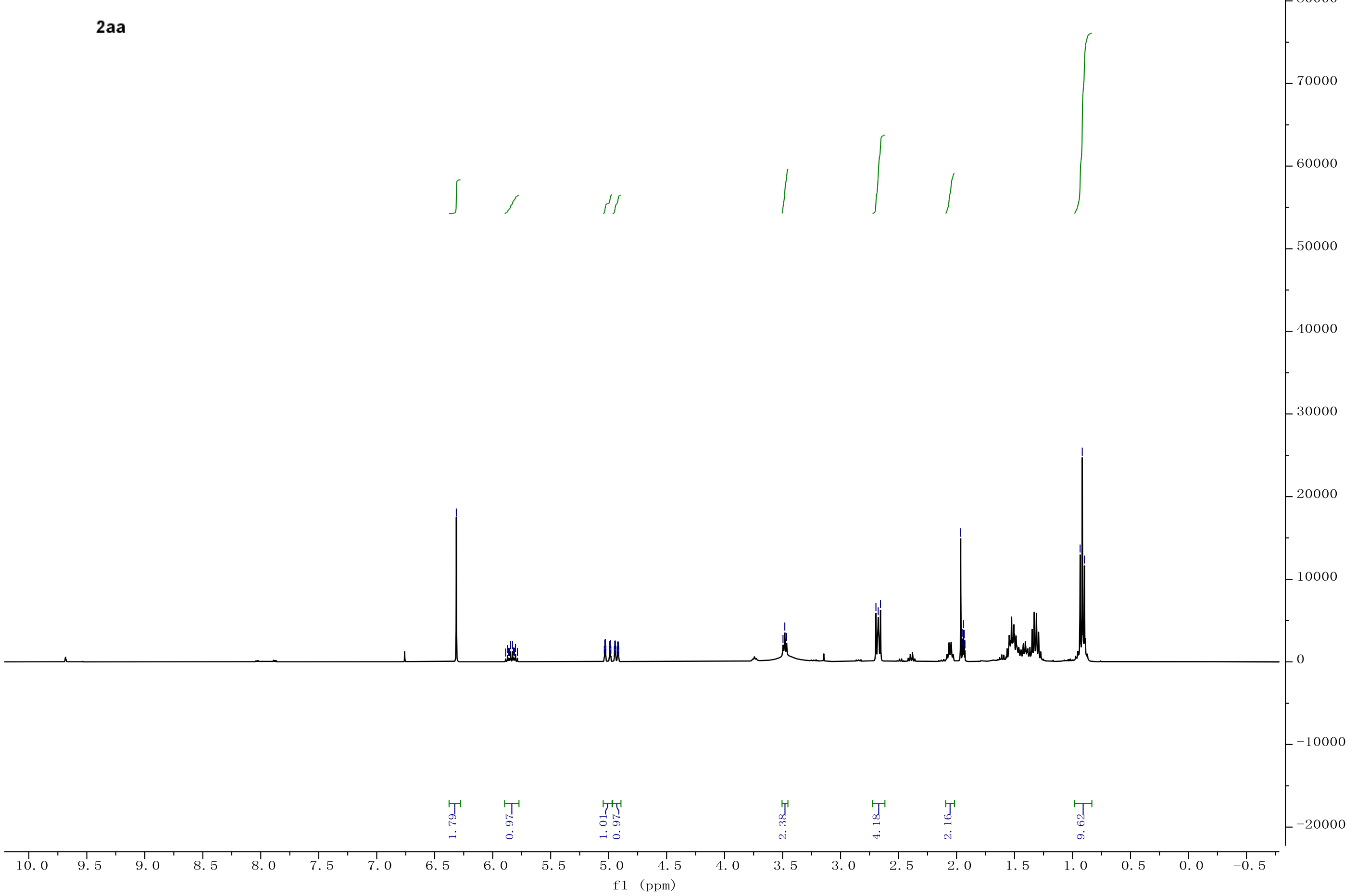

Supplementary Figure 2. ^{1}H NMR Spectrum for 2aa

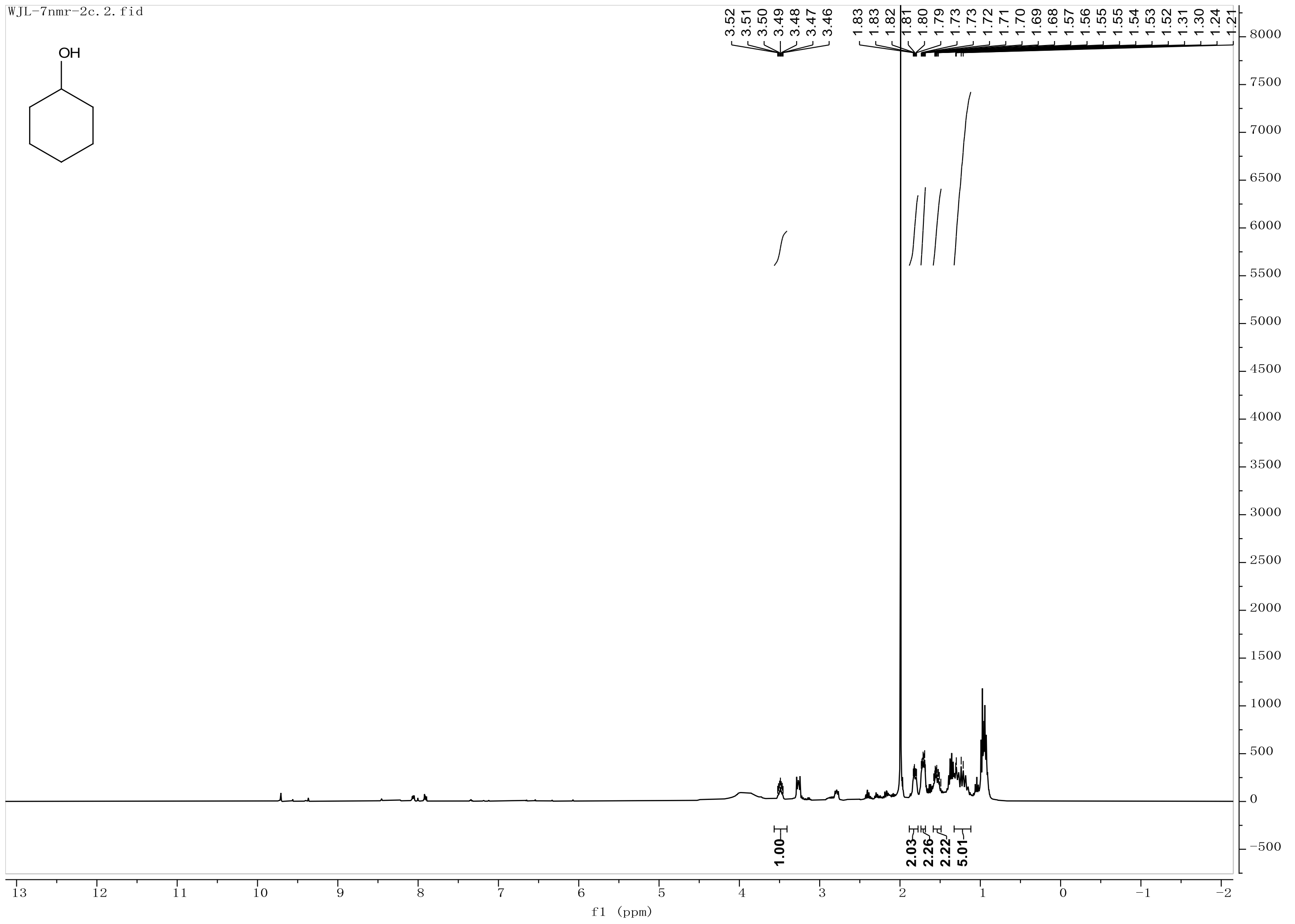

Supplementary Figure 3. ^{1}H NMR Spectrum for 2ab

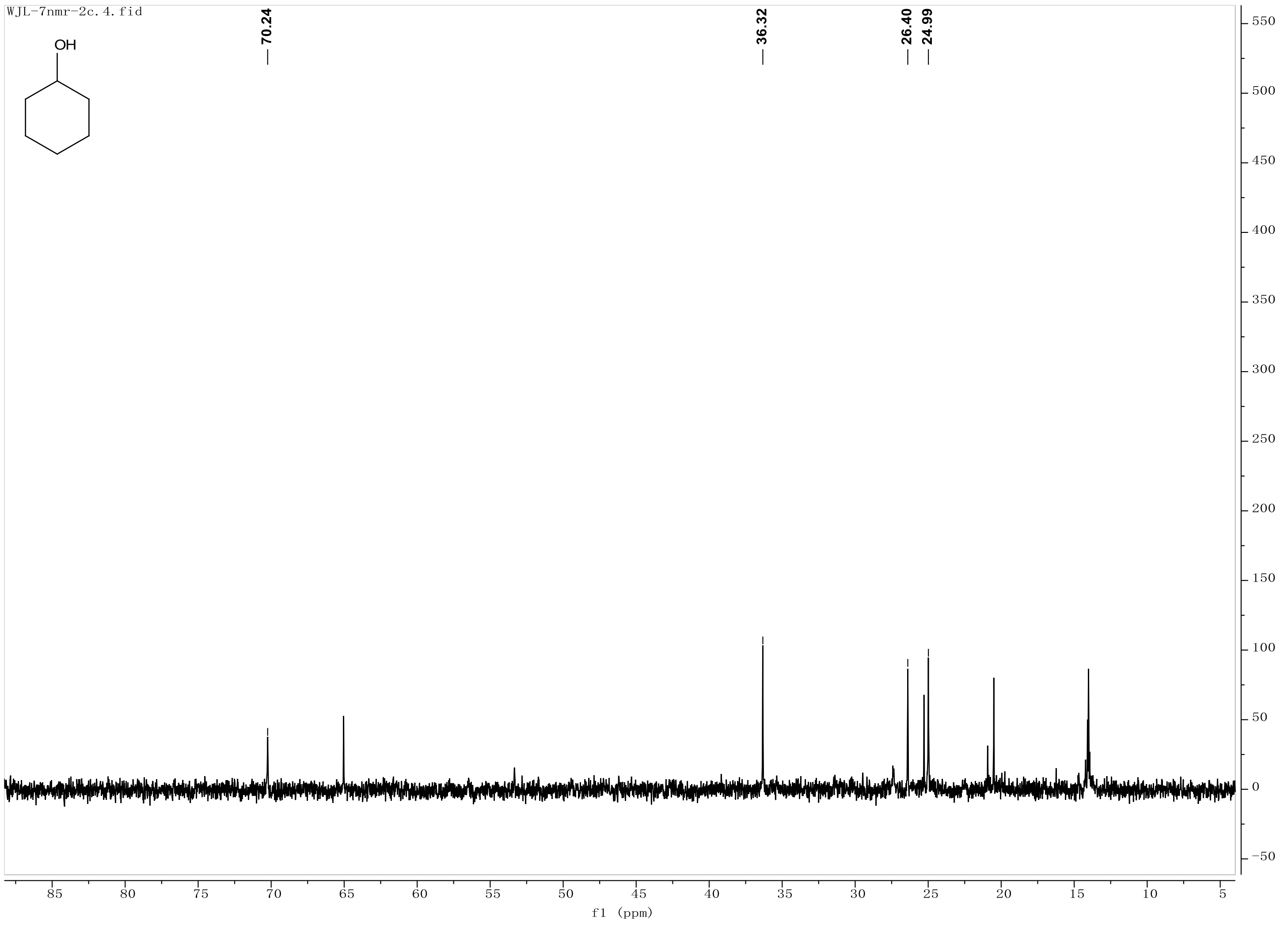

Supplementary Figure 4. ^{13}C NMR Spectrum for 2ab

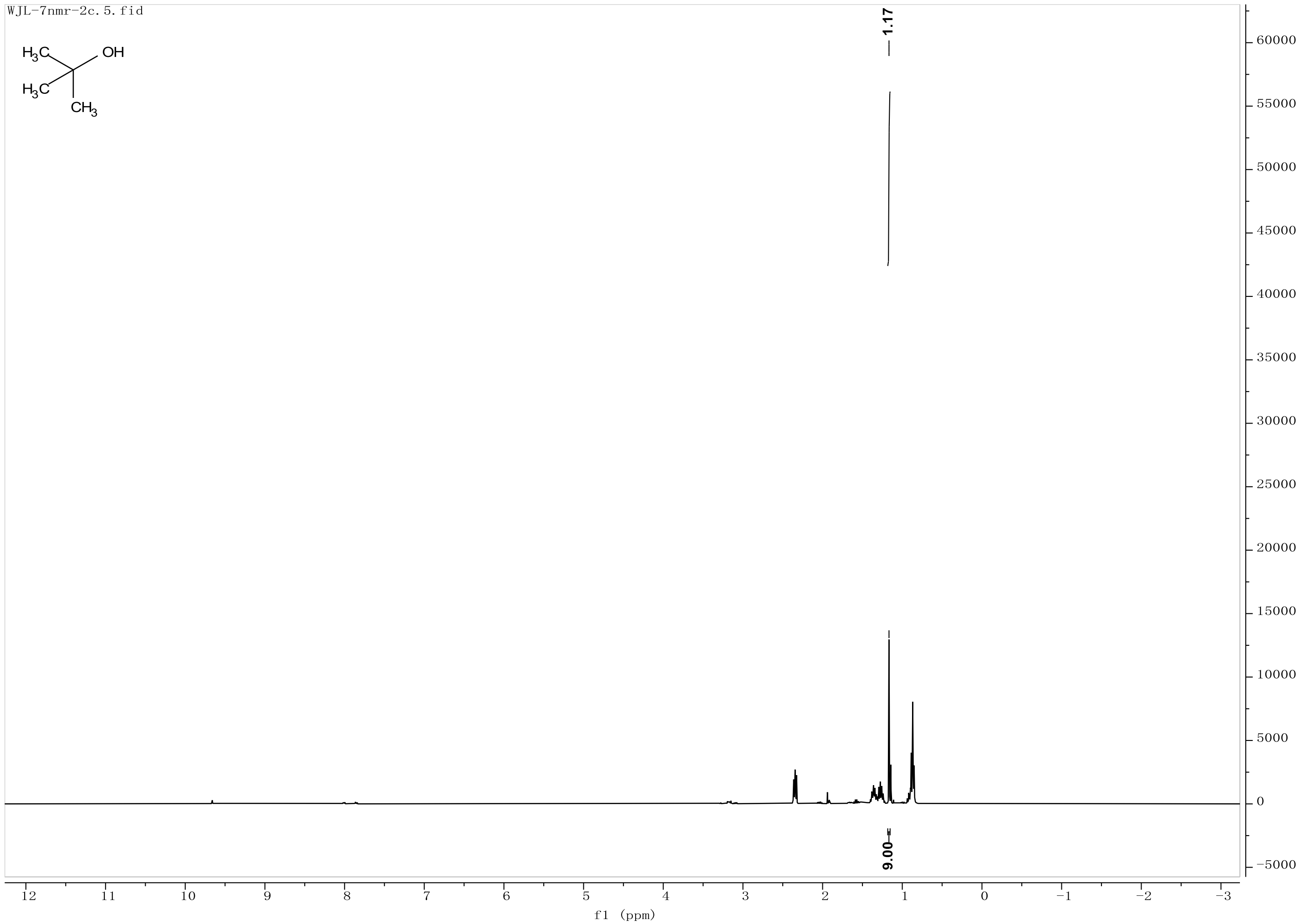

Supplementary Figure 5. ^{1}H NMR Spectrum for 2ac

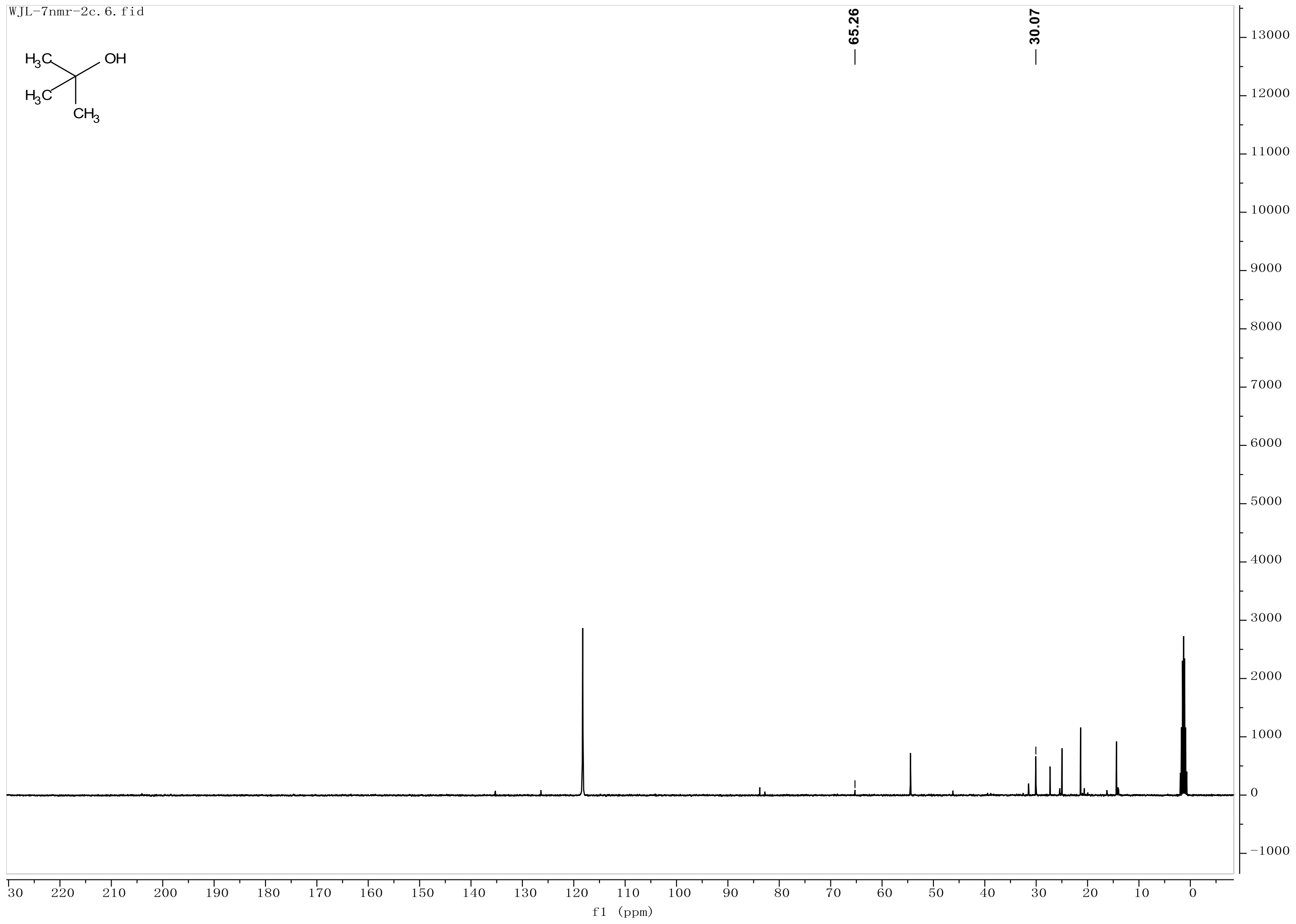

Supplementary Figure 6. ^{13}C NMR Spectrum for 2ac

(D) Experimental Procedure and Analytical Data of Products

General procedure for aerobic oxygenation of boric acid: To a dry 25 mL Schlenk tube, 0.5 mmol of boronic acid (if solid) and 4.3 mg (5 mol%) of 1,2-dinitrobenzene (1,2-DNB) were added. The tube was evacuated under reduced pressure and refilled with oxygen (3 times). Next, 120 μL of tri-*n*-butylamine (0.5 mmol, 1.0 eq) was added as a donor to the Schlenk tube. Anhydrous MeCN (4.0 mL) was then added using a syringe. The mixture was stirred at room temperature for 48 hours under irradiation with blue LED (450 nm). After completion of the reaction, the solvent was removed under vacuum and the crude products were purified by column chromatography.

Note: Some phenolic products are unstable under air conditions and are easily oxidized by oxygen in the air, causing the color of the product to darken. Therefore, aftertreatment should be done as soon as possible.

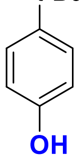

4-(tert-butyl)phenol (**2a**) was prepared according to general procedure. The reaction of **1a**: (4-(tert-butyl) phenyl) boronic acid (89.1 mg, 0.5 mmol), 1,2-dinitrobenzene (1,2-DNB, acceptor) (0.025 mmol, 5 mol%, 4.2 mg) and Tri-*n*-Butylamine (donor) (0.5 mmol, 1.0 eq, 92.7 mg, 120 *u*L) in anhydrous MeCN (4 mL) under oxygen (1 atm) and blue LED illumination (450 nm) for 48 h. After that, flash column chromatography (eluent: PE/EtOAc = 9:1, v/v) affords 75.0 mg (99%) of **2a** as a white to pale yellow solid. **^{1}H NMR** (400 MHz, Chloroform-*d*) δ 7.29 – 7.21 (m, 2H), 6.80 – 6.74 (m, 2H), 1.29 (s, 9H). The ^{1}H NMR spectrum was in agreement with those reported in the reference.[1] **HRMS (ESI)** Calcd for [$C_{10}H_{13}O$, M - H]$^-$: 149.0966, Found: 149.0964.

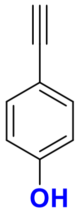

4-ethynylphenol (**2b**) was prepared according to general procedure. The reaction of **1b**: (4-ethynylphenyl)boronic acid (73.0 mg, 0.5 mmol), 1,2-dinitrobenzene (1,2-DNB, acceptor) (0.025 mmol, 5 mol%, 4.2 mg) and Tri-*n*-Butylamine (donor) (0.5 mmol, 1.0 eq, 92.7 mg, 120 *u*L) in anhydrous MeCN (4 mL) under oxygen (1 atm) and blue LED illumination (450 nm) for 48 h. After that, flash column chromatography (eluent: PE/EtOAc = 10:1, v/v) affords 18.1 mg (31%) of **2b** as a crimson solid. **^{1}H NMR** (400 MHz, DMSO-*d6*) δ 9.90 (s, 1H), 7.29 (d, *J* = 8.6 Hz, 2H), 6.75 (d, *J* = 8.6 Hz, 2H), 3.90 (s, 1H). **^{13}C NMR** (101 MHz, Chloroform-*d*) δ 158.56, 133.73, 116.07, 112.40, 84.46, 78.82. **HRMS (ESI)** Calcd for $[C_8H_5O, M - H]^-$: 117.0340, Found: 117.0342.

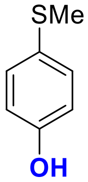

4-(methylthio)phenol (**2c**) was prepared according to general procedure. The reaction of **1c**: (4-(methylthio)phenyl)boronic acid (84.0 mg, 0.5 mmol), 1,2-dinitrobenzene (1,2-DNB, acceptor) (0.025 mmol, 5 mol%, 4.2 mg) and Tri-*n*-Butylamine (donor) (0.5 mmol, 1.0 eq, 92.7 mg, 120 *u*L) in anhydrous MeCN (4 mL) under oxygen (1 atm) and blue LED illumination (450 nm) for 48 h. After that, flash column chromatography (eluent: PE/EtOAc = 9:1, v/v) affords 44.1 mg (63%) of **2a** as a white to pale yellow solid. **^{1}H NMR** (400 MHz, Chloroform-*d*) δ 7.24 – 7.18 (m, 2H), 6.83 – 6.73 (m, 2H), 5.36 (s, 1H), 2.43 (s, 3H). **^{13}C NMR** (101 MHz, Chloroform-*d*) δ 154.08, 130.41, 128.81, 116.12, 18.05. **HRMS (ESI)** Calcd for $[C_7H_7OS, M - H]^-$: 139.0218, Found: 139.0216.

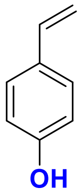

4-vinylphenol (**2d**) was prepared according to general procedure. The reaction of **1d**: (4-vinylphenyl)boronic acid (74.0 mg, 0.5 mmol), 1,2-dinitrobenzene (1,2-DNB, acceptor) (0.025 mmol, 5 mol%, 4.2 mg) and Tri-*n*-Butylamine (donor) (0.5 mmol, 1.0 eq, 92.7 mg, 120 *u*L) in

anhydrous MeCN (4 mL) under oxygen (1 atm) and blue LED illumination (450 nm) for 48 h. After that, flash column chromatography (eluent: PE/EtOAc = 10:1, v/v) affords 42.1 mg (70%) of **2d** as a white to pale yellow solid. **^{1}H NMR** (400 MHz, Chloroform-*d*) δ 7.34 – 7.27 (m, 2H), 6.82 – 6.76 (m, 2H), 6.65 (dd, *J* = 17.6, 10.9 Hz, 1H), 5.60 (dd, *J* = 17.6, 1.0 Hz, 1H), 5.12-4.96 (m, 2H). **^{13}C NMR** (101 MHz, Chloroform-*d*) δ 155.37, 136.20, 130.68, 127.64, 115.43, 111.66. **HRMS (ESI)** Calcd for [C_8H_7O, M - H]$^-$: 119.0497, Found: 119.0494.

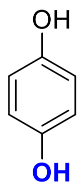

hydroquinone (**2e**) was prepared according to general procedure. The reaction of **1e**: (4-hydroxyphenyl)boronic acid (69.0 mg, 0.5 mmol), 1,2-dinitrobenzene (1,2-DNB, acceptor) (0.025 mmol, 5 mol%, 4.2 mg) and Tri-*n*-Butylamine (donor) (0.5 mmol, 1.0 eq, 92.7 mg, 120 *u*L) in anhydrous MeCN (4 mL) under oxygen (1 atm) and blue LED illumination (450 nm) for 48 h. After that, flash column chromatography (eluent: PE/EtOAc = 5:1, v/v) affords 28.6 mg (52%) of **2e** as a white to pale yellow solid. **^{1}H NMR** (400 MHz, Acetone-*d*$_6$) δ 7.64 (s, 2H), 6.66 (s, 4H). **^{13}C NMR** (101 MHz, Acetone-*d*$_6$) δ 151.18, 116.60. **HRMS (ESI)** Calcd for [$C_6H_5O_2$, M - H]$^-$: 109.0290, Found: 109.0289.

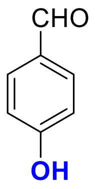

4-hydroxybenzaldehyde (**2f**) was prepared according to general procedure. The reaction of **1f**: (4-formylphenyl)boronic acid (75.0 mg, 0.5 mmol), 1,2-dinitrobenzene (1,2-DNB, acceptor) (0.025 mmol, 5 mol%, 4.2 mg) and Tri-*n*-Butylamine (donor) (0.5 mmol, 1.0 eq, 92.7 mg, 120 *u*L) in anhydrous MeCN (4 mL) under oxygen (1 atm) and blue LED illumination (450 nm) for 48 h. After that, flash column chromatography (eluent: PE/EtOAc = 6:1, v/v) affords 59.2 mg (97%) of **2f** as a white to pale yellow solid. **^{1}H NMR** (400 MHz, Chloroform-*d*) δ 9.86 (s, 1H), 7.88 – 7.69 (m, 2H), 7.04 – 6.83 (m, 2H), 6.47 (s, 1H). **^{13}C NMR** (101 MHz, Chloroform-*d*) δ 191.33, 162.43,

132.54, 129.51, 116.12. **HRMS (ESI)** Calcd for [$C_8H_5O_2$, M - H]$^-$: 121.0290, Found: 121.0288.

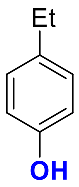

4-ethylphenol (**2g**) was prepared according to general procedure. The reaction of **1g**: (4-ethylphenyl)boronic acid (75.0 mg, 0.5 mmol), 1,2-dinitrobenzene (1,2-DNB, acceptor) (0.025 mmol, 5 mol%, 4.2 mg) and Tri-*n*-Butylamine (donor) (0.5 mmol, 1.0 eq, 92.7 mg, 120 *u*L) in anhydrous MeCN (4 mL) under oxygen (1 atm) and blue LED illumination (450 nm) for 48 h. After that, flash column chromatography (eluent: PE/EtOAc = 9:1, v/v) affords 50.7 mg (83%) of **2g** as a white to pale yellow solid. **^{1}H NMR** (400 MHz, Chloroform-*d*) δ 7.10 – 7.00 (dd, *J* = 8.4, 2.1 Hz, 2H), 6.76 (dd, *J* = 8.4, 2.1 Hz, 2H), 5.22 (s, 1H), 2.57 (q, *J* = 7.6 Hz, 2H), 1.19 (t, *J* = 7.6 Hz, 3H). **^{13}C NMR** (101 MHz, Chloroform-*d*) δ 153.34, 136.61, 128.94, 115.19, 27.99, 15.88. The ^{1}H NMR and ^{13}C NMR spectrum was in agreement with those reported in the reference.[1]

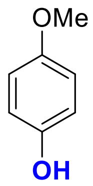

4-methoxyphenol (**2h**) was prepared according to general procedure. The reaction of **1h**: (4-methoxyphenyl)boronic acid (76.0 mg, 0.5 mmol), 1,2-dinitrobenzene (1,2-DNB, acceptor) (0.025 mmol, 5 mol%, 4.2 mg) and Tri-*n*-Butylamine (donor) (0.5 mmol, 1.0 eq, 92.7 mg, 120 *u*L) in anhydrous MeCN (4 mL) under oxygen (1 atm) and blue LED illumination (450 nm) for 48 h. After that, flash column chromatography (eluent: PE/EtOAc = 6:1, v/v) affords 40.4 mg (65%) of **2h** as a white to pale yellow solid. **^{1}H NMR** (400 MHz, Chloroform-*d*) δ 6.84 – 6.72 (m, 4H), 4.84 (s, 1H), 3.76 (s, 3H). **^{13}C NMR** (101 MHz, Chloroform-*d*) δ 153.75, 149.54, 116.09, 114.91, 55.85. **HRMS (ESI)** Calcd for [$C_7H_7O_2$, M - H]$^-$: 123.0446, Found: 123.0444.

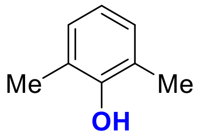

2,6-dimethylphenol (**2i**) was prepared according to general procedure. The reaction of **1i**: (2,6-dimethylphenyl)boronic acid (75.0 mg, 0.5 mmol), 1,2-dinitrobenzene (1,2-DNB, acceptor) (0.025 mmol, 5 mol%, 4.2 mg) and Tri-*n*-Butylamine (donor) (0.5 mmol, 1.0 eq, 92.7 mg, 120 *u*L) in anhydrous MeCN (4 mL) under oxygen (1 atm) and blue LED illumination (450 nm) for 48 h. After that, flash column chromatography (eluent: PE/EtOAc = 10:1, v/v) affords 35.5 mg (58%) of **2i** as a white to pale yellow solid. **^{1}H NMR** (400 MHz, Chloroform-*d*) δ 6.97 (d, *J* = 7.4 Hz, 2H), 6.75 (t, *J* = 7.5 Hz, 1H), 4.63 (s, 1H), 2.24 (s, 6H). **^{13}C NMR** (101 MHz, Chloroform-*d*) δ 152.18, 128.62, 122.99, 120.23, 15.87. The **HRMS (ESI)** Calcd for [C_8H_9O, M - H]$^-$: 121.0653, Found: 121.0648.

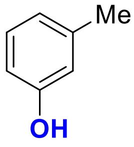

m-cresol (**2j**) was prepared according to general procedure. The reaction of **1j**: *m*-tolylboronic acid (68.0 mg, 0.5 mmol), 1,2-dinitrobenzene (1,2-DNB, acceptor) (0.025 mmol, 5 mol%, 4.2 mg) and Tri-*n*-Butylamine (donor) (0.5 mmol, 1.0 eq, 92.7 mg, 120 *u*L) in anhydrous MeCN (4 mL) under oxygen (1 atm) and blue LED illumination (450 nm) for 48 h. After that, flash column chromatography (eluent: PE/EtOAc = 10:1, v/v) affords 49.2 mg (91%) of **2j** as a white to pale yellow solid. **^{1}H NMR** (400 MHz, Chloroform-*d*) δ 7.11 (t, *J* = 7.6 Hz, 1H), 6.74 (d, *J* = 7.5 Hz, 1H), 6.70 – 6.61 (m, 2H), 5.29 (s, 1H), 2.29 (s, 3H). **^{13}C NMR** (101 MHz, Chloroform-*d*) δ 155.39, 139.86, 129.45, 121.65, 116.09, 112.34, 21.34. **HRMS (ESI)** Calcd for [C_7H_7O, M - H]$^-$: 107.0497, Found: 107.0494.

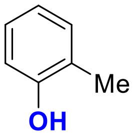

o-cresol (**2k**) was prepared according to general procedure. The reaction of **1k**: *o*-tolylboronic acid (68.0 mg, 0.5 mmol), 1,2-dinitrobenzene (1,2-DNB, acceptor) (0.025 mmol, 5 mol%, 4.2 mg) and Tri-*n*-Butylamine (donor) (0.5 mmol, 1.0 eq, 92.7 mg, 120 *u*L) in anhydrous

MeCN (4 mL) under oxygen (1 atm) and blue LED illumination (450 nm) for 48 h. After that, flash column chromatography (eluent: PE/EtOAc = 10:1, v/v) affords 40.6 mg (75%) of **2k** as a white to pale yellow solid. **^{1}H NMR** (400 MHz, Chloroform-*d*) δ 7.12 (dd, *J* = 7.4, 1.1 Hz, 1H), 7.07 (td, *J* = 7.6, 1.7 Hz, 1H), 6.84 (td, *J* = 7.4, 1.2 Hz, 1H), 6.76 (dd, *J* = 8.0, 1.2 Hz, 1H), 5.01 – 4.78 (s, 1H), 2.25 (s, 3H). **^{13}C NMR** (101 MHz, Chloroform-*d*) δ 153.79, 131.06, 127.14, 123.78, 120.76, 114.92, 15.73. The ^{1}H NMR and ^{13}C NMR spectrum was in agreement with those reported in the reference.[1]

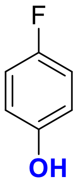

4-fluorophenol (**2l**) was prepared according to general procedure. The reaction of **1l**: (4-fluorophenyl)boronic acid (70 mg, 0.5 mmol), 1,2-dinitrobenzene (1,2-DNB, acceptor) (0.025 mmol, 5 mol%, 4.2 mg) and Tri-*n*-Butylamine (donor) (0.5 mmol, 1.0 eq, 92.7 mg, 120 *u*L) in anhydrous MeCN (4 mL) under oxygen (1 atm) and blue LED illumination (450 nm) for 48 h. After that, flash column chromatography (eluent: PE/EtOAc = 10:1, v/v) affords 52.7 mg (94%) of **2l** as a pale-yellow liquid. **^{1}H NMR** (400 MHz, Chloroform-*d*) δ 6.96 – 6.86 (m, 2H), 6.82 – 6.70 (m, 2H), 5.40 (s, 1H). **^{13}C NMR** (101 MHz, Chloroform-*d*) δ 157.28 (d, *J* = 237.7 Hz), 151.56 (d, *J* = 2.2 Hz), 116.27 (d, *J* = 8.0 Hz), 116.00 (d, *J* = 23.2 Hz). The ^{1}H NMR and ^{13}C NMR spectrum was in agreement with those reported in the reference.[2]

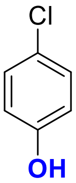

4-chlorophenol (**2m**) was prepared according to general procedure. The reaction of **1m**: (4-chlorophenyl)boronic acid (78.2 mg, 0.5 mmol), 1,2-dinitrobenzene (1,2-DNB, acceptor) (0.025 mmol, 5 mol%, 4.2 mg) and Tri-*n*-Butylamine (donor) (0.5 mmol, 1.0 eq, 92.7 mg, 120 *u*L) in anhydrous MeCN (4 mL) under oxygen (1 atm) and blue LED illumination (450 nm) for 48 h. After that, flash column chromatography (eluent: PE/EtOAc = 10:1, v/v) affords 45.7 mg (71%) of **2m** as a white to pale yellow

solid. **^{1}H NMR** (400 MHz, Chloroform-*d*) δ 7.23 – 7.11 (m, 2H), 6.81 – 6.69 (m, 2H), 5.43 (s, 1H). **^{13}C NMR** (101 MHz, Chloroform-*d*) δ 154.14, 129.55, 125.64, 116.70. **HRMS (ESI)** Calcd for [C_6H_4OCl, M - H]$^-$: 126.9951, Found: 126.9950.

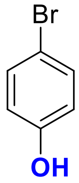

4-bromophenol (**2n**) was prepared according to general procedure. The reaction of **1n**: (4-bromophenyl)boronic acid (100.4 mg, 0.5 mmol), 1,2-dinitrobenzene (1,2-DNB, acceptor) (0.025 mmol, 5 mol%, 4.2 mg) and Tri-*n*-Butylamine (donor) (0.5 mmol, 1.0 eq, 92.7 mg, 120 *u*L) in anhydrous MeCN (4 mL) under oxygen (1 atm) and blue LED illumination (450 nm) for 48 h. After that, flash column chromatography (eluent: PE/EtOAc = 10:1, v/v) affords 86.4 mg (99%) of **2n** as a white to pale yellow solid. **^{1}H NMR** (400 MHz, Chloroform-*d*) δ 7.40 – 7.28 (m, 2H), 6.78 – 6.66 (m, 2H). **^{13}C NMR** (101 MHz, Chloroform-*d*) δ 154.68, 132.49, 117.24, 112.84. **HRMS (ESI)** Calcd for [C_6H_4BrO, M - H]$^-$: 170.9446, Found: 170.9443, 172.9420 (for ^{81}Br).

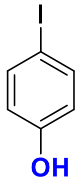

4-iodophenol (**2o**) was prepared according to general procedure. The reaction of **1o**: (4-iodophenyl)boronic acid (124.0 mg, 0.5 mmol), 1,2-dinitrobenzene (1,2-DNB, acceptor) (0.025 mmol, 5 mol%, 4.2 mg) and Tri-*n*-Butylamine (donor) (0.5 mmol, 1.0 eq, 92.7 mg, 120 *u*L) in anhydrous MeCN (4 mL) under oxygen (1 atm) and blue LED illumination (450 nm) for 48 h. After that, flash column chromatography (eluent: PE/EtOAc = 10:1, v/v) affords 97.9 mg (89%) of **2o** as a white to pale yellow solid. **^{1}H NMR** (400 MHz, Chloroform-*d*) δ 7.53 – 7.49 (m, 2H), 6.65-6.61 (m, 2H), 5.04 (s, 1H). **^{13}C NMR** (101 MHz, Chloroform-*d*) δ 155.40, 138.45, 117.82, 82.66. **HRMS (ESI)** Calcd for [C_6H_4IO, M - H]$^-$: 218.9307, Found: 218.9310.

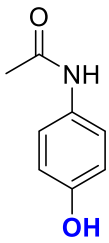

N-(4-hydroxyphenyl)acetamide (**2p**) was prepared according to general procedure. The reaction of **1p**: 4-acetylaminophenylboronic acid pinacol ester (130.6 mg, 0.5 mmol), 1,2-dinitrobenzene (1,2-DNB, acceptor) (0.025 mmol, 5 mol%, 4.2 mg) and Tri-*n*-Butylamine (donor) (0.5 mmol, 1.0 eq, 92.7 mg, 120 *u*L) in anhydrous MeCN (4 mL) under oxygen (1 atm) and blue LED illumination (450 nm) for 48 h. After that, flash column chromatography (eluent: PE/EtOAc = 1:2, v/v) affords 46.9 mg (62%) of **2p** as a white to pale yellow solid. **^{1}H NMR** (400 MHz, DMSO-*d6*) δ 9.63 (s, 1H), 9.11 (s, 1H), 7.33 (d, *J* = 8.7 Hz, 2H), 6.67 (d, *J* = 8.6 Hz, 2H), 1.97 (s, 3H). **^{13}C NMR** (101 MHz, DMSO-*d6*) δ 167.5, 153.1, 131.02, 120.8, 115.0, 23.7. **HRMS (ESI)** Calcd for [$C_8H_8NO_2$, M - H]$^-$: 150.0555, Found: 150.0556.

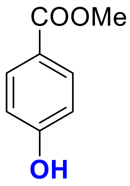

methyl 4-hydroxybenzoate (**2r**) was prepared according to general procedure. The reaction of **1q**: (4-(methoxycarbonyl)phenyl)boronic acid (99.0 mg, 0.5 mmol), 1,2-dinitrobenzene (1,2-DNB, acceptor) (0.025 mmol, 5 mol%, 4.2 mg) and Tri-*n*-Butylamine (donor) (0.5 mmol, 1.0 eq, 92.7 mg, 120 *u*L) in anhydrous MeCN (4 mL) under oxygen (1 atm) and blue LED illumination (450 nm) for 48 h. After that, flash column chromatography (eluent: PE/EtOAc = 8:1, v/v) affords 71.5 mg (94%) of **2q** as a white to pale yellow solid. **^{1}H NMR** (400 MHz, Chloroform-*d*) δ 8.00 – 7.90 (m, 2H), 6.94 – 6.83 (m, 2H), 6.49 (s, 1H), 3.90 (s, 3H). **^{13}C NMR** (101 MHz, Chloroform-*d*) δ 167.41, 160.34, 131.99, 122.34, 115.33, 52.09. **HRMS (ESI)** Calcd for [$C_8H_7O_3$, M - H]$^-$: 151.0395, Found: 151.0393.

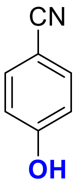

4-hydroxybenzonitrile (**2s**) was prepared according to general procedure. The reaction of **1r**: (4-cyanophenyl)boronic acid (73.5 mg, 0.5 mmol), 1,2-dinitrobenzene (1,2-DNB, acceptor) (0.025 mmol, 5 mol%, 4.2 mg) and Tri-*n*-Butylamine (donor) (0.5 mmol, 1.0 eq, 92.7 mg, 120 *u*L) in

anhydrous MeCN (4 mL) under oxygen (1 atm) and blue LED illumination (450 nm) for 48 h. After that, flash column chromatography (eluent: PE/EtOAc = 6:1, v/v) affords 59.2 mg (99%) of **2r** as a white to pale yellow solid. **^{1}H NMR** (400 MHz, Chloroform-*d*) δ 7.71 – 7.41 (m, 2H), 7.06 – 6.89 (m, 2H). **^{13}C NMR** (101 MHz, Chloroform-*d*) δ 160.29, 134.36, 119.30, 116.50, 102.98. The ^{1}H NMR and ^{13}C NMR spectrum was in agreement with those reported in the reference.[1] **HRMS (ESI)** Calcd for $[C_7H_4NO, M - H]^-$: 118.0293, Found: 118.0292.

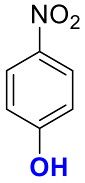

4-nitrophenol (**2t**) was prepared according to general procedure. The reaction of **1s**: (4-nitrophenyl)boronic acid (83.5 mg, 0.5 mmol), 1,2-dinitrobenzene (1,2-DNB, acceptor) (0.025 mmol, 5 mol%, 4.2 mg) and Tri-*n*-Butylamine (donor) (0.5 mmol, 1.0 eq, 92.7 mg, 120 *u*L) in anhydrous MeCN (4 mL) under oxygen (1 atm) and blue LED illumination (450 nm) for 48 h. After that, flash column chromatography (eluent: PE/EtOAc = 8:1, v/v) affords 45.5 mg (65%) of **2s** as a pale-yellow solid. **^{1}H NMR** (400 MHz, Chloroform-*d*) δ 8.28 – 8.07 (m, 2H), 7.03 – 6.81 (m, 2H). **^{13}C NMR** (101 MHz, Chloroform-*d*) δ 161.87, 141.44, 126.33, 115.79. **HRMS (ESI)** Calcd for $[C_6H_4NO_3, M - H]^-$: 138.0191, Found: 138.0189.

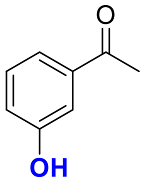

1-(3-hydroxyphenyl)ethan-1-one (**2u**) was prepared according to general procedure. The reaction of **1t**: (3-acetylphenyl)boronic acid (82.0 mg, 0.5 mmol), 1,2-dinitrobenzene (1,2-DNB, acceptor) (0.025 mmol, 5 mol%, 4.2 mg) and Tri-*n*-Butylamine (donor) (0.5 mmol, 1.0 eq, 92.7 mg, 120 *u*L) in anhydrous MeCN (4 mL) under oxygen (1 atm) and blue LED illumination (450 nm) for 48 h. After that, flash column chromatography (eluent: PE/EtOAc = 10:1, v/v) affords 68.1 mg (99%) of **2t** as a white to pale yellow solid. **^{1}H NMR** (400 MHz, Chloroform-*d*) δ 7.59 – 7.44 (m, 2H), 7.33 (t, *J* = 7.9 Hz, 1H), 7.12 (ddd, *J* = 8.1, 2.6, 1.0 Hz, 1H), 7.08 – 6.88

(m, 1H), 2.60 (s, 3H). ^{13}**C NMR** (101 MHz, Chloroform-*d*) δ 199.31, 156.53, 138.36, 129.91, 120.96, 120.88, 114.80, 26.80. **HRMS (ESI)** Calcd for [$C_8H_7O_2$, M - H]$^-$: 135.0446, Found: 135.0445.

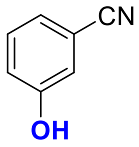

3-hydroxybenzonitrile (**2v**) was prepared according to general procedure. The reaction of **1u**: (3-cyanophenyl)boronic acid (73.5 mg, 0.5 mmol), 1,2-dinitrobenzene (1,2-DNB, acceptor) (0.025 mmol, 5 mol%, 4.2 mg) and Tri-*n*-Butylamine (donor) (0.5 mmol, 1.0 eq, 92.7 mg, 120 *u*L) in anhydrous MeCN (4 mL) under oxygen (1 atm) and blue LED illumination (450 nm) for 48 h. After that, flash column chromatography (eluent: PE/EtOAc = 8:1, v/v) affords 55.9 mg (94%) of **2u** as a white to pale yellow solid. ^{1}**H NMR** (400 MHz, Chloroform-*d*) δ 7.35 (t, *J* = 7.9 Hz, 1H), 7.23 (dt, *J* = 7.6, 1.3 Hz, 1H), 7.19 – 7.05 (m, 2H), 6.41 (s, 1H). ^{13}**C NMR** (101 MHz, Chloroform-*d*) δ 156.33, 130.66, 124.51, 120.90, 118.79, 118.64, 112.64. **HRMS (ESI)** Calcd for [C_7H_4NO, M - H]$^-$: 118.0293, Found: 118.0291.

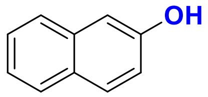

naphthalen-2-ol (**2w**) was prepared according to general procedure. The reaction of **1v**: naphthalen-2-ylboronic acid (86.0 mg, 0.5 mmol), 1,2-dinitrobenzene (1,2-DNB, acceptor) (0.025 mmol, 5 mol%, 4.2 mg) and Tri-*n*-Butylamine (donor) (0.5 mmol, 1.0 eq, 92.7 mg, 120 *u*L) in anhydrous MeCN (4 mL) under oxygen (1 atm) and blue LED illumination (450 nm) for 48 h. After that, flash column chromatography (eluent: PE/EtOAc = 10:1, v/v) affords 55.6 mg (77%) of **2v** as a white to pale yellow solid. ^{1}**H NMR** (400 MHz, Chloroform-*d*) δ 7.74 (t, *J* = 8.6 Hz, 2H), 7.66 (d, *J* = 8.2 Hz, 1H), 7.41 (ddd, *J* = 8.2, 6.8, 1.3 Hz, 1H), 7.32 (ddd, *J* = 8.2, 6.8, 1.3 Hz, 1H), 7.17 – 7.01 (m, 2H), 5.31 (s, 1H). ^{13}**C NMR** (101 MHz, Chloroform-*d*) δ 153.33, 134.61, 129.89, 128.97, 127.80, 126.56, 126.40, 123.66, 117.78, 109.56. **HRMS (ESI)** Calcd for [$C_{10}H_7O$, M - H]$^-$: 143.0497, Found: 143.0495.

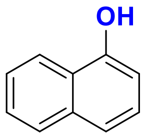

naphthalen-1-ol (**2x**) was prepared according to general procedure. The reaction of **1w**: naphthalen-1-ylboronic acid (86.0 mg, 0.5 mmol), 1,2-dinitrobenzene (1,2-DNB, acceptor) (0.025 mmol, 5 mol%, 4.2 mg) and Tri-*n*-Butylamine (donor) (0.5 mmol, 1.0 eq, 92.7 mg, 120 *u*L) in anhydrous MeCN (4 mL) under oxygen (1 atm) and blue LED illumination (450 nm) for 48 h. After that, flash column chromatography (eluent: PE/EtOAc = 10:1, v/v) affords 39.7 mg (55%) of **2w** as a white to pale yellow solid. **^{1}H NMR** (400 MHz, Chloroform-*d*) δ 8.22 – 8.14 (m, 1H), 7.86 – 7.77 (m, 1H), 7.55 – 7.40 (m, 3H), 7.30 (dd, *J* = 8.3, 7.4 Hz, 1H), 6.81 (dd, *J* = 7.5, 1.0 Hz, 1H), 5.33 (s, 1H). **^{13}C NMR** (101 MHz, Chloroform-*d*) δ 151.40, 134.80, 127.71, 126.47, 125.87, 125.29, 124.39, 121.56, 120.71, 108.66. **HRMS (ESI)** Calcd for [$C_{10}H_7O$, M - H]$^-$: 143.0497, Found: 143.0497.

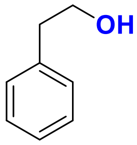

2-phenylethan-1-ol (**2y**) was prepared according to general procedure. The reaction of **1x**: phenethylboronic acid (75.0 mg, 0.5 mmol), 1,2-dinitrobenzene (1,2-DNB, acceptor) (0.025 mmol, 5 mol%, 4.2 mg) and Tri-*n*-Butylamine (donor) (0.5 mmol, 1.0 eq, 92.7 mg, 120 *u*L) in anhydrous MeCN (4 mL) under oxygen (1 atm) and blue LED illumination (450 nm) for 48 h. After that, flash column chromatography (eluent: PE/EtOAc = 3:1, v/v) affords 36.1 mg (59%) of **2x** as a white to pale yellow solid. **^{1}H NMR** (400 MHz, Chloroform-*d*) δ 7.35 – 7.27 (m, 2H), 7.23 (t, *J* = 6.9 Hz, 3H), 3.84 (t, *J* = 6.6 Hz, 2H), 2.86 (t, *J* = 6.6 Hz, 2H), 1.68 (s, 1H). **^{13}C NMR** (101 MHz, Chloroform-*d*) δ 138.51, 129.05, 128.59, 126.48, 63.67, 39.20. The ^{1}H NMR and ^{13}C NMR spectrum was in agreement with those reported in the reference.[3]

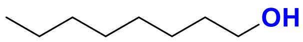

octan-1-ol (**2z**) was prepared according to general procedure. The reaction of **1y**: octylboronic acid (79.1 mg, 0.5 mmol), 1,2-dinitrobenzene (1,2-DNB, acceptor) (0.025 mmol, 5 mol%, 4.2 mg) and Tri-*n*-

Butylamine (donor) (0.5 mmol, 1.0 eq, 92.7 mg, 120 *u*L) in anhydrous MeCN (4 mL) under oxygen (1 atm) and blue LED illumination (450 nm) for 48 h. After that, flash column chromatography (eluent: PE/EtOAc = 5:1, v/v) affords 52.7 mg (81%) of **2y** as a white to pale yellow solid. **^{1}H NMR** (400 MHz, Chloroform-*d*) δ 3.64 (t, *J* = 6.7 Hz, 2H), 1.56 (q, *J* = 6.9 Hz, 2H), 1.47 – 1.22 (m, 11H), 0.88 (t, *J* = 6.5 Hz, 3H). **^{13}C NMR** (101 MHz, Chloroform-*d*) δ 63.10, 32.83, 31.85, 29.43, 29.31, 25.77, 22.69, 14.12. The ^{1}H NMR and ^{13}C NMR spectrum was in agreement with those reported in the reference.[4]

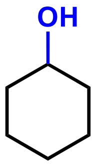

cyclohexanol (**2ab**) was prepared according to general procedure and using CD_3CN as solvent. After complete the reaction, the reaction system does not require post-processing and proceed directly with sampling for nuclear magnetic resonance (NMR) analysis. **^{1}H NMR** (400 MHz, CD_3CN) δ 3.54 (m, *1*H), 1.89 (m, 2H), 1.76 (m, 2H), 1.58 (m, 2H), 1.32 – 1.12 (m, 5H). **^{13}C NMR** (101 MHz, CD_3CN) δ 70.24, 36.32, 26.40, 24.99. The ^{1}H NMR and ^{13}C NMR spectrum was in agreement with those reported in the reference.[8]

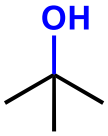

tert butyl alcohol (**2ac**) was prepared according to general procedure and using CD_3CN as solvent. After complete the reaction, the reaction system does not require post-processing and proceed directly with sampling for nuclear magnetic resonance (NMR) analysis. **^{1}H NMR** (400 MHz, CD_3CN) 1.16 (s, 9H). **^{13}C NMR** (101 MHz, CD_3CN) δ 65.26, 30.07.

The ^{1}H NMR and ^{13}C NMR spectrum was in agreement with those reported in the reference.[9]

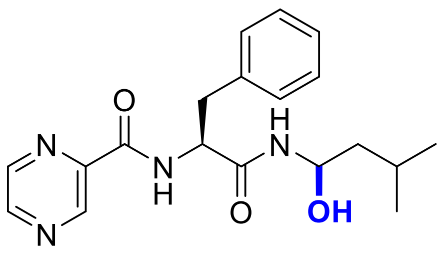

N-((S)-1-(((R)-1-hydroxy-3-methylbutyl) amino)-1-oxo-3-phenylpropan-2-yl)pyrazine-2-carboxamide (**4a**) was prepared according to general procedure. The reaction of Bortezomib (192.1 mg, 0.5 mmol): 1,2-dinitrobenzene (1,2-DNB, acceptor) (0.025 mmol, 5 mol%, 4.2 mg) and Tri-*n*-Butylamine (donor) (0.5 mmol, 1.0 eq, 92.7 mg, 120 *u*L) in anhydrous MeCN (6 mL) under oxygen (1 atm) and blue LED illumination (450 nm) for 24 h. After that, flash column chromatography (eluent: PE/EtOAc = 1:1, v/v) affords 119.7 mg (67%) of **4a** as a pale yellow solid. **^{1}H NMR** (400 MHz, Chloroform-*d*) δ 9.31 (s, *1*H), 8.76 (m, *1*H), 8.54 (s, *1*H), 8.45 (d, *J* = 8.3 Hz, *1*H), 7.22 (m, *5*H), 7.07 (d, *J* = 7.6 Hz, *1*H), 5.39 (q, *J* = 7.0 Hz, *1*H), 4.94 (q, *J* = 7.5 Hz, *1*H), 3.22 (d, *J* = 7.0 Hz, *1*H), 1.28 (m, *3*H), 0.85 (d, *J* = 6.6 Hz, *6*H). **^{13}C NMR** (101 MHz, Chloroform-*d*) δ 171.08, 162.93, 147.43, 144.14, 143.75, 142.77, 136.05, 129.31, 128.54, 127.02, 73.09, 54.44, 43.91, 38.31, 24.14, 22.65, 22.01. **HRMS (ESI)** Calcd for $[NaC_{19}H_{24}N_4O_3^{+}$, M + Na$]^{+}$: 379.1741, Found: 379.1747.

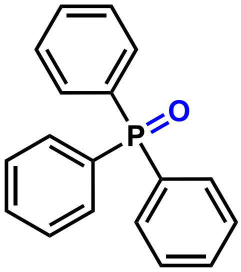

Triphenylphosphine oxide (**5a**) was prepared according to general procedure. The reaction of triphenylphosphine (131.1 mg, 0.5 mmol), 1,2-dinitrobenzene (1,2-DNB, acceptor) (0.025 mmol, 5 mol%, 4.2 mg) and Tri-*n*-Butylamine (donor) (0.5 mmol, 1.0 eq, 92.7 mg, 120 *u*L) in anhydrous MeCN (4 mL) under oxygen (1 atm) and blue LED illumination (450

nm) for 48 h. After that, flash column chromatography (eluent: PE/EtOAc = 1.6:1, v/v) affords 139.0 mg (99%) of **5a** as a white solid. **^{1}H NMR** (400 MHz, Chloroform-*d*) δ 7.69 – 7.63 (m, 6H), δ 7.57 – 7.49 (m, 3H), δ 7.48 – 7.37 (m, 6H). **^{13}C NMR** (101 MHz, Chloroform-*d*) δ 133.09, 132.15, 132.05, 131.95, 128.57, 128.45. The ^{1}H NMR and ^{13}C NMR spectrum was in agreement with those reported in the reference.[7]

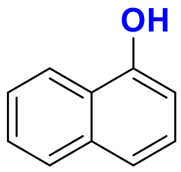

naphthalen-1-ol (**6a**) was prepared according to general procedure. The reaction of trimethoxy(naphthalen-1-yl)silane (124.2 mg, 0.5 mmol), 1,2-dinitrobenzene (1,2-DNB, acceptor) (0.025 mmol, 5 mol%, 4.2 mg) and Tri-*n*-Butylamine (donor) (0.5 mmol, 1.0 eq, 92.7 mg, 120 *u*L) in anhydrous MeCN (4 mL) under oxygen (1 atm) with NH_4F (27.8 mg, 1.5 eq) as additive and blue LED illumination (450 nm) for 48 h. After that, flash column chromatography (eluent: PE/EtOAc = 10:1, v/v) affords 36.8 mg (51%) of **6a** as a white to pale yellow solid. (*Note:* The weighing should be conducted in a dry environment to prevent ammonium fluoride from absorbing moisture and deliquescing, which could affect the yield.) **^{1}H NMR** (400 MHz, Chloroform-*d*) δ 8.22 – 8.14 (m, 1H), 7.86 – 7.77 (m, 1H), 7.55 – 7.40 (m, 3H), 7.30 (dd, *J* = 8.3, 7.4 Hz, 1H), 6.81 (dd, *J* = 7.5, 1.0 Hz, 1H), 5.33 (s, 1H). **^{13}C NMR** (101 MHz, Chloroform-*d*) δ 151.40, 134.80, 127.71, 126.47, 125.87, 125.29, 124.39, 121.56, 120.71, 108.66. **HRMS (ESI)** Calcd for $[C_{10}H_7O, M - H]^-$: 143.0497, Found: 143.0497.

(E) Mechanistic Studies

Characterization EDA Complex:

Equimolar amounts (0.025 M) of pale yellow 1,2-dinitrobenzene and colorless tri-n-butylamine were mixed in acetonitrile solvent, resulting in a pronounced darkening of the solution:

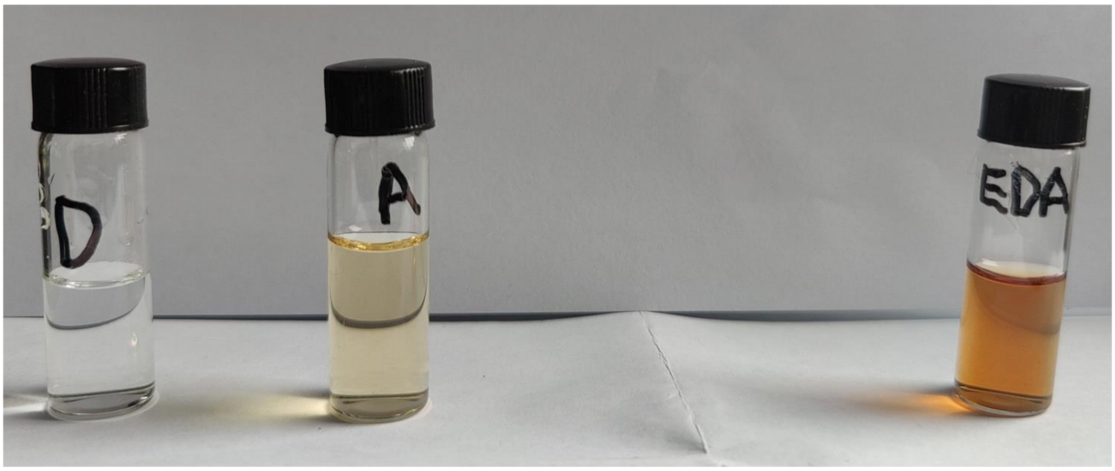

Supplementary Figure 7. Picture of D (Donor, left), A (Acceptor, middle) and EDA (Donor + Acceptor, right) in MeCN solution with concentration of 0.025 M.

UV-Vis absorption spectra were recorded using a Cary Eclipse Agilent Technologies Absorption spectrometer using 1 cm path quartz cuvettes.

UV-visible absorption spectrum measurement (absorption spectra were recorded in MeCN in 1 cm path quartz cuvettes) revealed a significant bathochromic displacement (shift towards longer wavelengths) and increased absorption of visible light in MeCN solution with concentration of 0.025 M (Supplementary Figure 8):

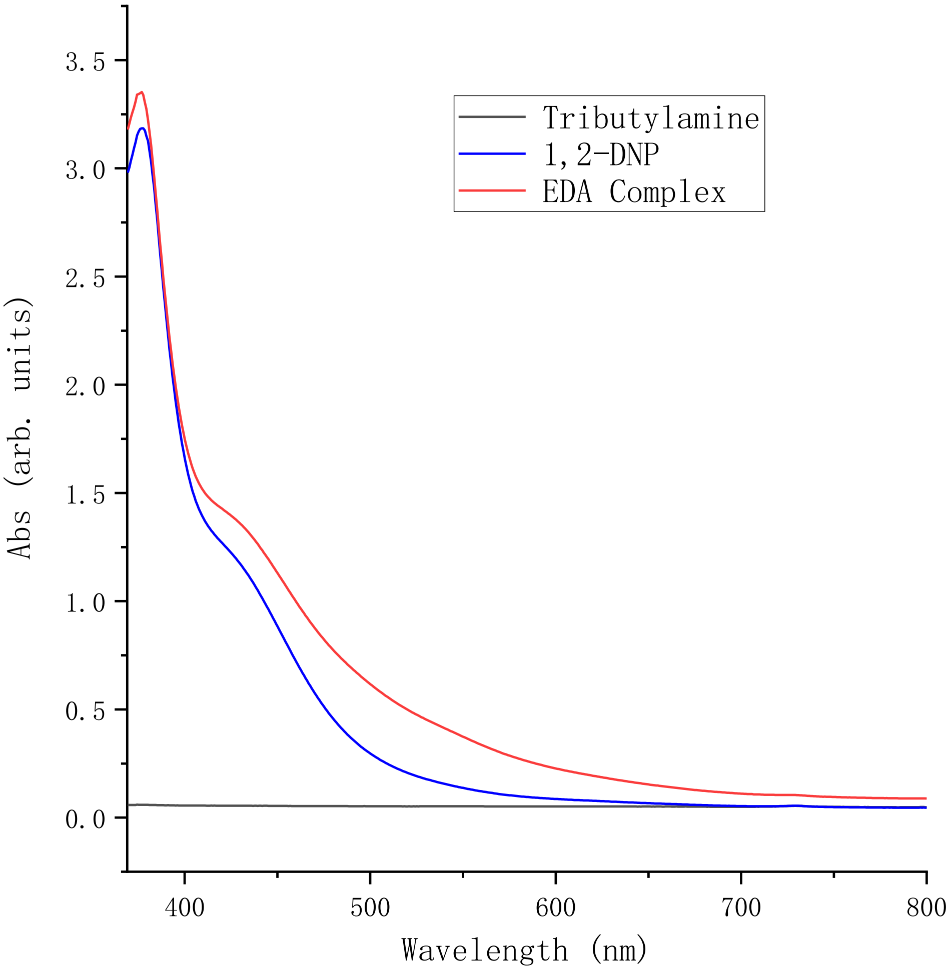

Supplementary Figure 8. Ultraviolet visible absorption spectrum of D (Donor, gray line), A (Acceptor, blue line) and EDA Complex (red line).

While ensuring the final concentration of the Acceptor remains constant (0.025 M), as the proportion of the Donor increases, the absorbance of the entire system gradually increases, showing a phenomenon of initially rapid and then slower rate of increase (see Supplementary Figure 9). Once the Donor and Acceptor reach an equimolar ratio, the absorbance no longer shows significant

increase in MeCN solution with concentration of 0.025 M (absorption spectra were recorded in MeCN in 1 cm path quartz cuvettes):

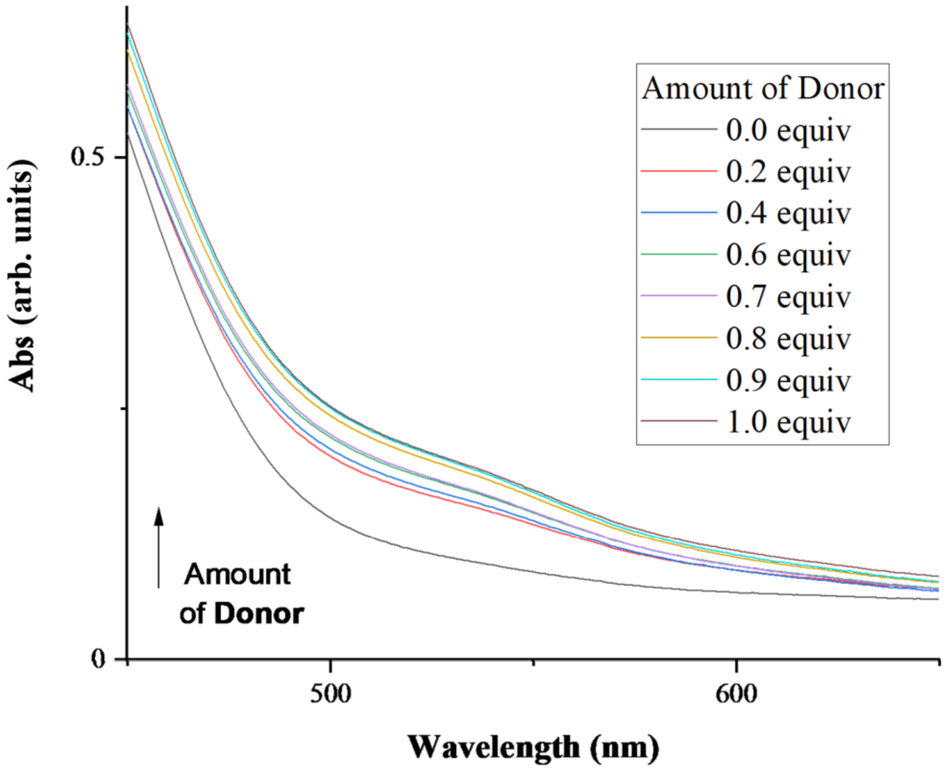

Supplementary Figure 9. Ultraviolet visible absorption spectrum of Donor and the EDA Complex with the proportion of the Donor increases.

It can be observed that the maximum increment in absorbance of the EDA Complex is reached at 490 nm (Supplementary Figure 10):

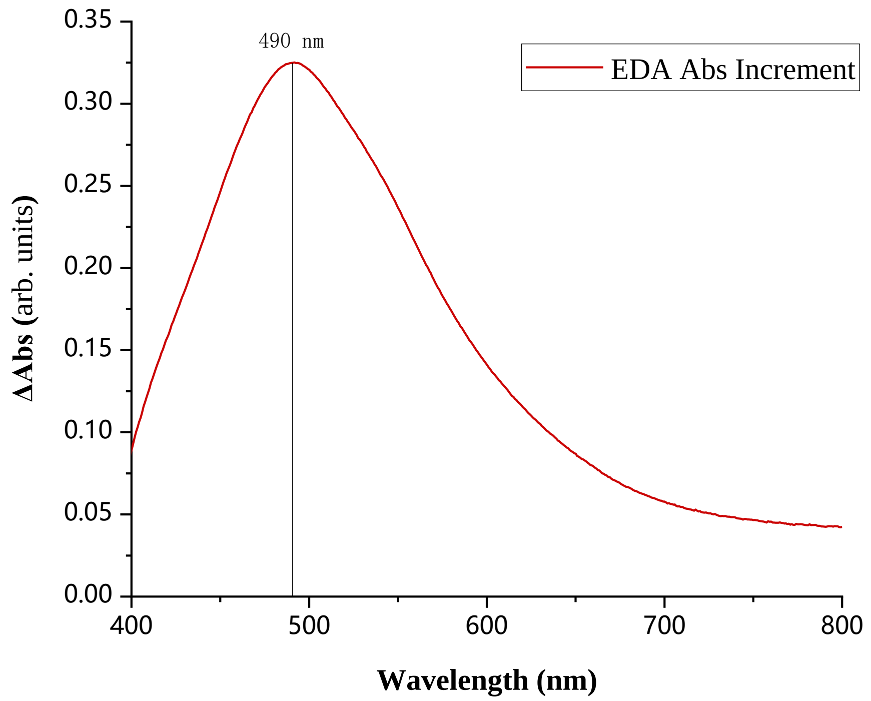

Supplementary Figure 10. Increment in absorbance of the EDA Complex.

Using this wavelength as the reference point, by measuring the absorbance increment of the EDA Complex at different ratios, we conducted a Job's Plot (see Supplementary Figure 11) and surprisingly found that the optimal binding ratio between the Acceptor and Donor is 4:1:

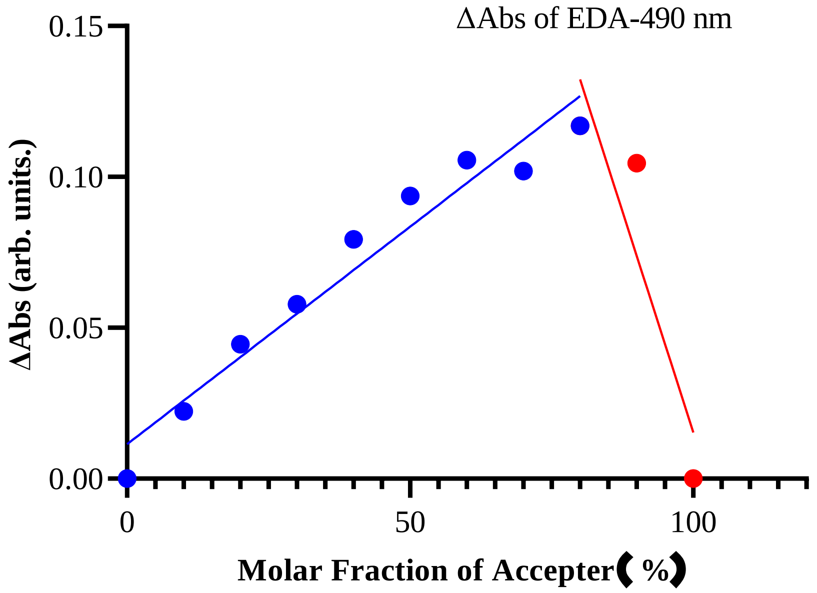

Supplementary Figure 11. Job’s Plot.

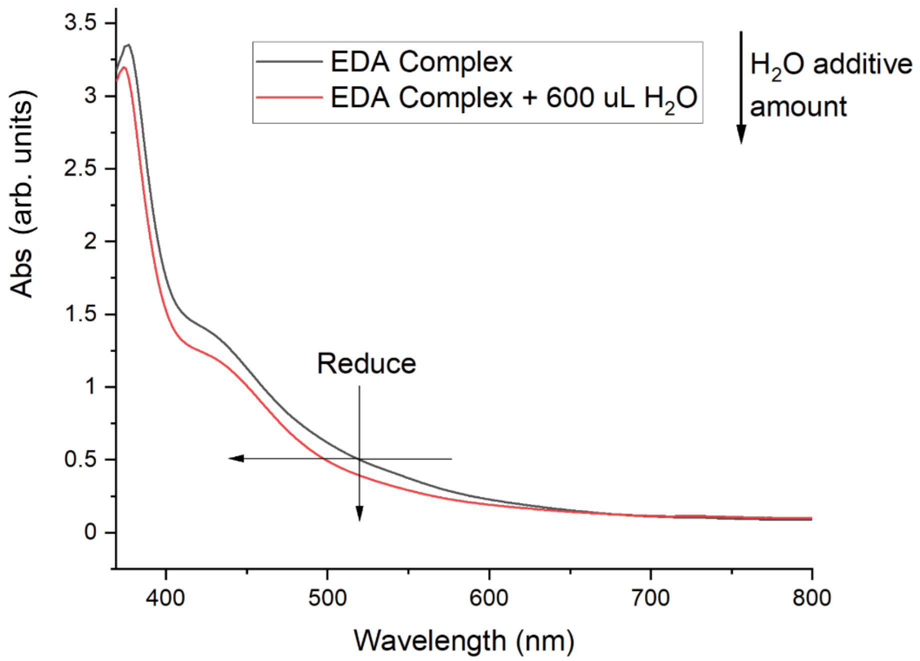

Supplementary Figure 12. Ultraviolet visible absorption spectrum of EDA Complex in MeCN solution with concentration of 0.025 M and 600 *u*Lwater as additive.

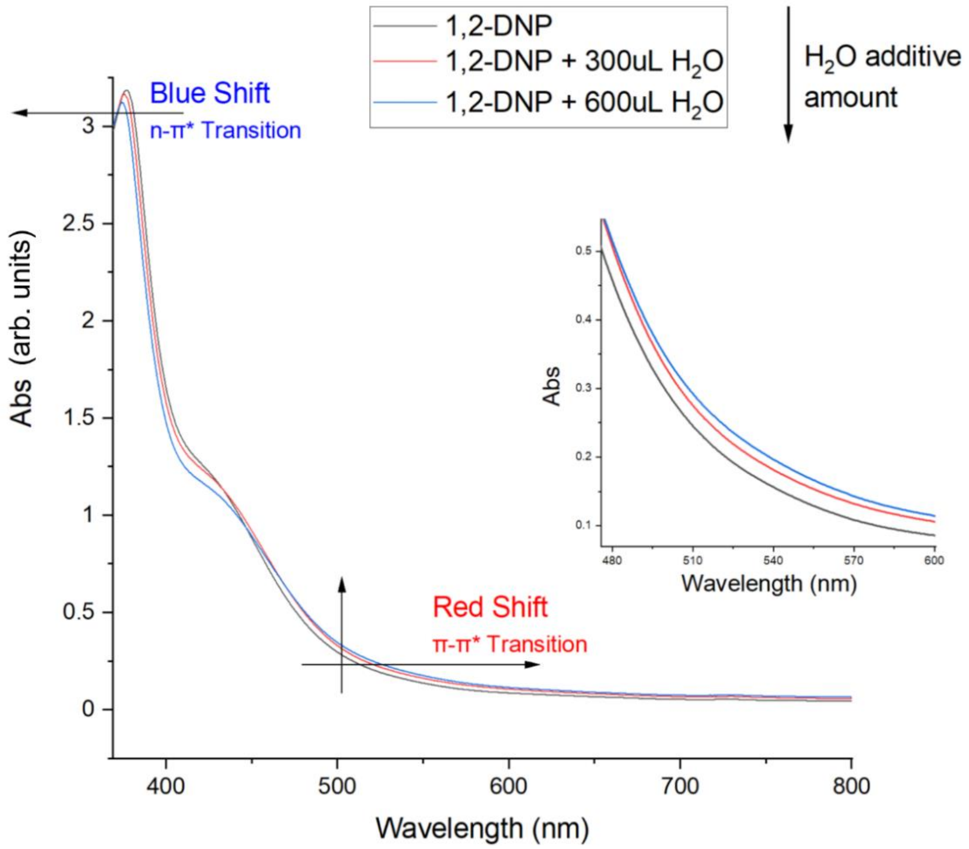

Supplementary Figure 13. Ultraviolet visible absorption spectrum of Donor in MeCN solution with concentration of 0.025 M and 600 *u*L water as additive.

Excluding the dilution effect caused by water addition: a separate addition of water to another acceptor solution in same amount and concentration showed a slight red shift in visible absorption due to increased solvent polarity in π-π* transition of nitroaromatics (see Supplementary Figure 13). It was speculated that water acted as both a strong hydrogen bond donor and acceptor, disrupting the formation of the EDA Complex. This observation also explained why the addition of 600 microliters of water completely suppressed the oxygenation reaction under standard conditions (absorption spectra were recorded in MeCN in 1 cm path quartz cuvettes).

Fluorescence spectra were obtained with a Cary Eclipse Agilent Technologies fluorescence spectrophotometer using 1 cm path quartz cuvettes.

Detection of ROS:

We used ROS fluorescent probe DCFH (2,7-Dichlorodihydrofluorescein) to detect the existence of ROS. According to specification, the excitation wavelength is 485 nm and the positive emission wavelength is 525 nm.

DCFH is a commonly used and classical fluorescent probe for detecting reactive oxygen species (ROS).[5] In this study, we prepared a stock solution of DCFH in acetonitrile with a concentration of 10 mM, which was stored at a temperature of minus 20 degrees Celsius. During the testing process, the Donor and Acceptor solutions were added, and the volume was adjusted with acetonitrile to maintain a final concentration of 0.025 M for the EDA Complex. Simultaneously, the concentration of DCFH probe was maintained at 1 mM. Throughout the experiment, except for the use of Blue LED light source for illumination, all steps were conducted in the absence of light. Immediately after adding the fluorescent probe to the system, the curve 0 was obtained, and subsequently, measurements were taken every 10 seconds after 1-minute intervals of light exposure (450 nm Blue LED, see Supplementary Figure 1, left). This allowed us to obtain various fluorescence emission curves Supplementary Figure 14. (Fluorescence emission spectrum was recorded in MeCN in 1 cm path quartz cuvettes):

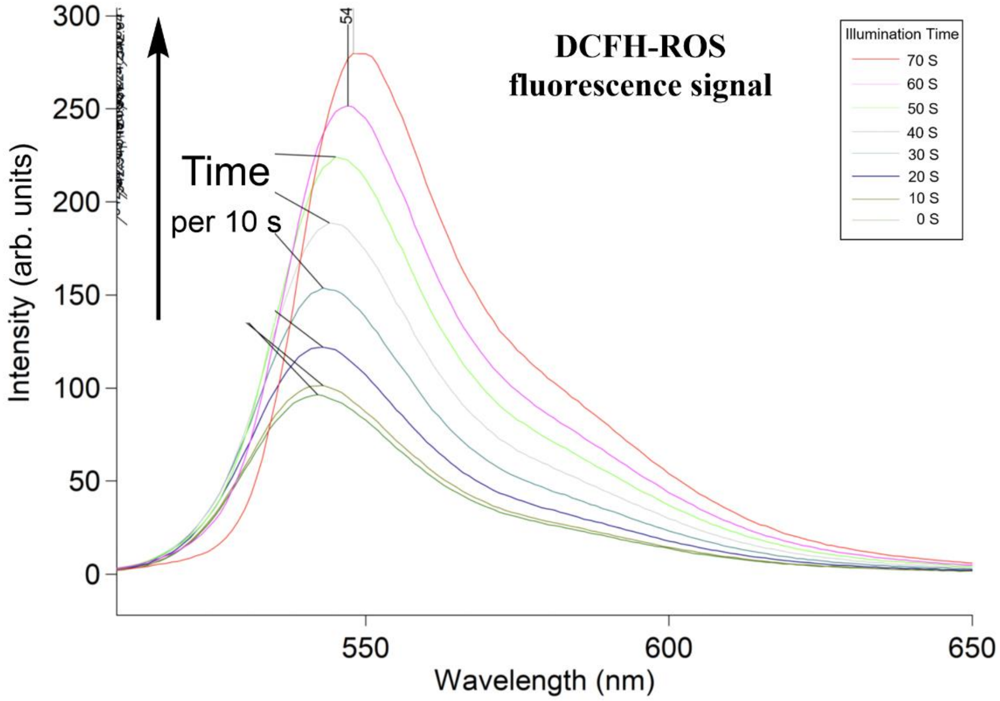

Supplementary Figure 14. DCFH ROS fluorescent probe positive fluorescence signal with extended illumination time.

Detection of superoxide:

Similarly, we employed dihydroethidium (DHE) as a specific probe for detecting superoxide species.[6] The fluorescence signal of the probe was measured before and 12 hours after light exposure (fluorescence emission spectrum was recorded in MeCN in 1 cm path quartz cuvettes). Before light exposure, the system was prepared by adding the DHE probe solution. The concentration of DHE used was 0.1 mM. The sample was then incubated for a specified period to allow the probe to react with superoxide species present in the system. Following the incubation period, the fluorescence signal of the probe was measured using fluorescence spectrophotometer to obtain the baseline fluorescence spectrum and intensity. After the baseline measurement, the system was subjected to light exposure (450 nm Blue LED, see Supplementary Figure 1, right) for a duration of 12 hours. The light source used was Blue LED.

The light exposure conditions were optimized to stimulate the generation of superoxide species. After the 12-hour light exposure, the sample was again subjected to fluorescence measurement using the same technique as before. By comparing the fluorescence signal obtained before and after light exposure, we were able to evaluate the changes in the fluorescence intensity, which provided insights into the generation and behavior of superoxide species in the system. This test was conducted based on relevant literature references. The experimental procedure and methodology were adapted from previously published studies in the field. By referring to these sources, we ensured that our experimental setup and procedures were consistent with established practices and standards in the scientific community. The literature references provided guidance on the selection of DHE as a specific probe for detecting superoxide species and the appropriate concentration to use. They also helped us determine the optimal conditions for the probe to react with the superoxide species present in the system. Furthermore, the literature references guided us in designing the light exposure protocol and selecting the appropriate duration and light source. This ensured that our experimental setup aligned with previously validated approaches for stimulating the generation of superoxide species. By incorporating the knowledge and insights from the relevant literature, we were able to conduct a rigorous and scientifically sound investigation into the fluorescence signal of the DHE probe before and after the designated light exposure period.

It is worth mentioning that after determining the spectrum of the fluorescent probe DHE, it is necessary to use mass spectrometry to detect the oxygenated products to confirm its specificity for superoxide detection. In our study, we successfully detected the corresponding positive result, which is the superoxide-oxygenated DHE product, through mass spectrometry. The use of mass spectrometry is a powerful analytical technique that can provide valuable information about the chemical composition and structure of molecules. In the

case of DHE, mass spectrometry analysis allows us to identify and verify the specific reaction product formed when it interacts with superoxide species. By confirming the presence of the superoxide-oxygenated DHE product through mass spectrometry, we have further substantiated the specificity of DHE as a probe for detecting superoxide species. This validation step enhances the reliability and confidence in the results obtained from the fluorescence measurements and strengthens the overall credibility of our experimental findings (see Supplementary Figure 15 – 17).

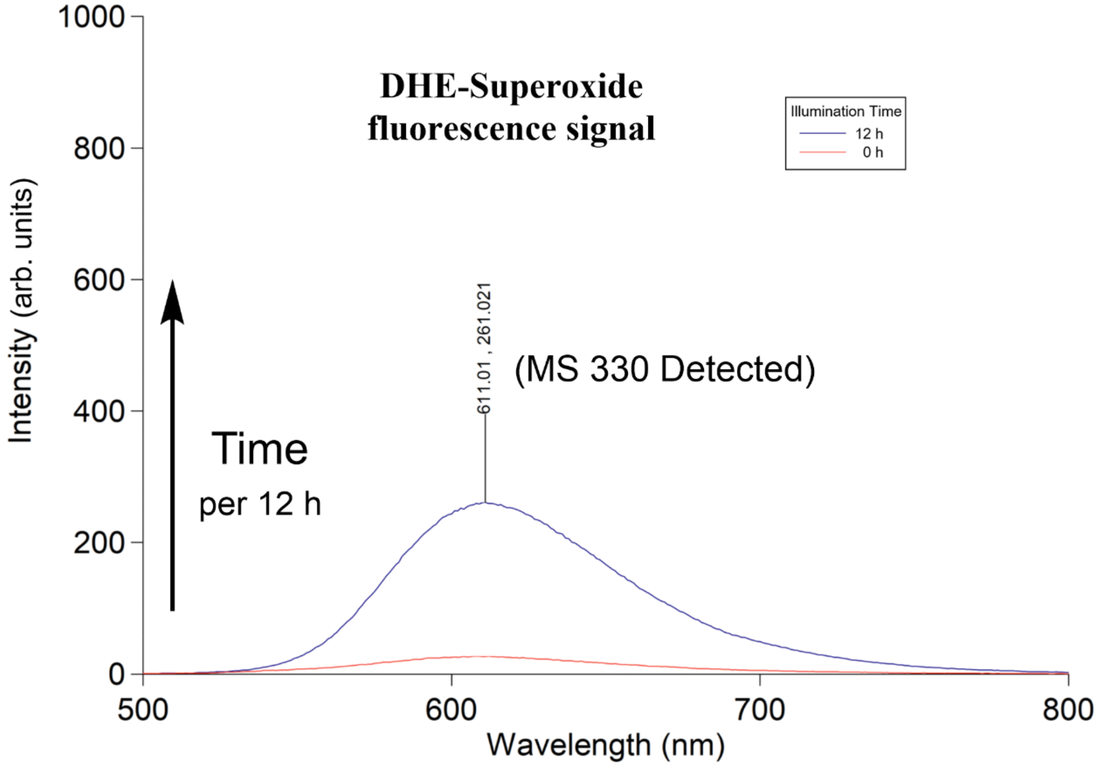

Supplementary Figure 15. DHE fluorescent probe positive fluorescence signal with extended illumination time.

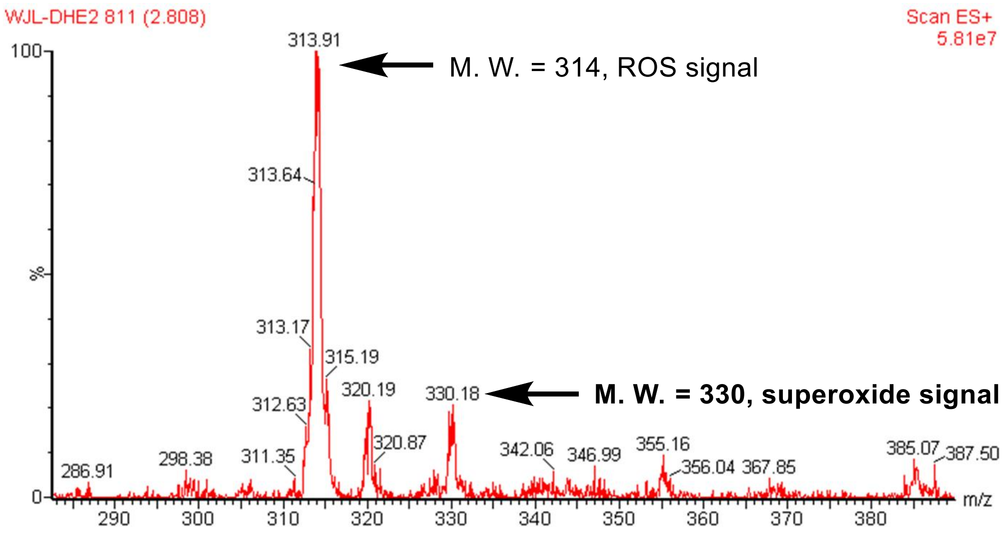

Supplementary Figure 16. Mass spectrum Analysis of DHE Fluorescent Probe after Light Exposure.

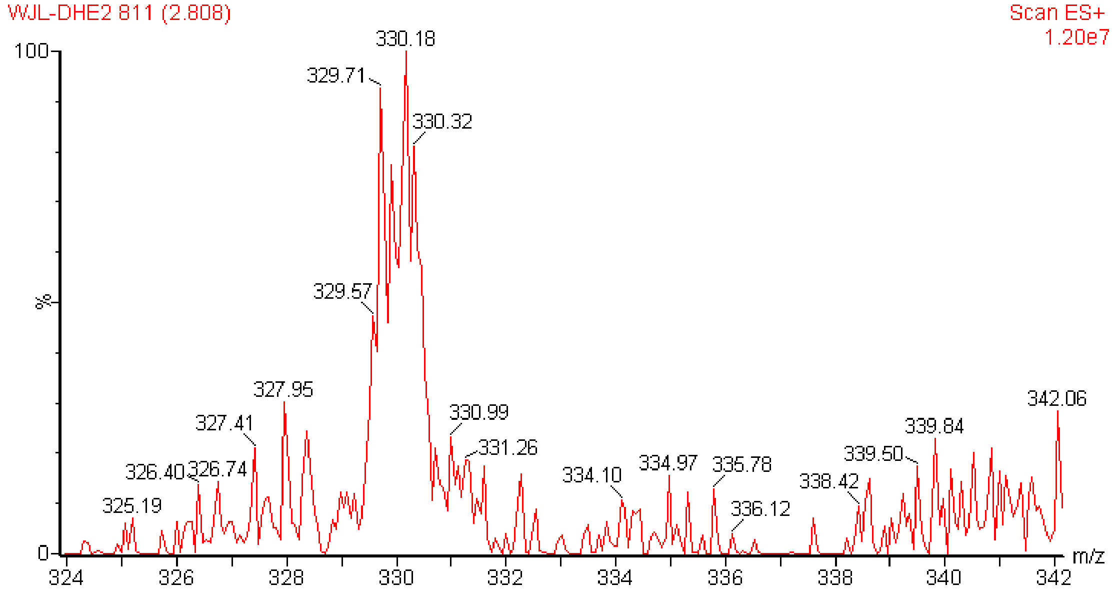

Supplementary Figure 17. Mass spectrum amplification of DHE Fluorescent Probe after Light Exposure.

As for amines used as sacrificial reagents in typical photoredox catalytic systems, their type (steric hindrance) generally has little impact on the yield. This is because the excited state photocatalyst has very high activity, and its redox ability is greatly enhanced due to the rearrangement of electrons in the molecular orbitals in the excited state. Therefore, various types of amines and even other types of reducing agents can serve as sacrificial reducing agents as long as the redox potentials are mutually compatible[10-13]. However, in the EDA Complex photocatalytic system, due to the electron-rich amine compounds acting as Donors, the process of forming EDA complexes with electron-deficient acceptor is extremely sensitive to steric hindrance: The reaction yields corresponding to donors with similar redox potentials[14,15], but differing steric hindrances vary significantly. (See Supplementary Figure 18 and Supplementary Table 4. Optimization of Electron Donor in SI). The yield of the reaction is sensitive to the steric hindrance of the amine rather than to the redox potential of the amine, further corroborating the mechanism of the EDA Complex photocatalytic system we proposed.

(*Tips:* There are numerous literature reports on amine compounds acting as reductive quenchers, such as: THIQ[16]; Secondary amines[17]. Et_3N[18]; *n*-Bu_3N[19]; TPA[20]; DIPEA[21].)

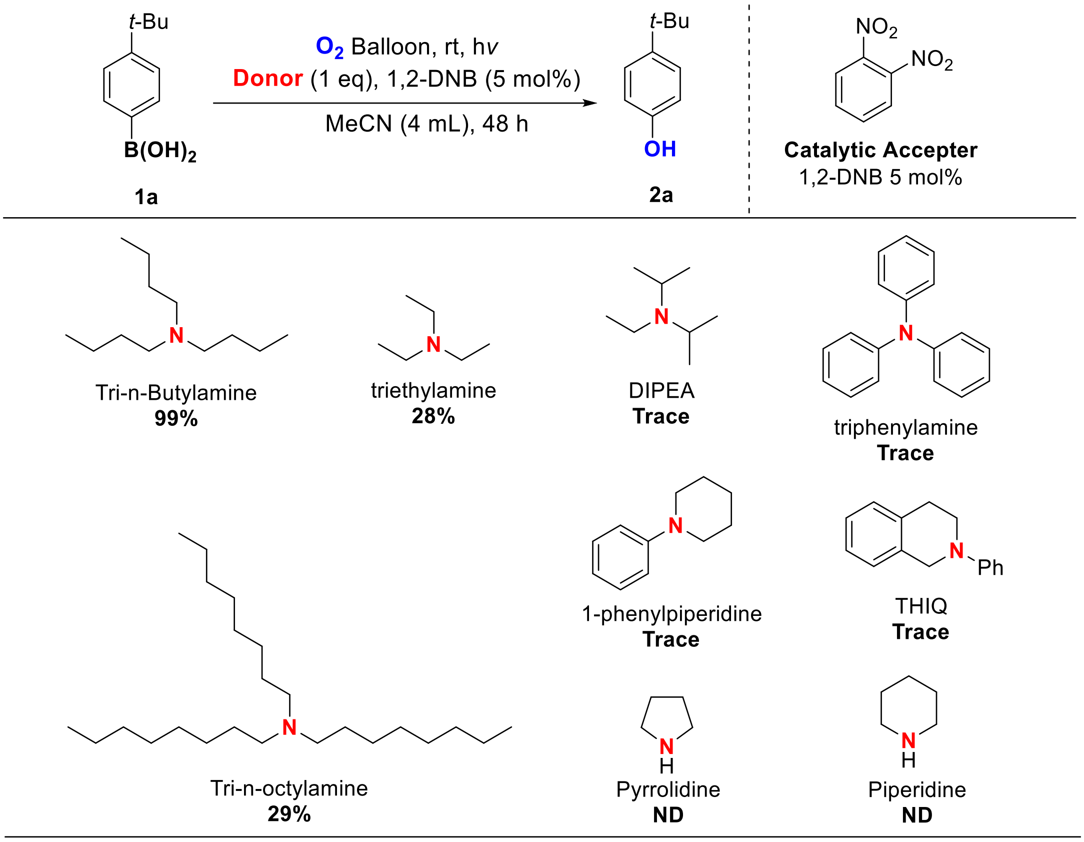

Supplementary Figure 18. Yields corresponding to donors with similar redox potentials but different steric hindrances.

It is also feasible to use a light source that possesses an optical profile that does not coincide with any local band. For example, 1,3-dinitrobenzene (1,3-DNB) has almost no absorption in the visible light region, especially no absorbency near the wavelength of *450 nm* of our light source used. However, upon adding the Donor, the formation of the EDA complex results in the emergence of new absorption bands (visible absorption, especially around 450 nm), please see Supplementary Figure 19. The absorbance of these new bands increases with the addition of the Donor (reflecting the dynamic equilibrium of Donor-Acceptor binding, and the addition of 20 times the amount of Donor to the Acceptor used as a catalyst also corresponds to the actual usage ratio of the relevant compounds: 5 mol% vs 1.0 eq). It was further supported that the reaction with 1,3-dinitrobenzene instead of 1,2-dinitrobenzene as the Acceptor produced the corresponding phenol product in 61% (Supplementary Figure 20).

These results demonstrate the rationality of the EDA Complex photocatalytic system we proposed.

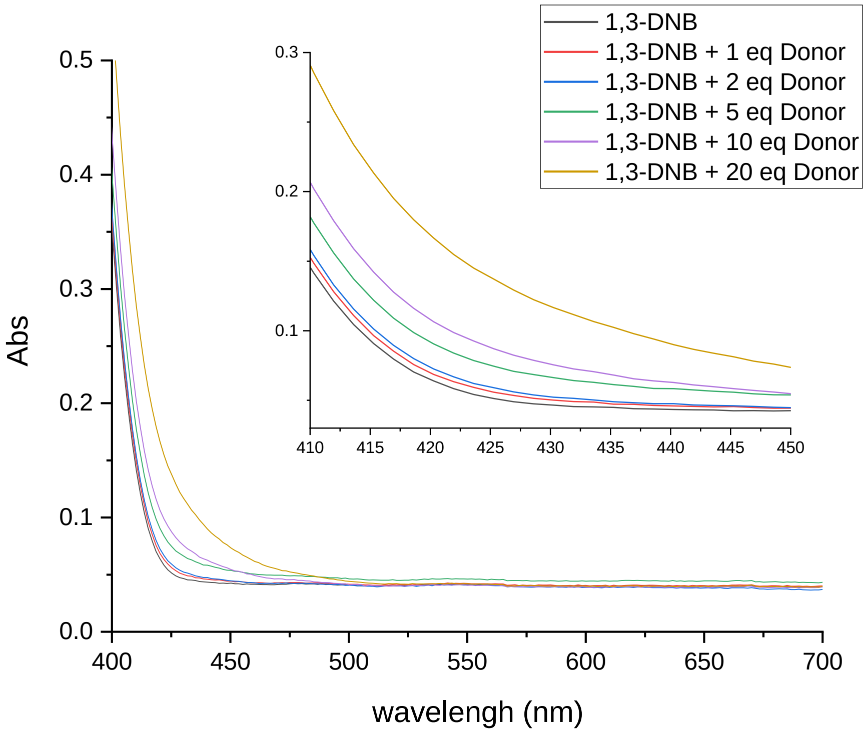

Supplementary Figure 19. 1,3-DNB and the corresponding absorption bands of EDA complex

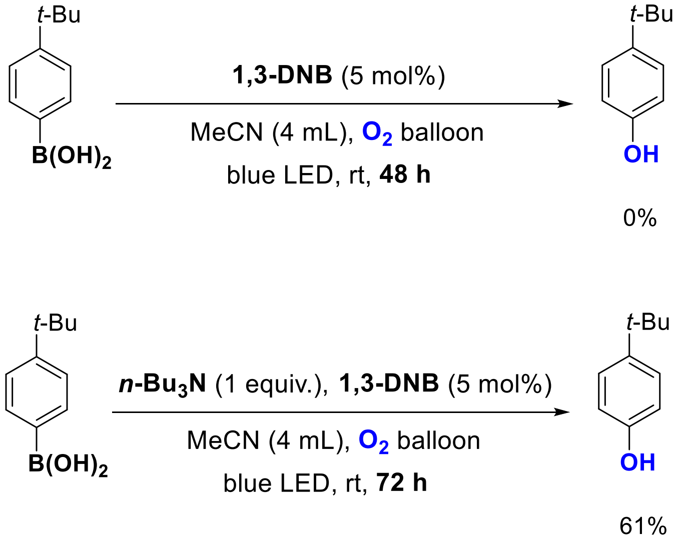

Supplementary Figure 20. The efficiency of the EDA complex catalytic reaction corresponding to 1,3-DNB.

Supplementary Table 4. Control Experiments of 1,3-DNB as catalytic acceptor[a]

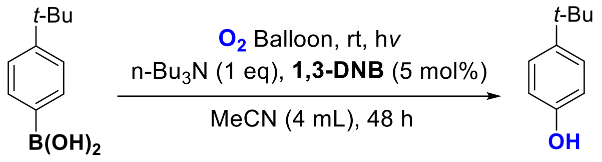

1a **2a**

entry	changed conditions	Isolated Yield
**1**	**none**	**34%**
2	72 h reaction time	**61%**
3	No donor	0%
4	No acceptor	0%
5	Under dark	0%
6	Under Ar	0%
7	600 *u*L H_2O as additive	Trace [b]

[a] Reaction conditions: **1a** (0.5 mmol), Tri-*n*-Butylamine donor (0.5 mmol), acceptor (5 mol%) in solvent (4.0 mL) under oxygen (1 atm). [b] Water, as a strong hydrogen bond donor-acceptor, can disrupt the EDA Complex when introduced into the system, thereby inhibiting the reaction.

Stern-Volmer analysis:

To enhance the importance of this research, we conducted a Stern-Volmer analysis, and the results are as follows:

(Testing conditions: Using above Agilent fluorescence spectrophotometer, after instrument tuning, the excitation light source grating slit width was increased to 20 and the excitation voltage strength set to 730, resulting in the following spectrum. Measurements were made using a 1 cm quartz fluorescence cuvette.)

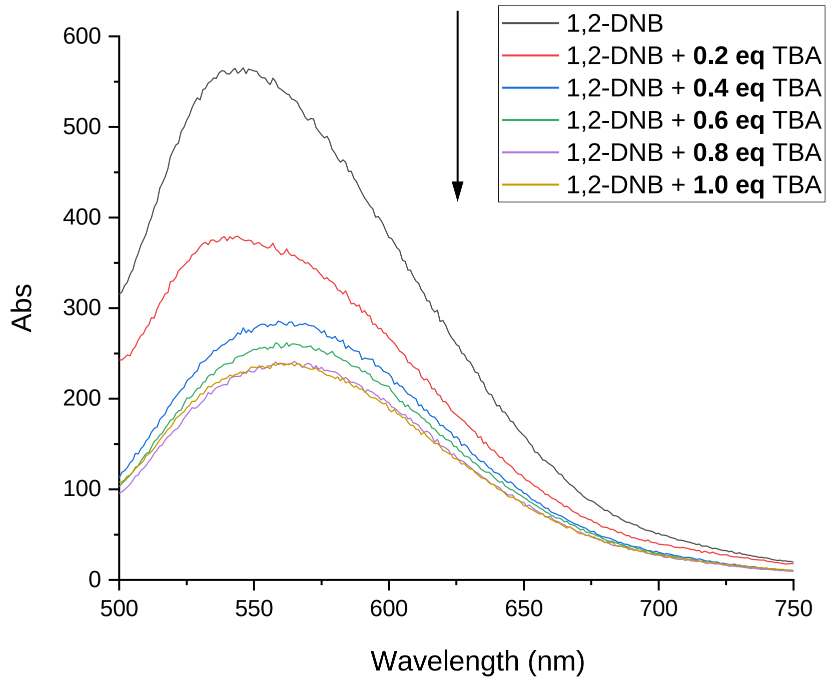

Supplementary Figure 21. Stern-Volmer quenching experiment

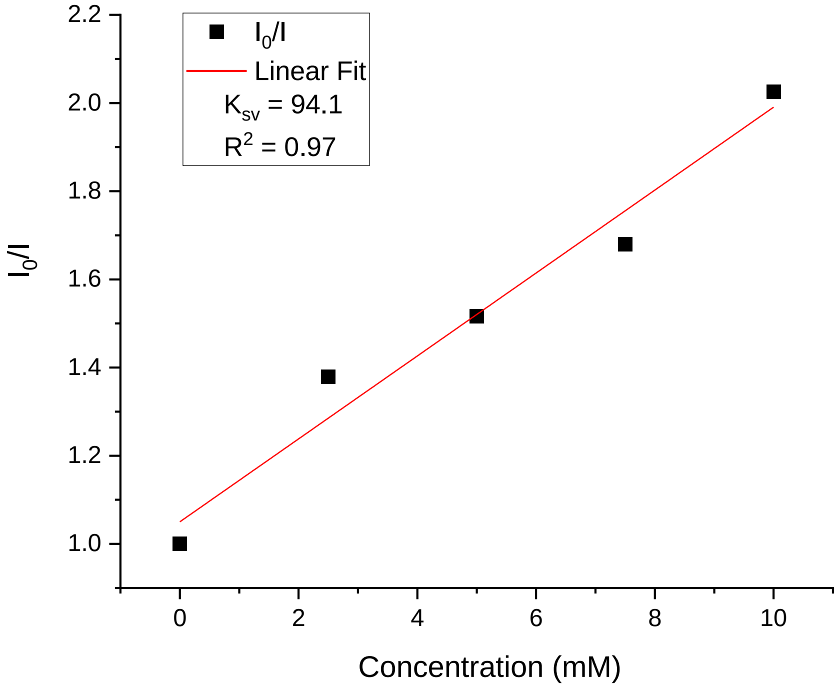

Supplementary Figure 22. Stern-Volmer quenching experiment linear fit

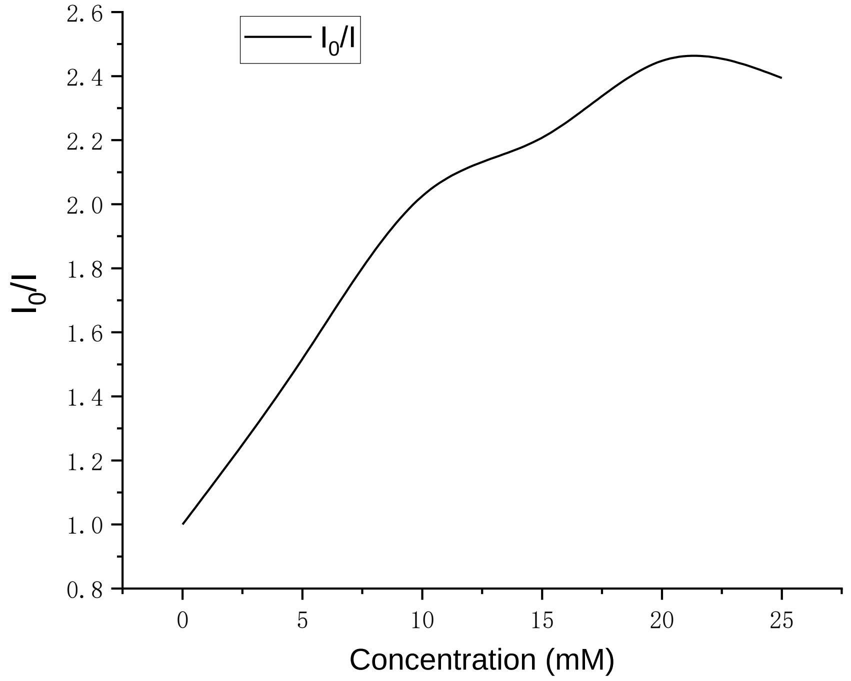

Supplementary Figure 23. The fluorescence quenching curve with extended concentration.

It is noteworthy that as the amount of the donor increases, the fluorescence quenching curve no longer remains linear. This is related to the coordination saturation of the EDA Complex: with the addition of the donor, EDA Complexes are continuously formed, rapidly quenching the luminescence of the acceptor. However, as the amount of the donor increases further, the formation of the EDA Complex gradually reaches saturation, resulting in a decrease and eventual leveling off of the slope of the quenching curve (Supplementary Figure 23). This observation aligns with the mechanism proposed in our study.

Cyclic Voltammetry (CV) experiments:

The results of the Cyclic Voltammetry (CV) experiment are as follows (Supplementary Figure 24 – 27):

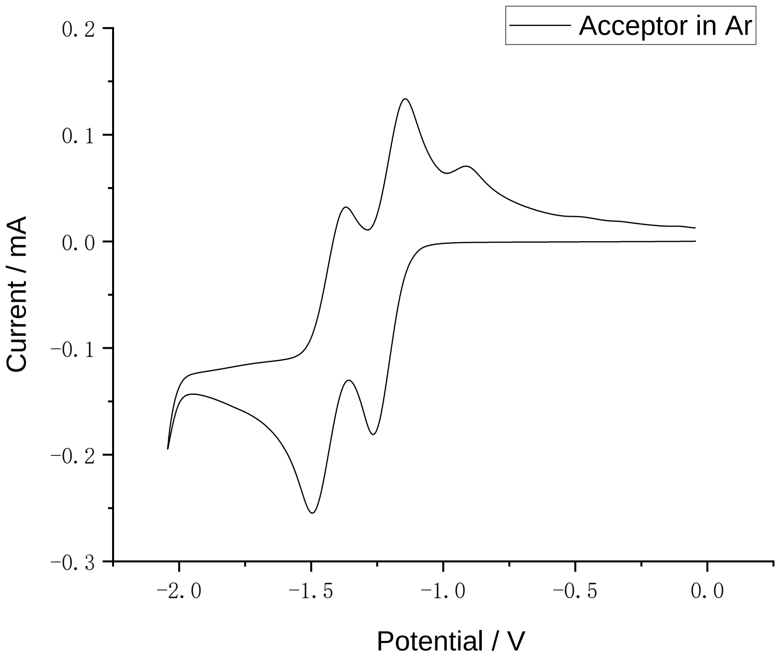

Supplementary Figure 24. CV of Acceptor 1,2-DNB

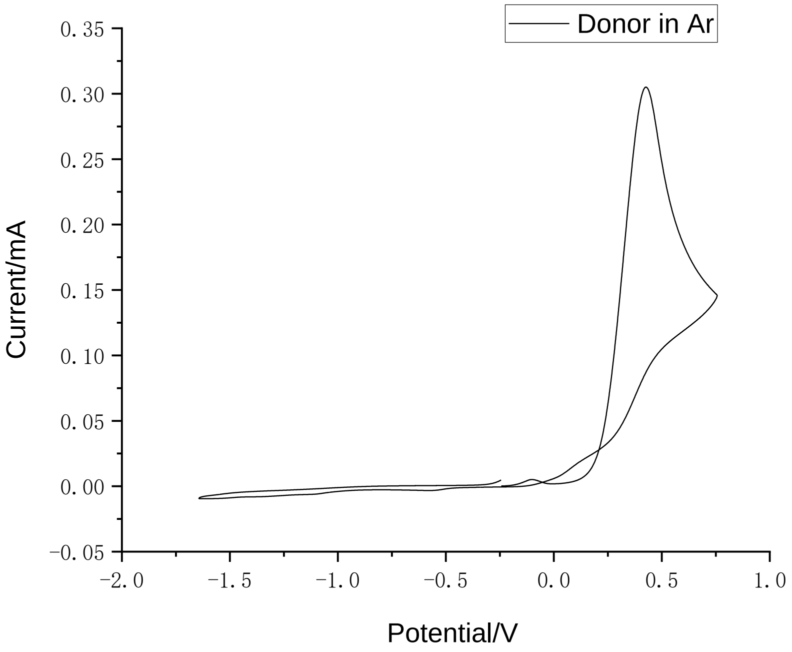

Supplementary Figure 25. CV of Donor TBA

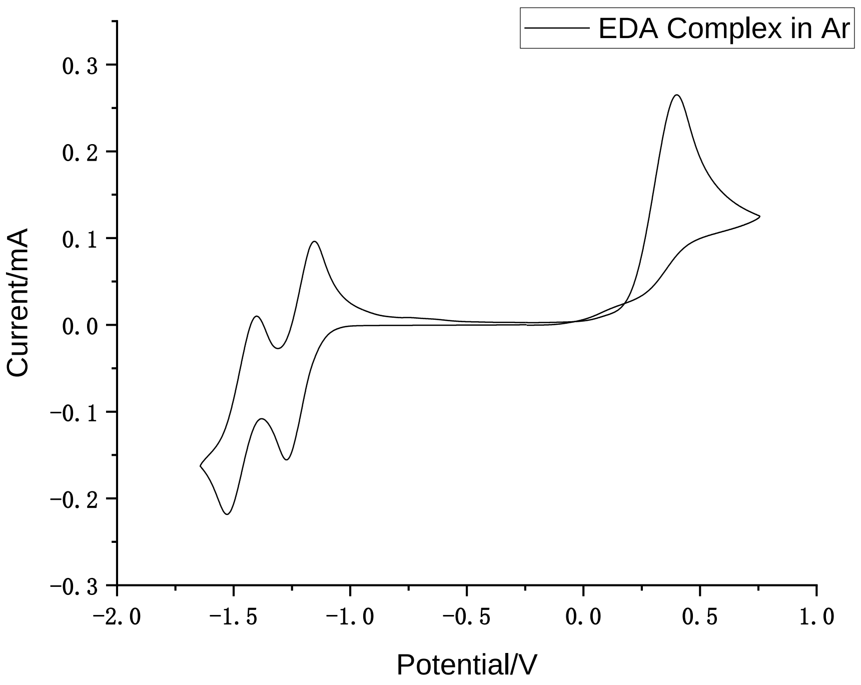

Supplementary Figure 26. CV of EDA Complex

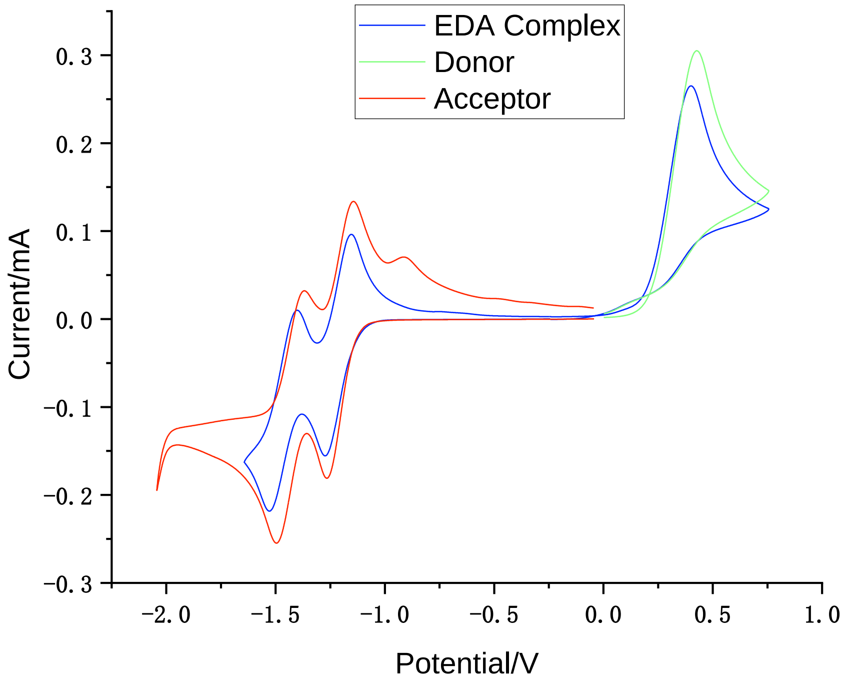

Supplementary Figure 27. Merged CV of EDA Complex together with Donor and Acceptor

Analysis and discussion of the CV experimental results: The redox potential of the EDA Complex shifted to the left (towards a lower potential) compared to the isolated Donor and Acceptor (Supplementary Figure 27). This shift is attributed to the formation of the EDA Complex, where the electron clouds of the Donor and Acceptor molecules become polarized due to their interaction. This polarization causes an elevation in the energy level of the Donor's HOMO (Highest Occupied Molecular Orbital), making it more easily oxidizable. Concurrently, once the Acceptor molecule combines with the electron-rich Donor, the resultant EDA Complex is more inclined towards intracomplex electron transfer, thus becoming less receptive to the reduction by external electrons.

Note: All electrode potentials have been calibrated using the ferrocene standard potential and all data were measured three times and data were accepted only after they stabilized and showed no further fluctuations to ensure accuracy.

Testing conditions: The concentrations of both the Donor and Acceptor were 0.025 M, the solvent used was anhydrous acetonitrile, and the electrolyte was recrystallized *n*-Bu_4NPF_6.

Quantum yield calculation and light on/off experiments:

To investigate the possibility of a chain reaction occurring within this system, we conducted relevant experiments as follows, see Supplementary equation (1)

$$(1) \quad \mathrm{mol\ Fe^{2+}} = \frac{\mathrm{V \times \Delta A}}{\mathrm{l \times \varepsilon}} = \frac{\mathrm{0.00235\ L \times 0.2275339}}{\mathrm{1.000\ cm \times 11100\ mol^{-1}} cm^{-1}} = 4.82 \times\ 10^{-8}\ mol$$

Where V is the volume of the respective sample solution analyzed (2.35 mL), ΔA is the difference between the average absorbance of irradiated and

nonirradiated ferrioxalate solutions at 510 nm, l is the pathlength, and the ϵ is the molar absorptivity at 510 nm. See Supplementary equation (2)

$$(2)\quad \text{photon flux} = \frac{\text{mol Fe}^{2+}}{\varphi \times \text{t} \times \text{f}} = \frac{4.82\times 10^{-8}\ mol}{0.92 \times 10\ \text{s} \times 0.99066} = 5.28 \times 10^{-9}\ einstein\ s^{-1}$$

Where φ is the quantum yield for the ferrioxalate actinometer at 450 nm, t is the irradiation time and f are the fraction of light absorbed by the ferrioxalate actinometer solution. See Supplementary equation (3)

$$(3)\quad f = 1 - 10^{-A} = 1 - 10^{-2.02} = 0.99$$

Where A is the measured absorbance of the ferrioxalate actinometer solution at 456 nm before blue LED irradiation without 1,10-phenanthroline.

Determination of the fraction of light absorbed at 450 nm by the ferrioxalate solution: The absorbance of the ferrioxalate actinometer solution at 450 nm before blue LED irradiation, without 1,10-phenanthroline, was measured at 2.029595.

The quantum yield (Φ) has been calculated using the Supplementary equation (4):

$$(4)\quad \Phi = \frac{\text{mol Product}}{\text{f} \times \text{t} \times \text{flux}} = \frac{0.5 \times 0.09 \times 10^{-3}\ mol}{0.99 \times 3600\ \text{s} \times 5.28 \times 10^{-9}\ einstein\ s^{-1}} = 2.39$$

Where t is the reaction time, f is previously calculated fraction of light absorbed by the respective solution.

The results of the light on/off experiments are as follows:

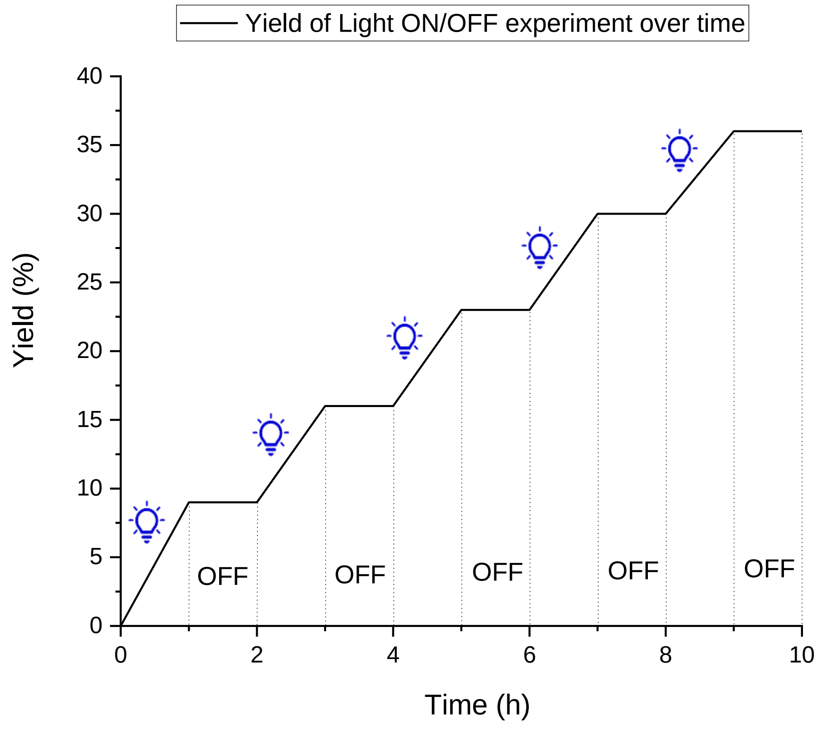

Supplementary Figure 28. light on/off experiments

Annotation: For the accuracy and reliability of the experimental results, all experimental data were repeated three times and the average values were taken.

Combining the experimental results and related literature[22,23], we can conclude that the reaction is likely a light-driven chain reaction. The illumination provides the necessary energy for initiating and maintain the chain reaction. The experimental findings, coupled with studies on the mechanism in related literature[24,25], hint at the existence of short chain reactions. Specifically, this involves the formation of oxidizing peroxide or radical intermediates through the reaction of certain amine radical intermediates with oxygen.

(F) ^{1}H NMR, ^{13}C NMR Spectra of Products:

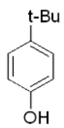

2a

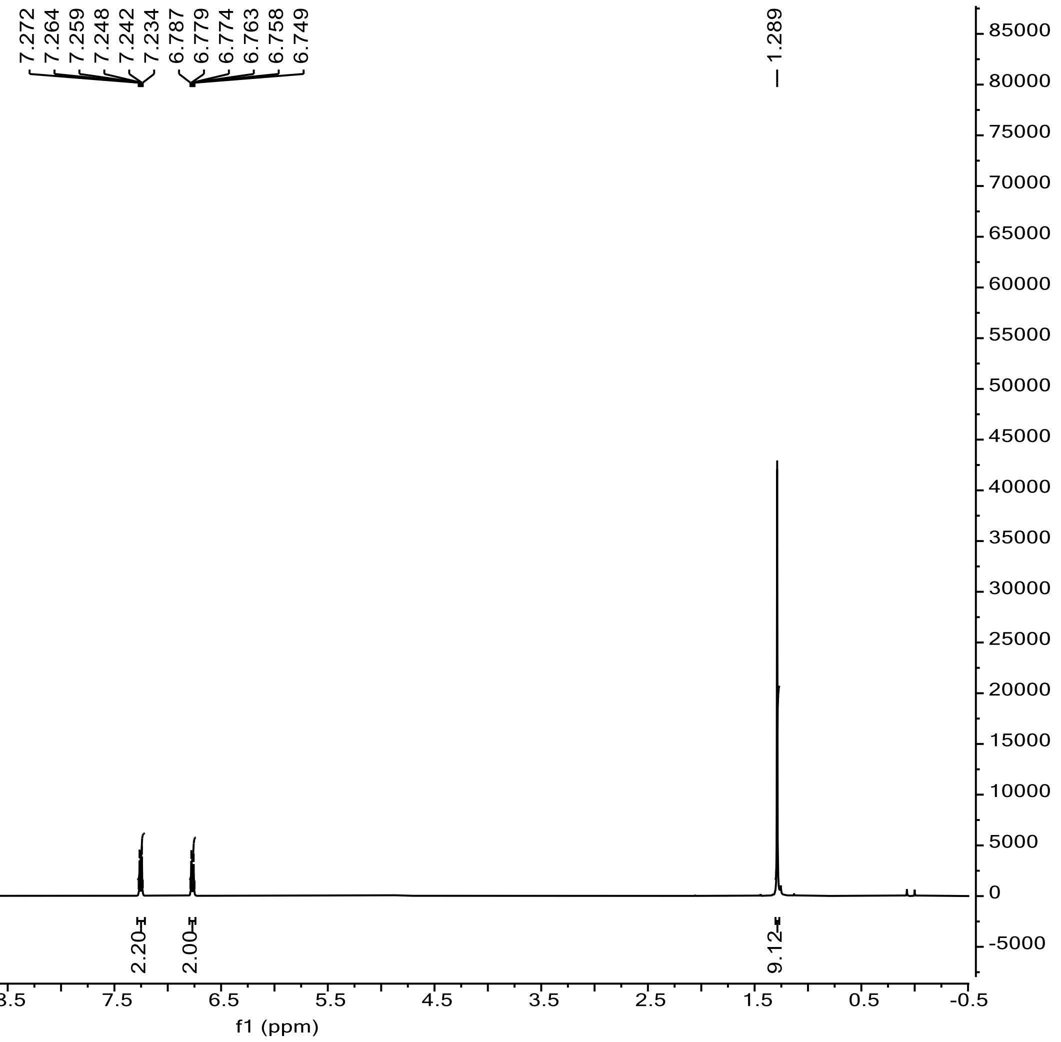

Supplementary Figure 29. ^{1}H NMR Spectrum for 2a

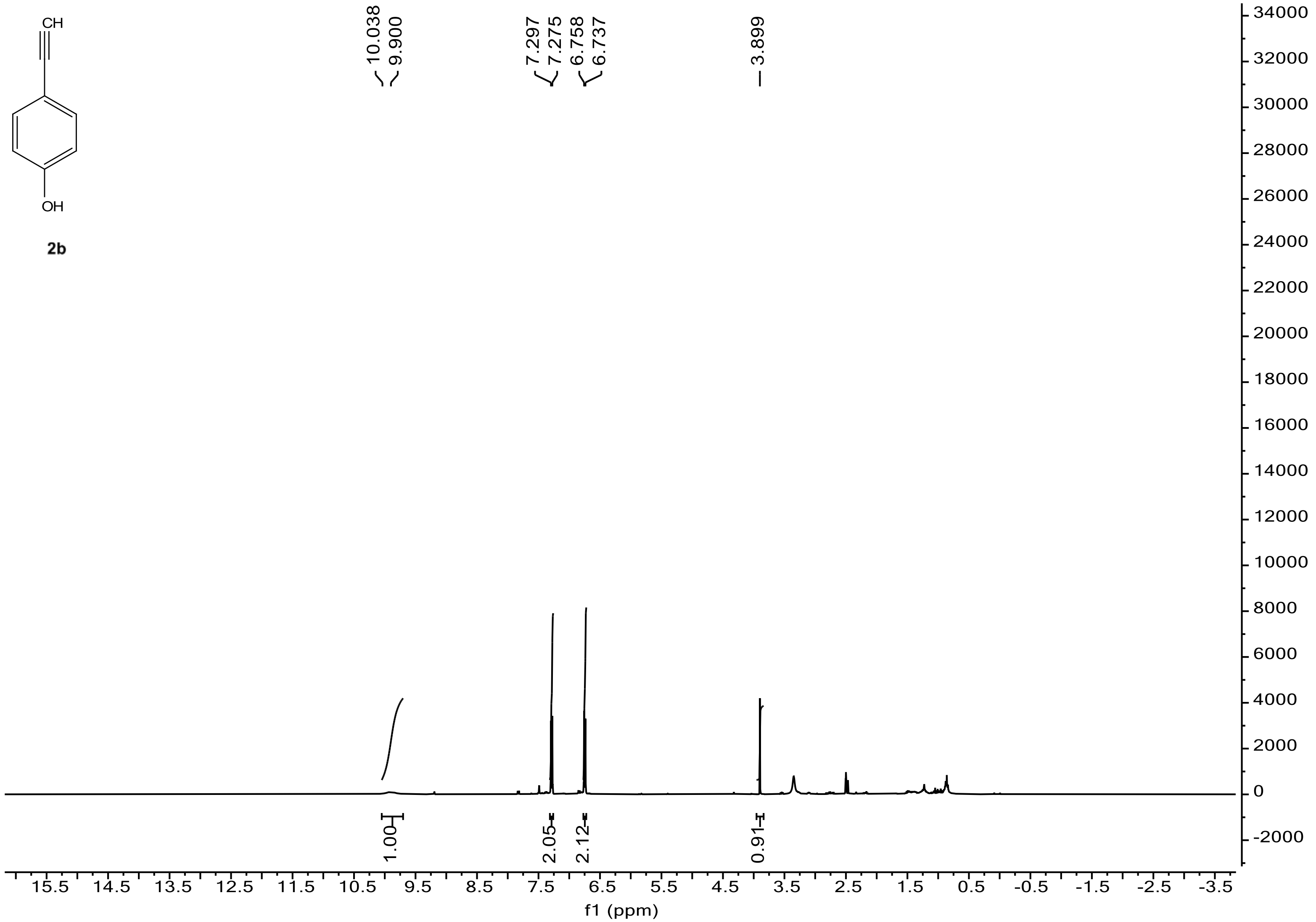

Supplementary Figure 30. ^{1}H NMR Spectrum for 2b

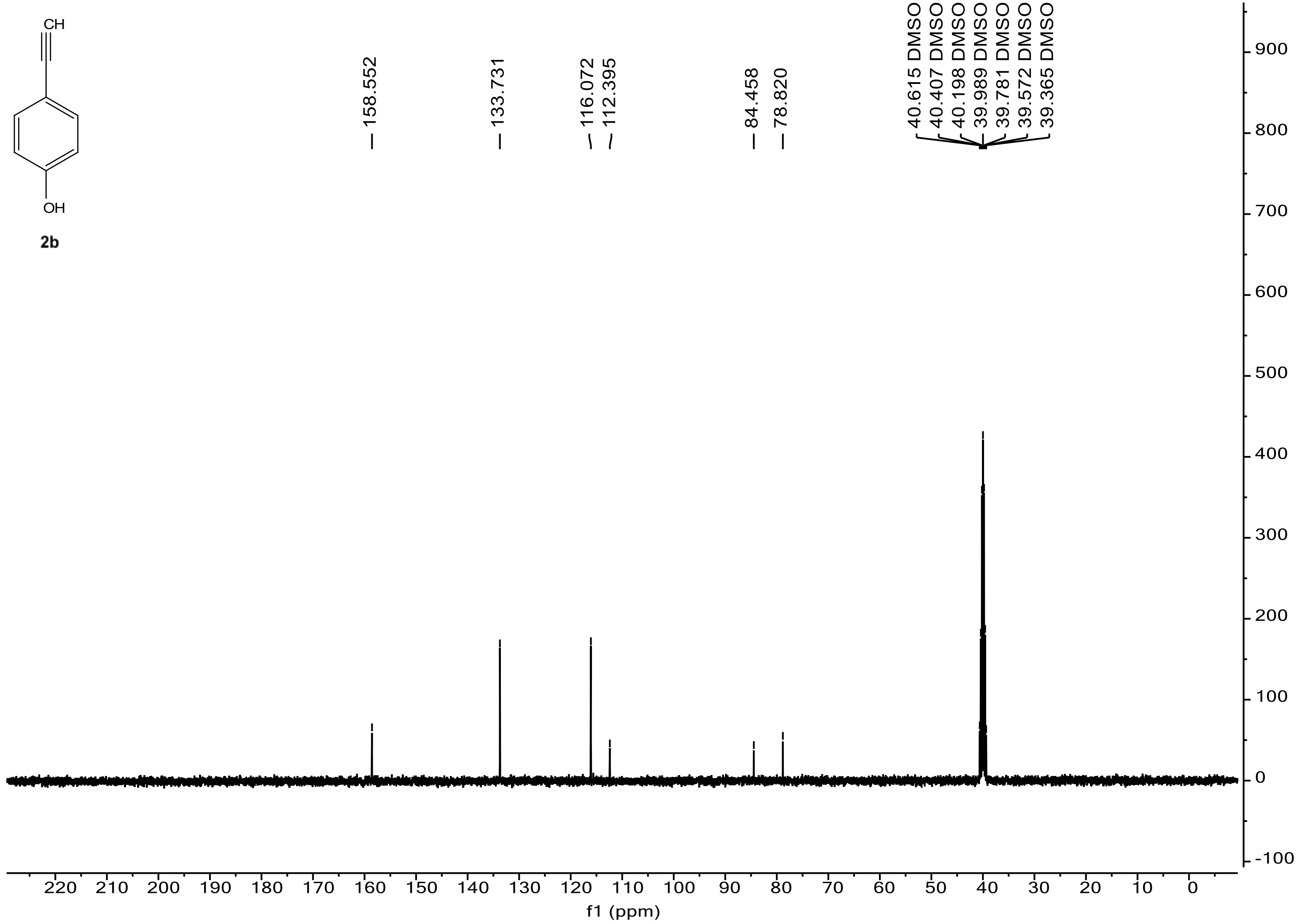

Supplementary Figure 31. ^{13}C NMR Spectrum for 2b

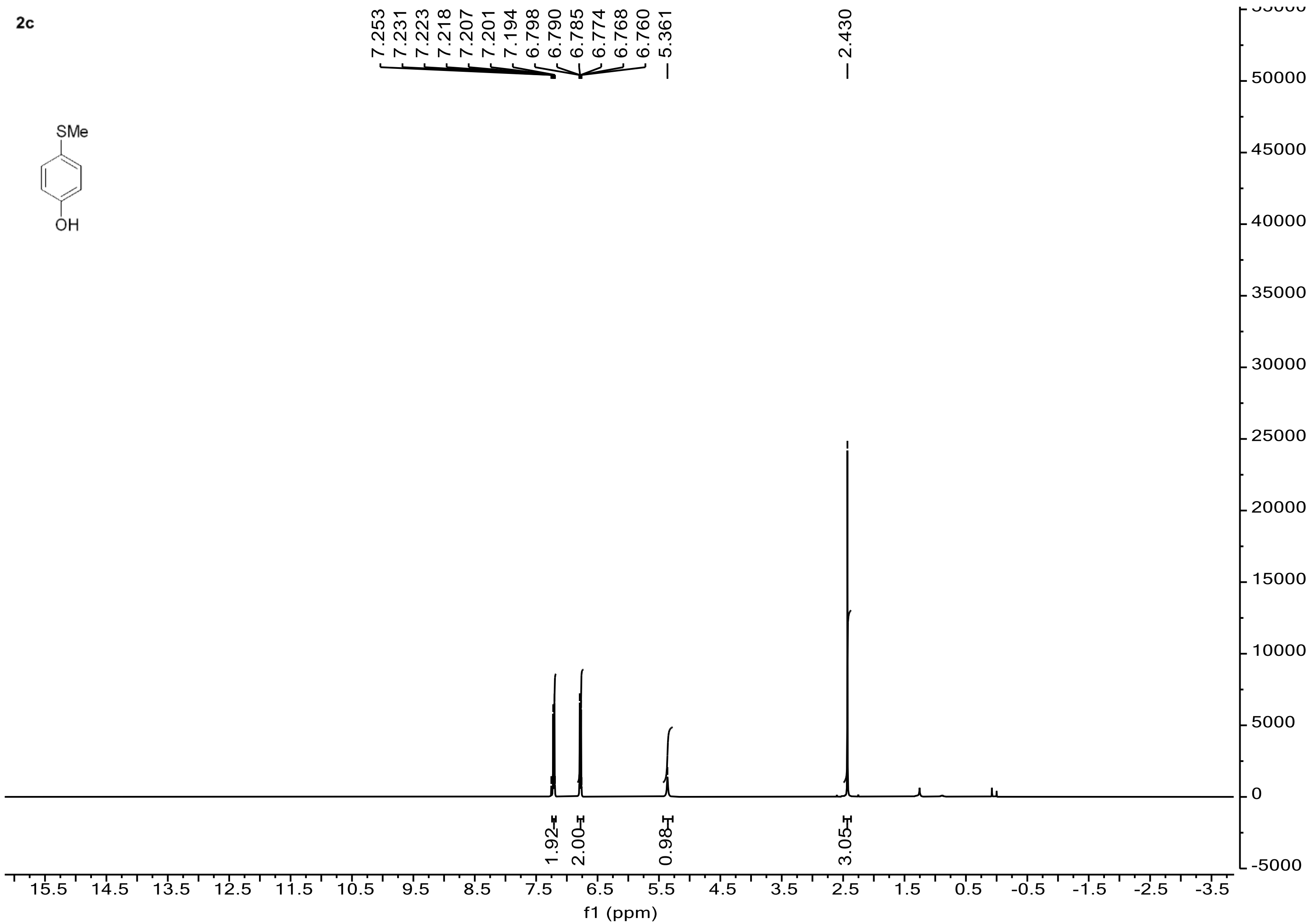

Supplementary Figure 32. ^{1}H NMR Spectrum for 2c

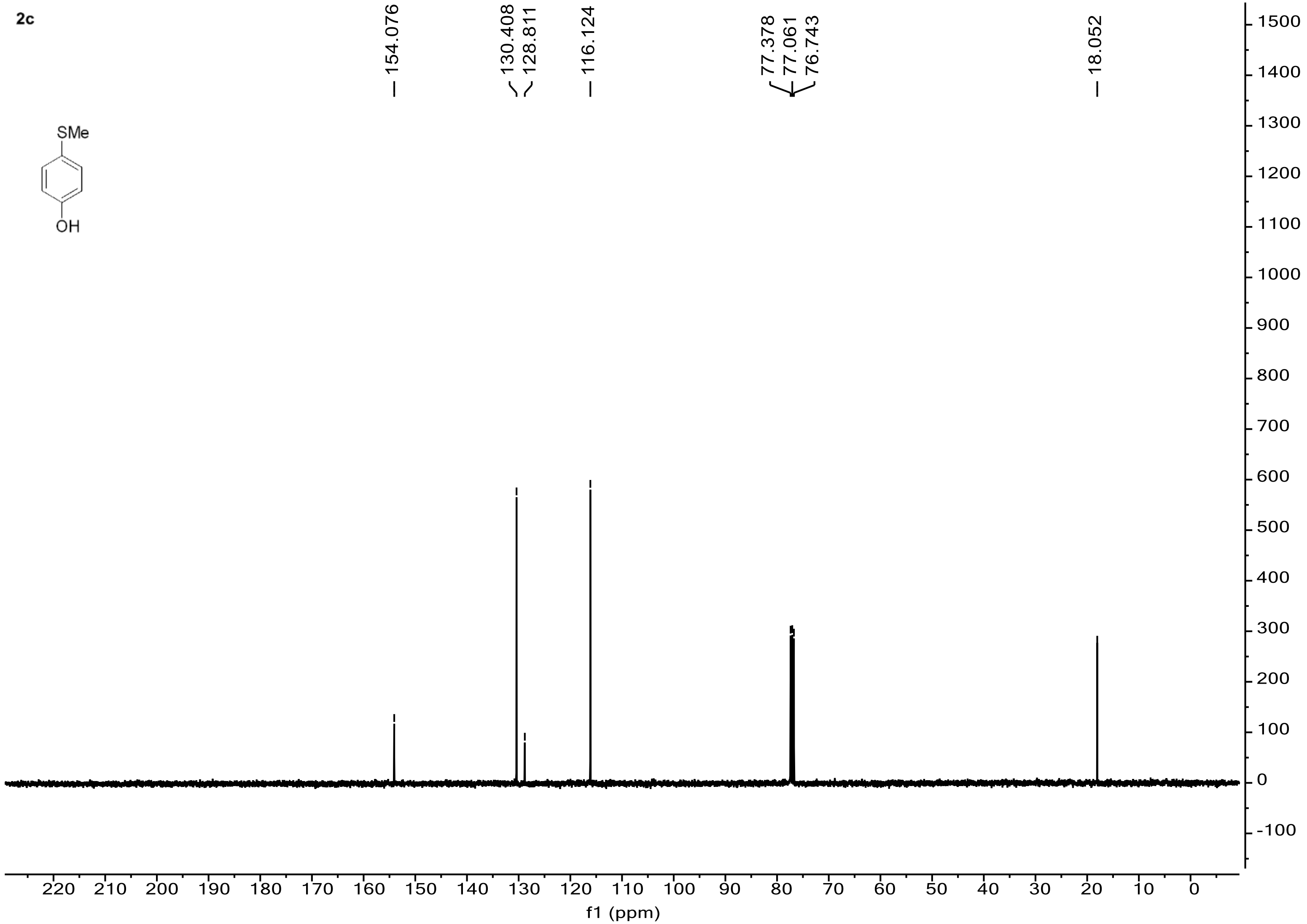

Supplementary Figure 33. ^{13}C NMR Spectrum for 2c

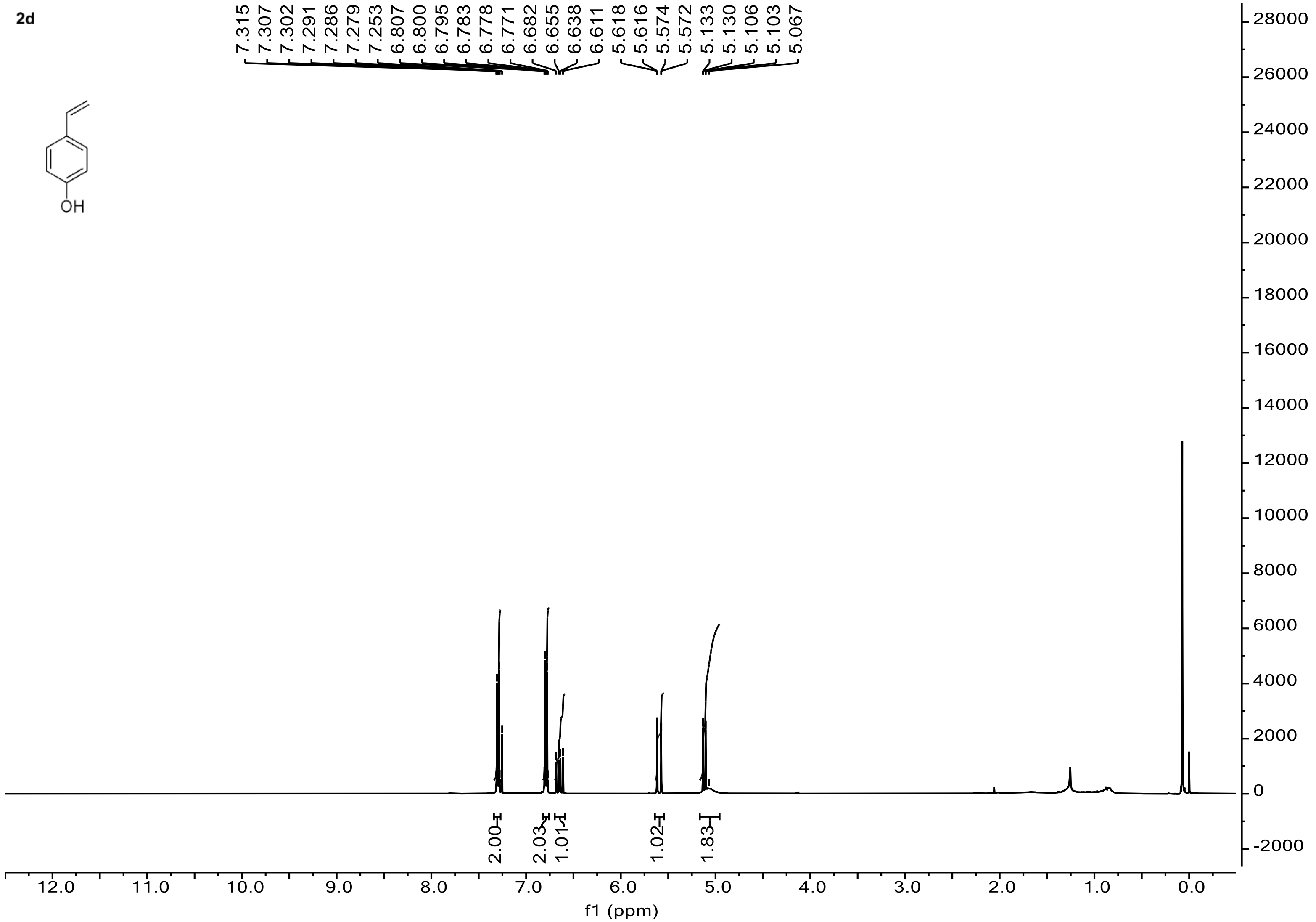

Supplementary Figure 34. ^{1}H NMR Spectrum for 2d

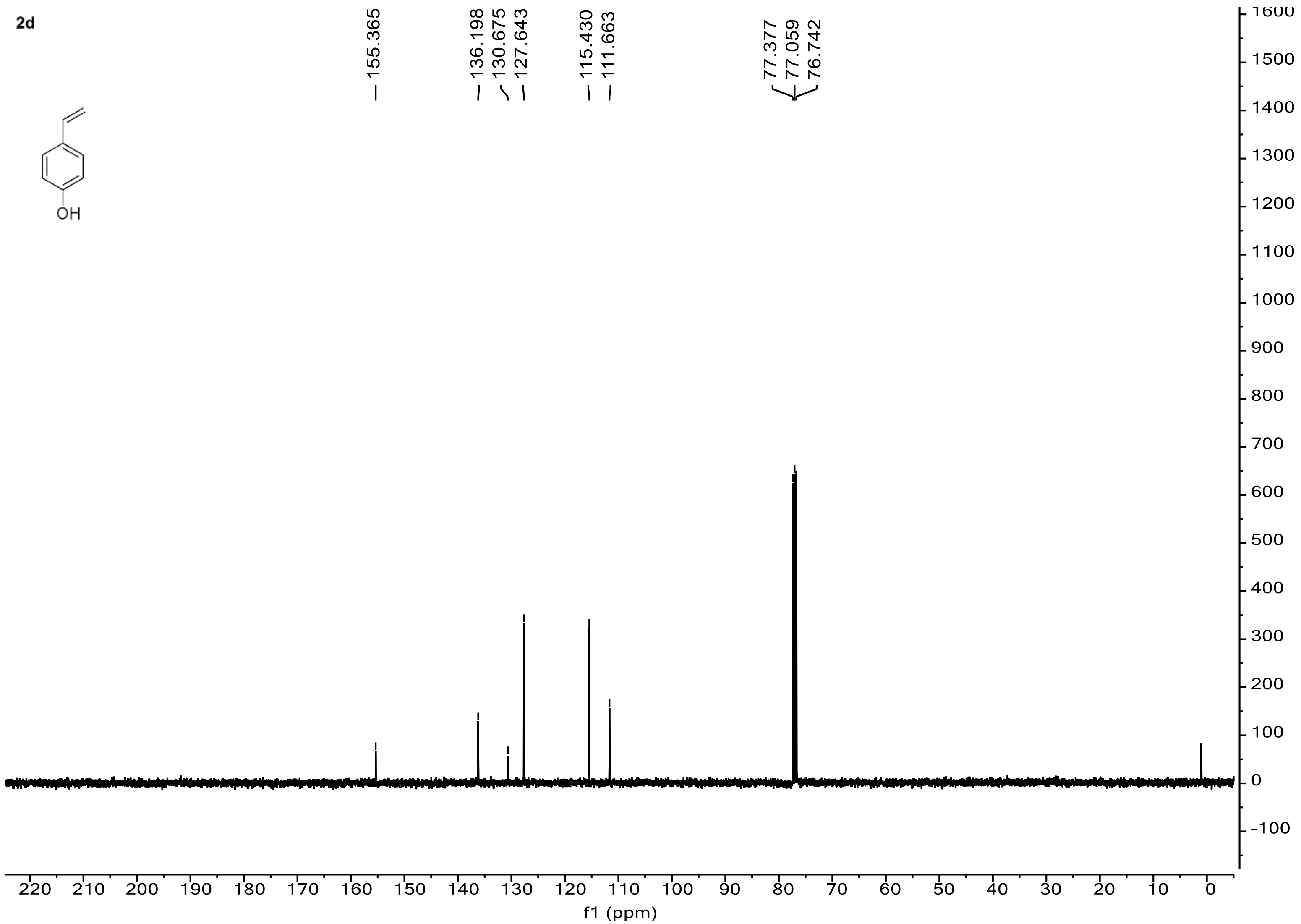

Supplementary Figure 35. ^{13}C NMR Spectrum for 2d

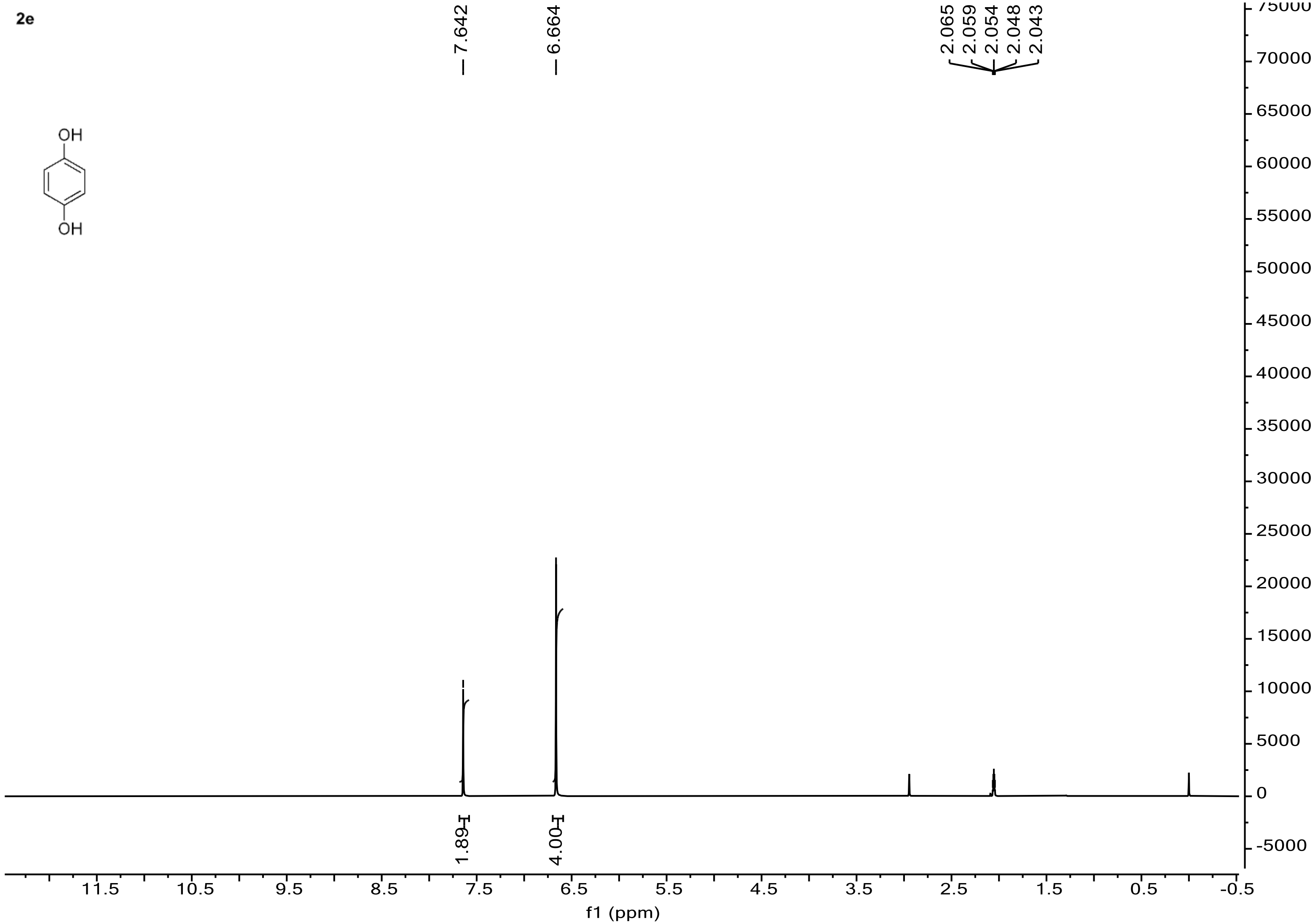

Supplementary Figure 36. ^{1}H NMR Spectrum for 2e

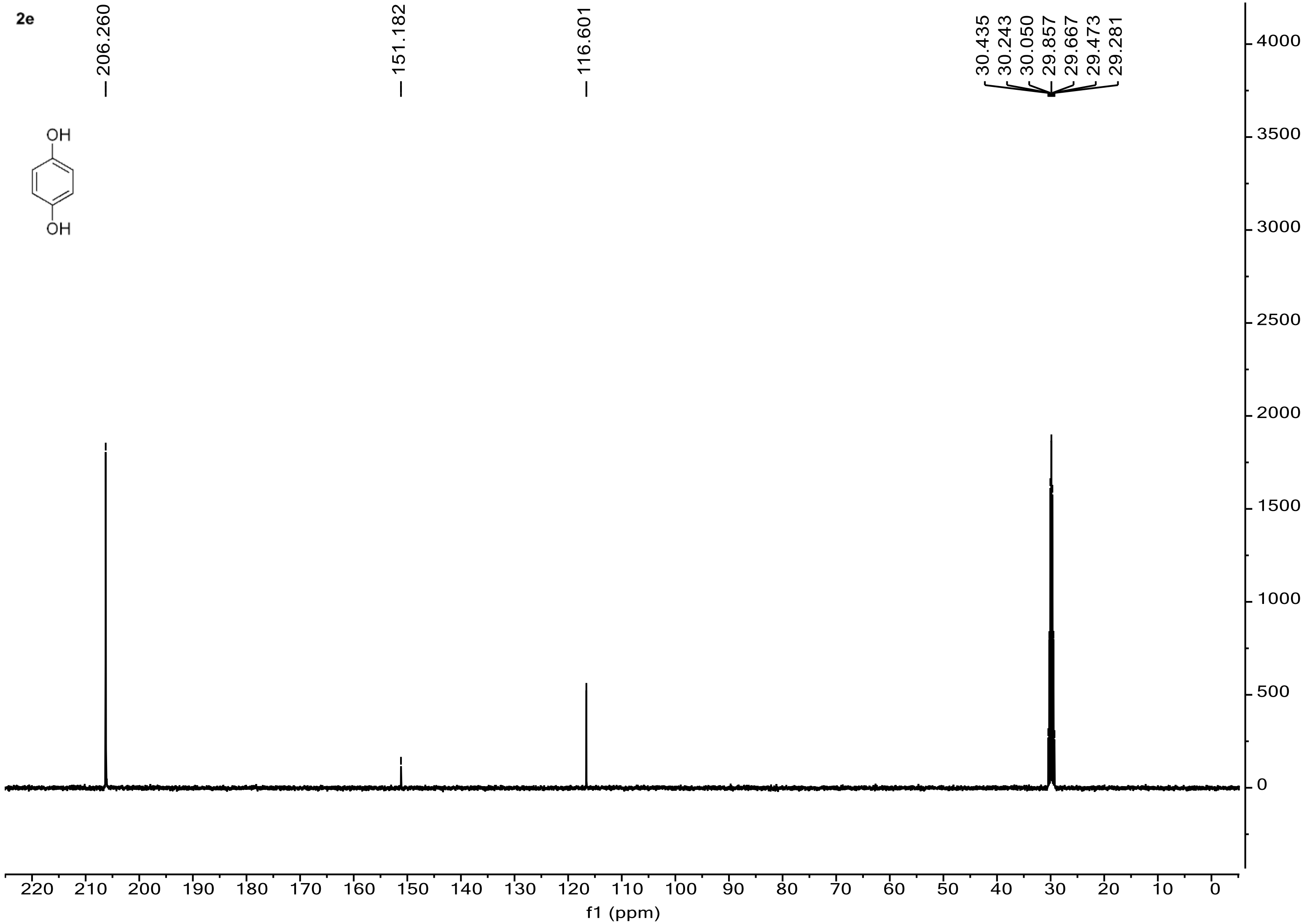

Supplementary Figure 37. ^{13}C NMR Spectrum for 2e

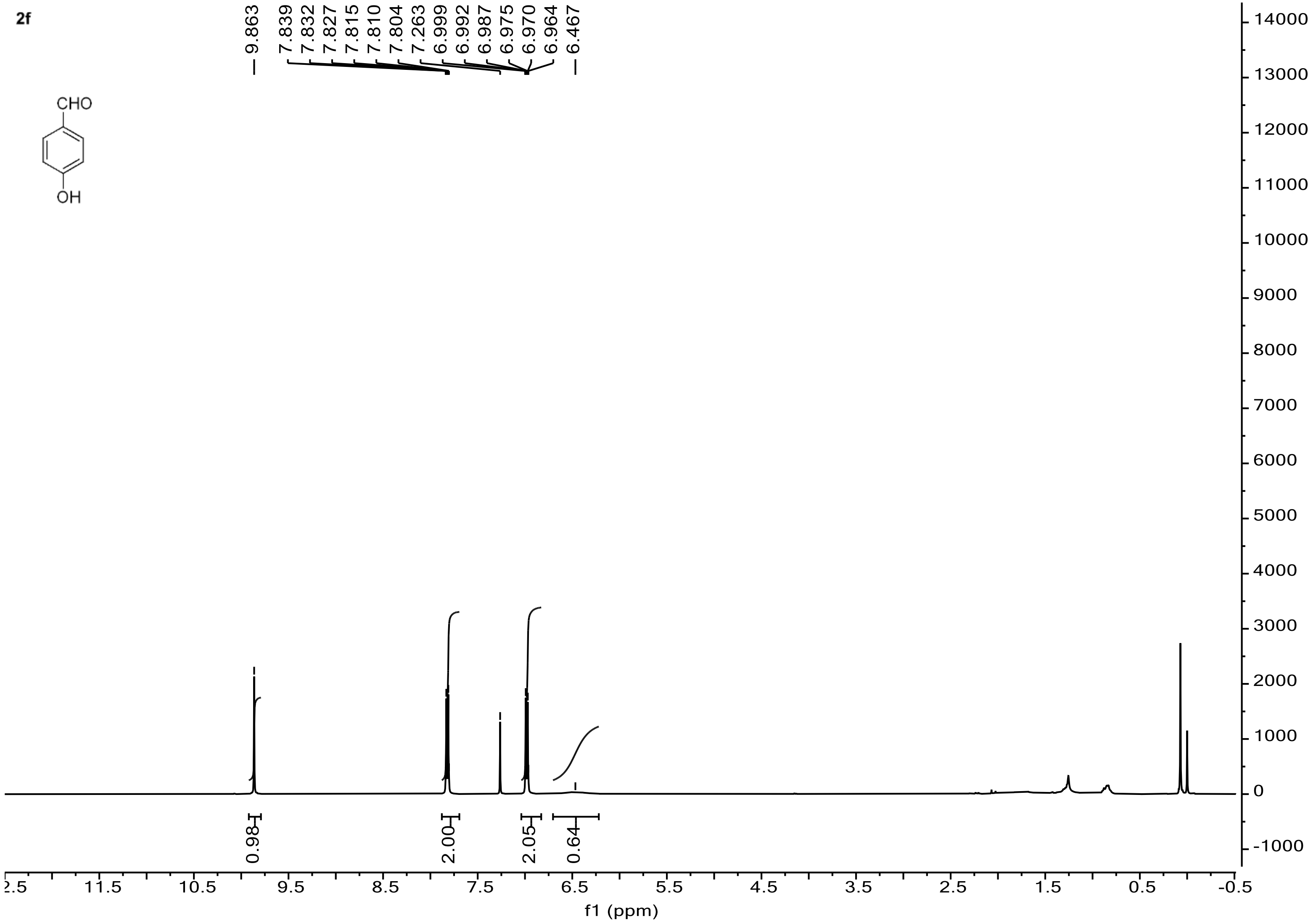

Supplementary Figure 38. ^{1}H NMR Spectrum for 2f

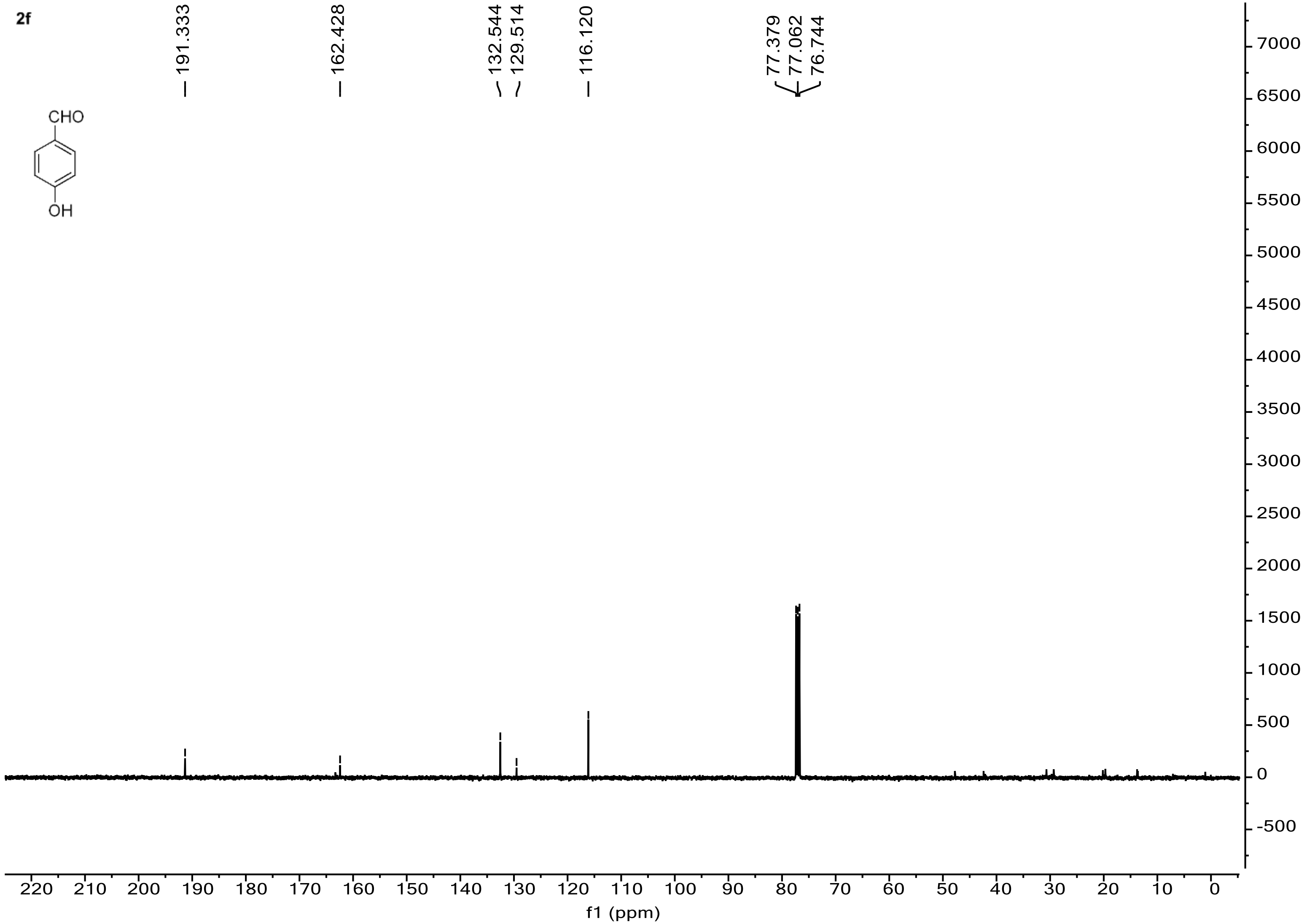

Supplementary Figure 39. ^{13}C NMR Spectrum for 2f

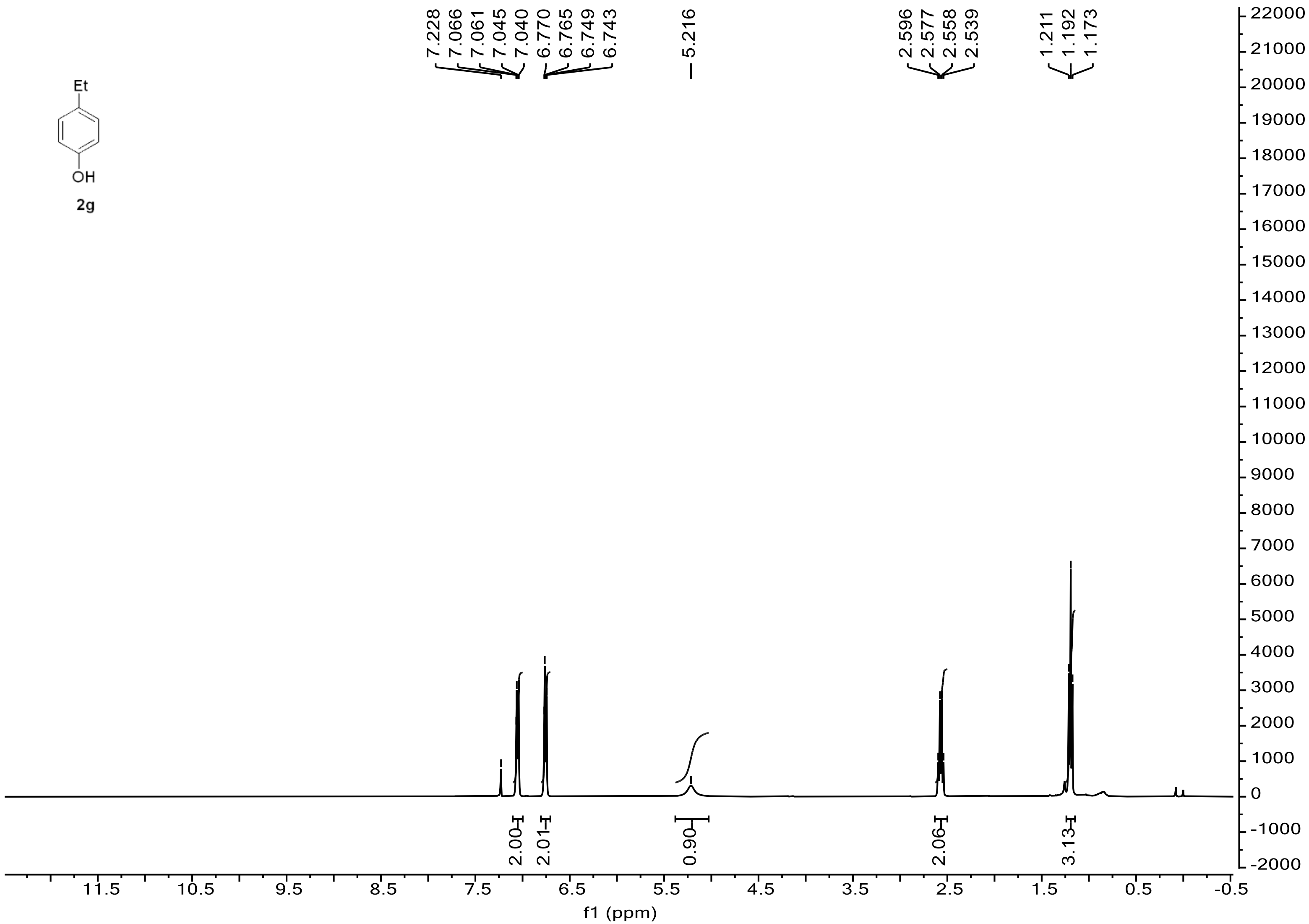

Supplementary Figure 40. ^{1}H NMR Spectrum for 2g

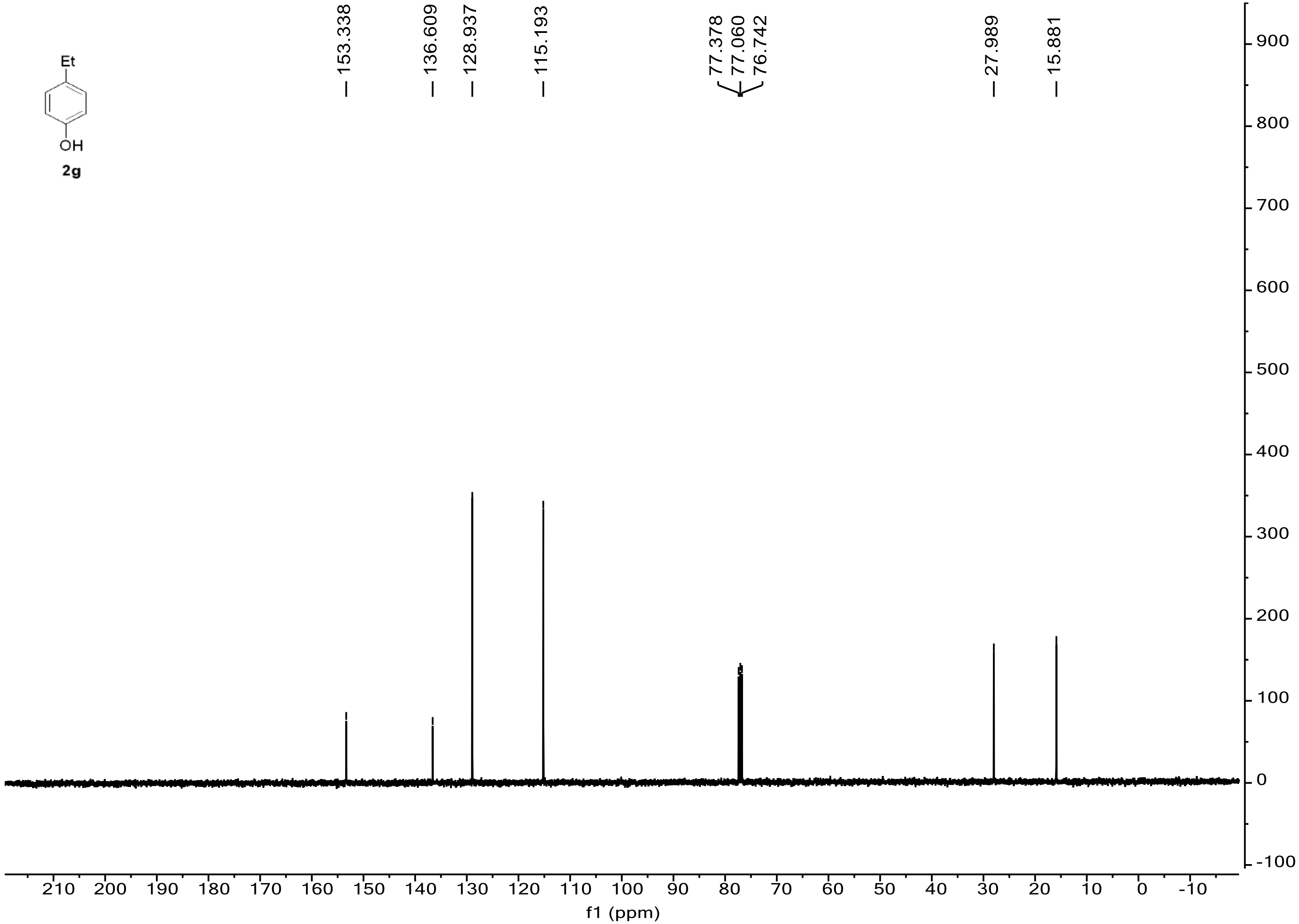

Supplementary Figure 41. ^{13}C NMR Spectrum for 2g

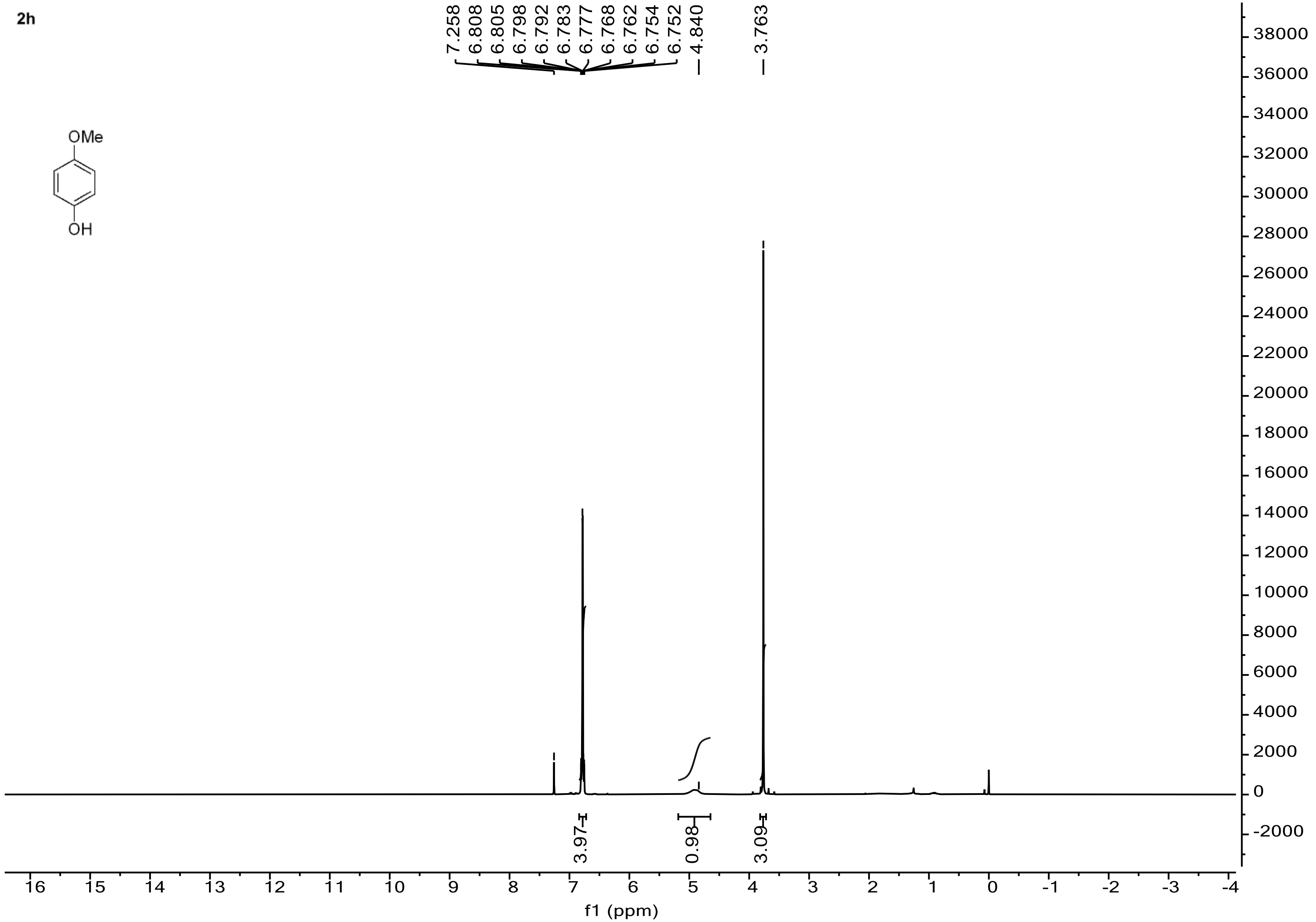

Supplementary Figure 42. ^{1}H NMR Spectrum for 2h

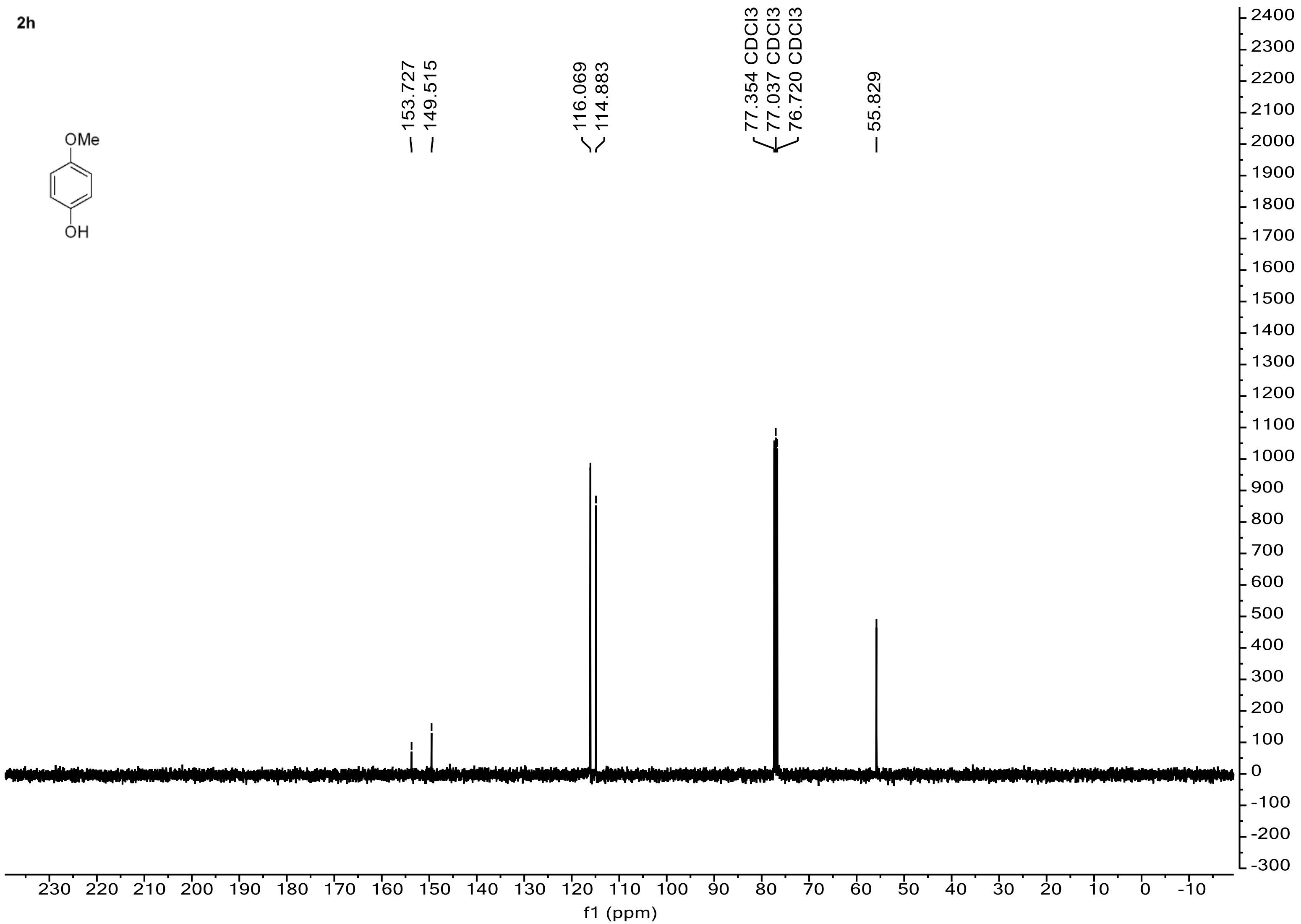

Supplementary Figure 43. ^{13}C NMR Spectrum for 2h

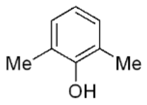

2i

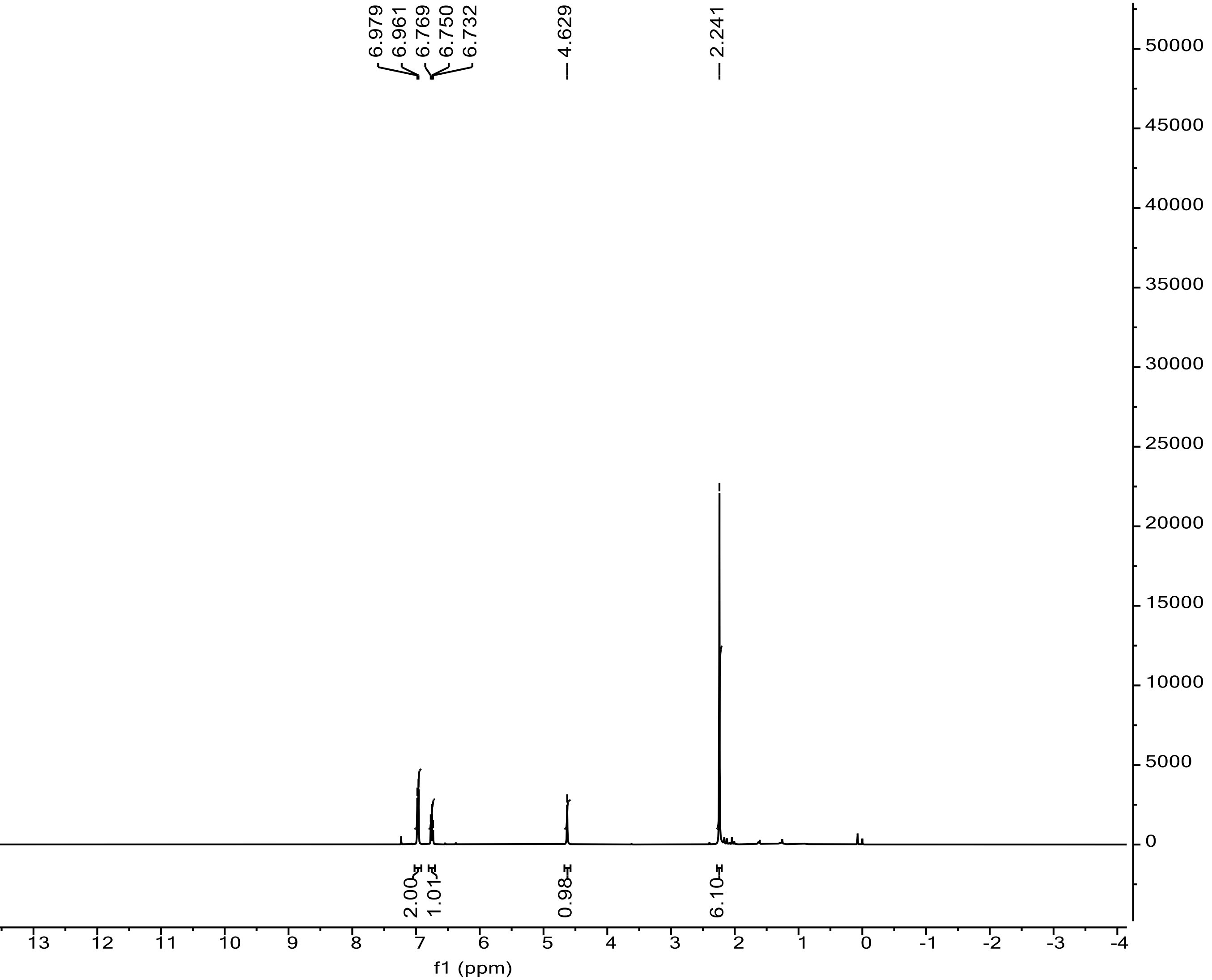

Supplementary Figure 44. ^{1}H NMR Spectrum for 2i

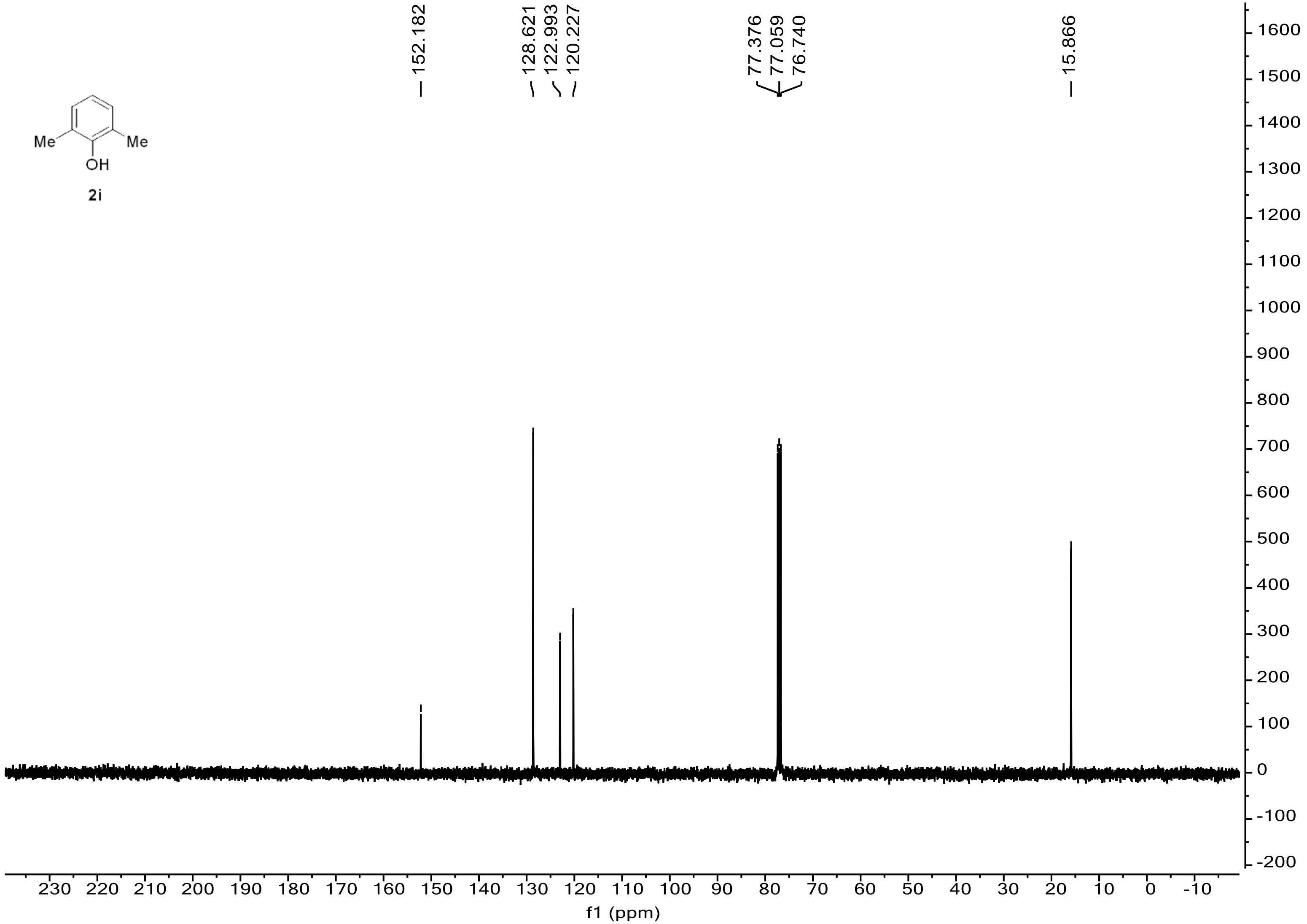

Supplementary Figure 45. ^{13}C NMR Spectrum for 2i

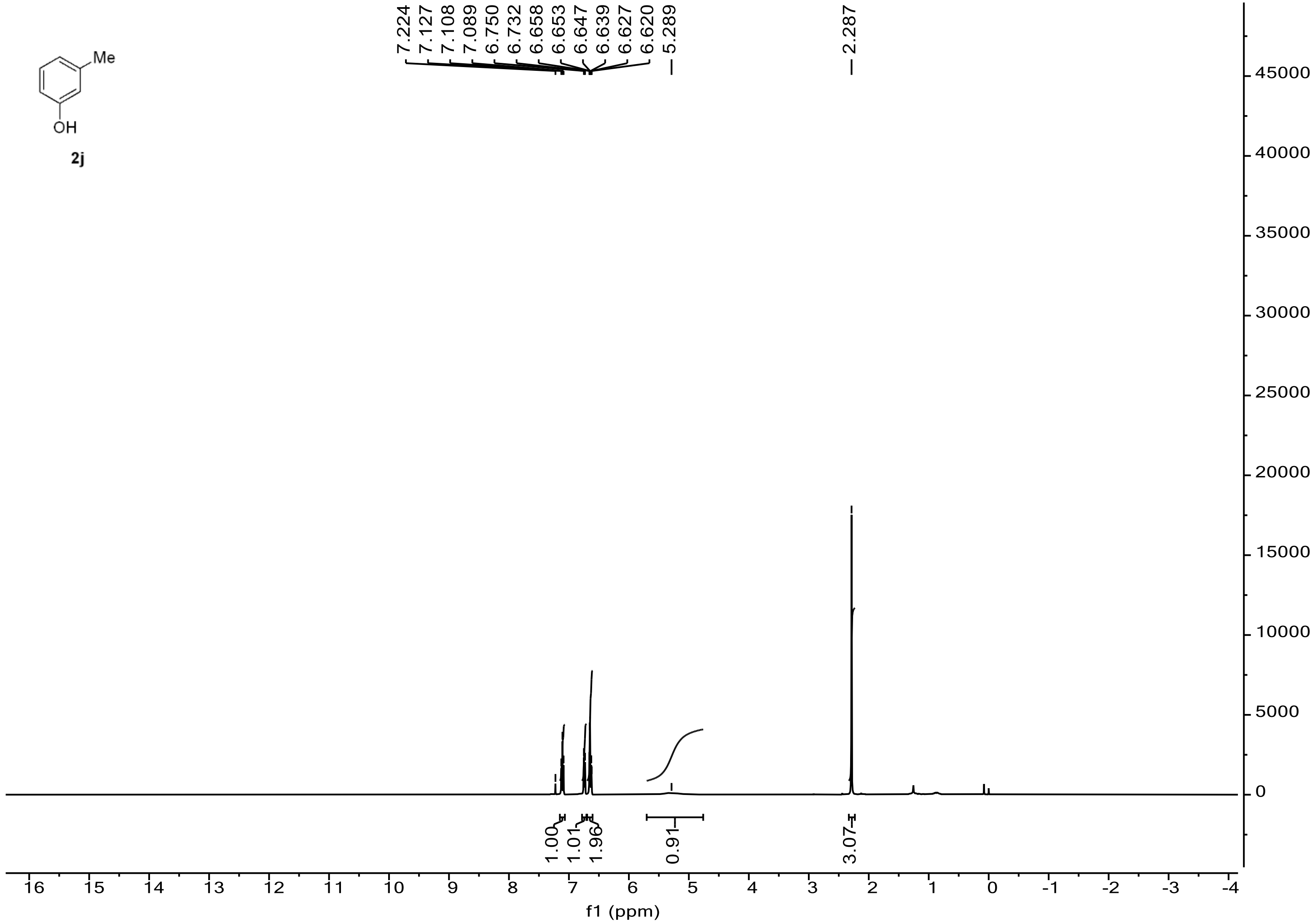

Supplementary Figure 46. ^{1}H NMR Spectrum for 2j

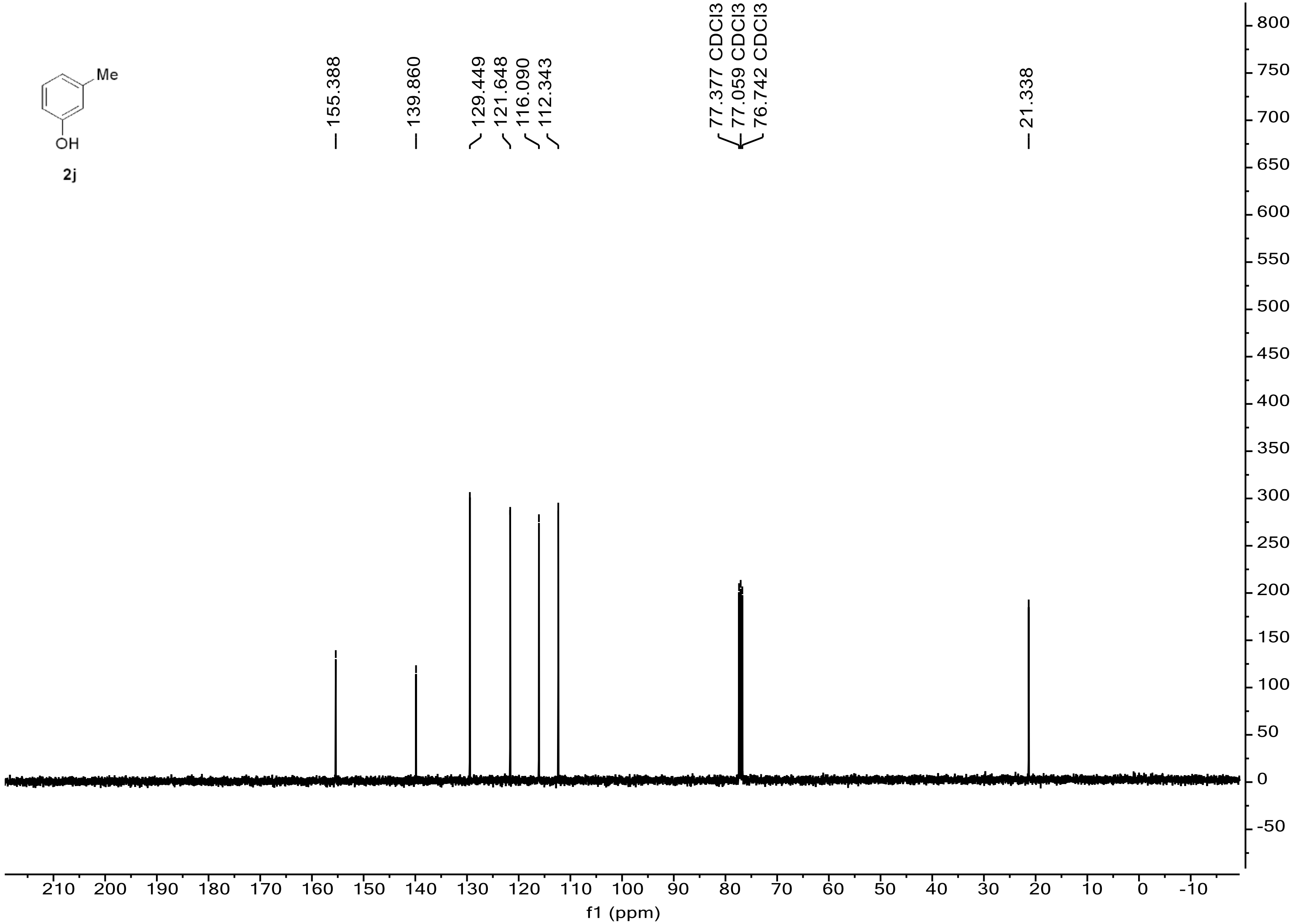

Supplementary Figure 47. ^{13}C NMR Spectrum for 2j

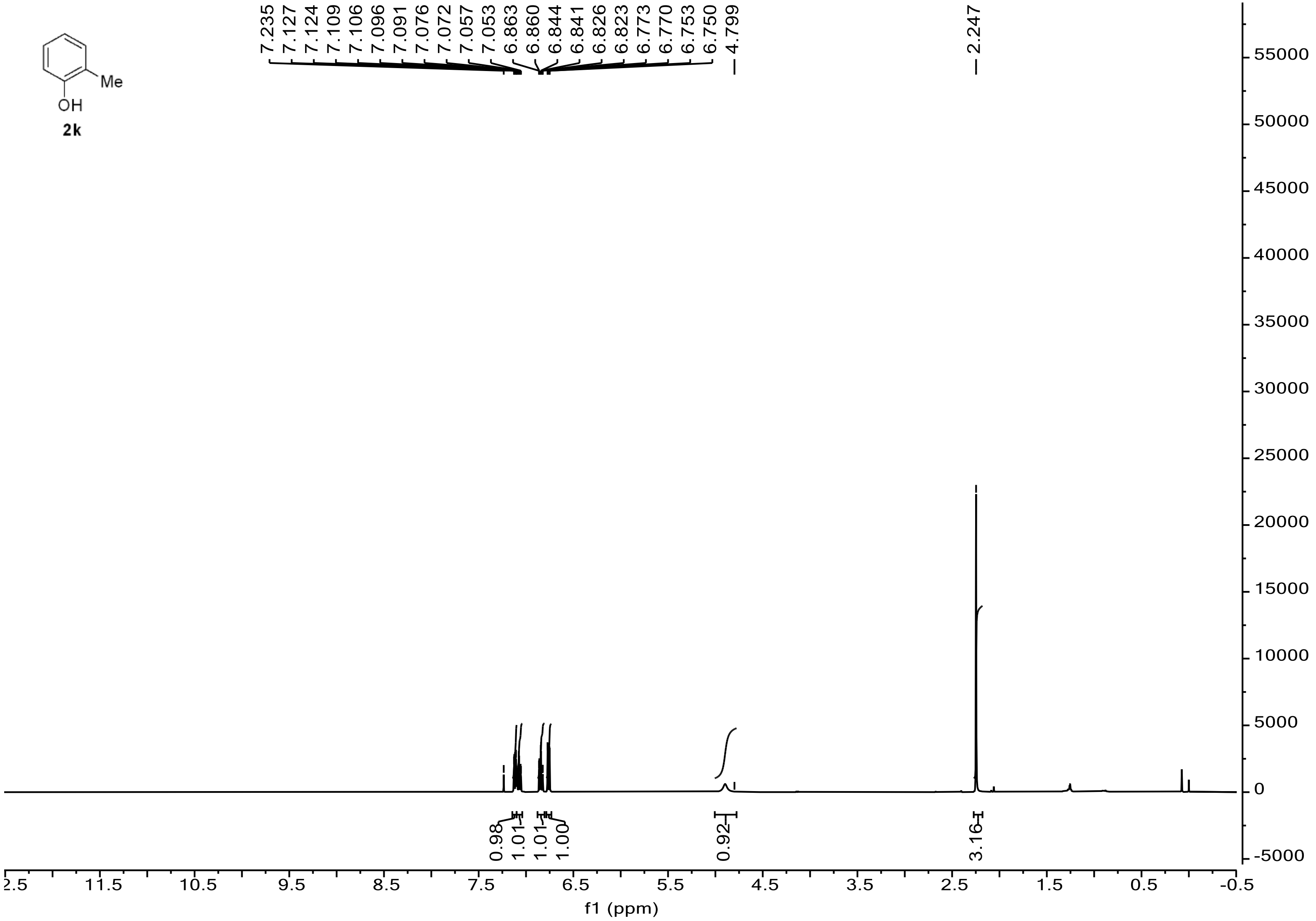

Supplementary Figure 48. ^{1}H NMR Spectrum for 2k

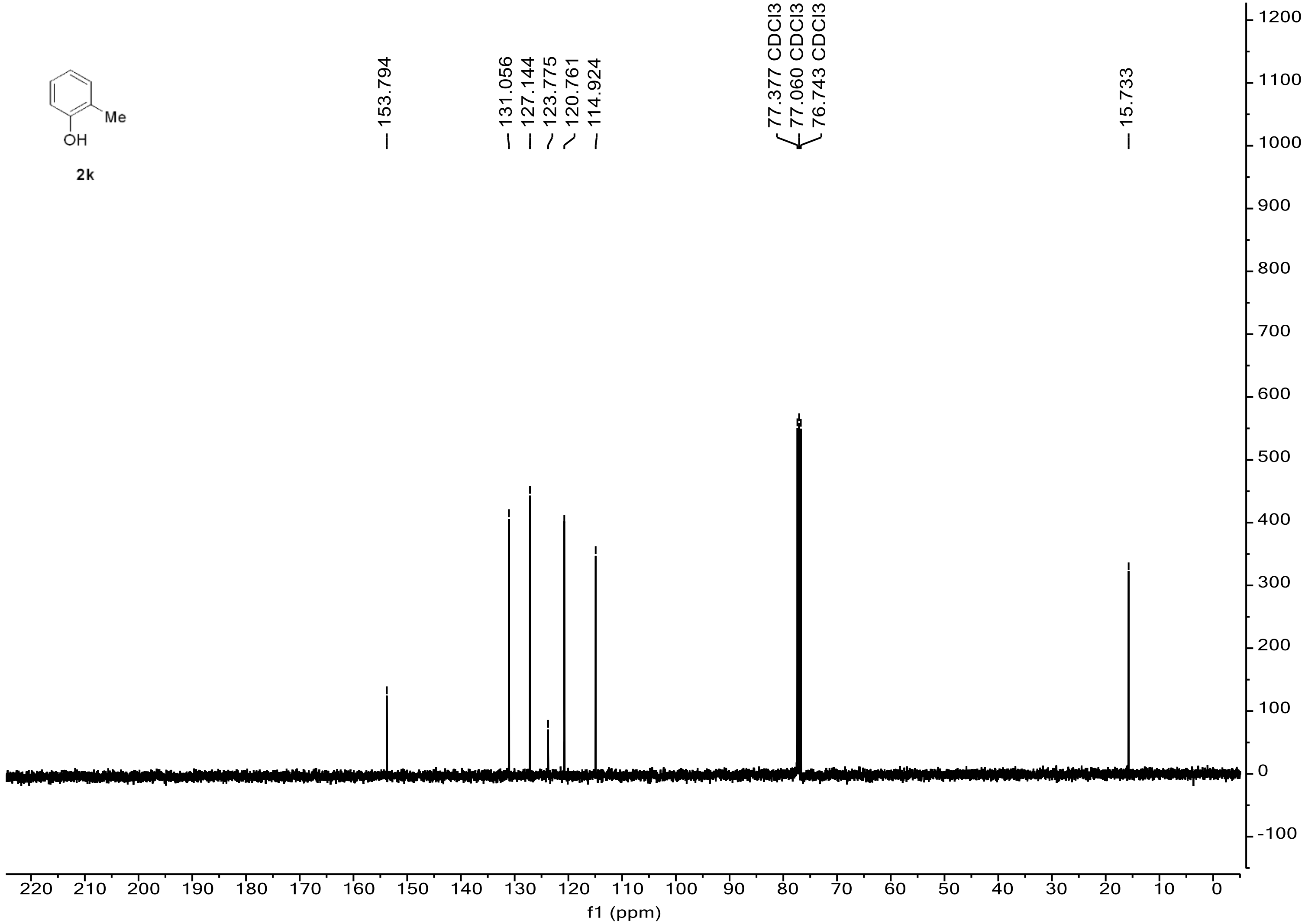

Supplementary Figure 49. ^{13}C NMR Spectrum for 2k

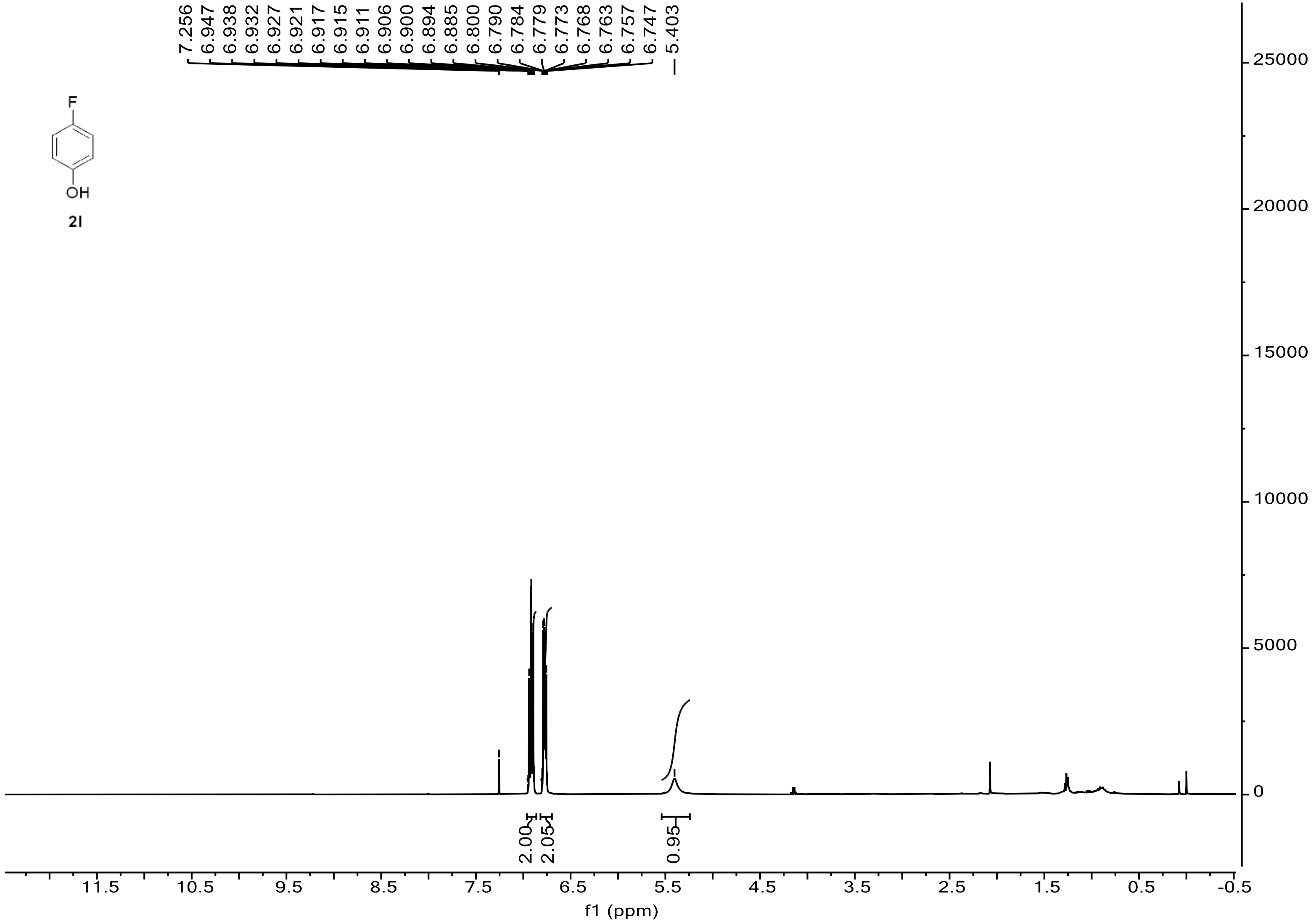

Supplementary Figure 50. ^{1}H NMR Spectrum for 2l

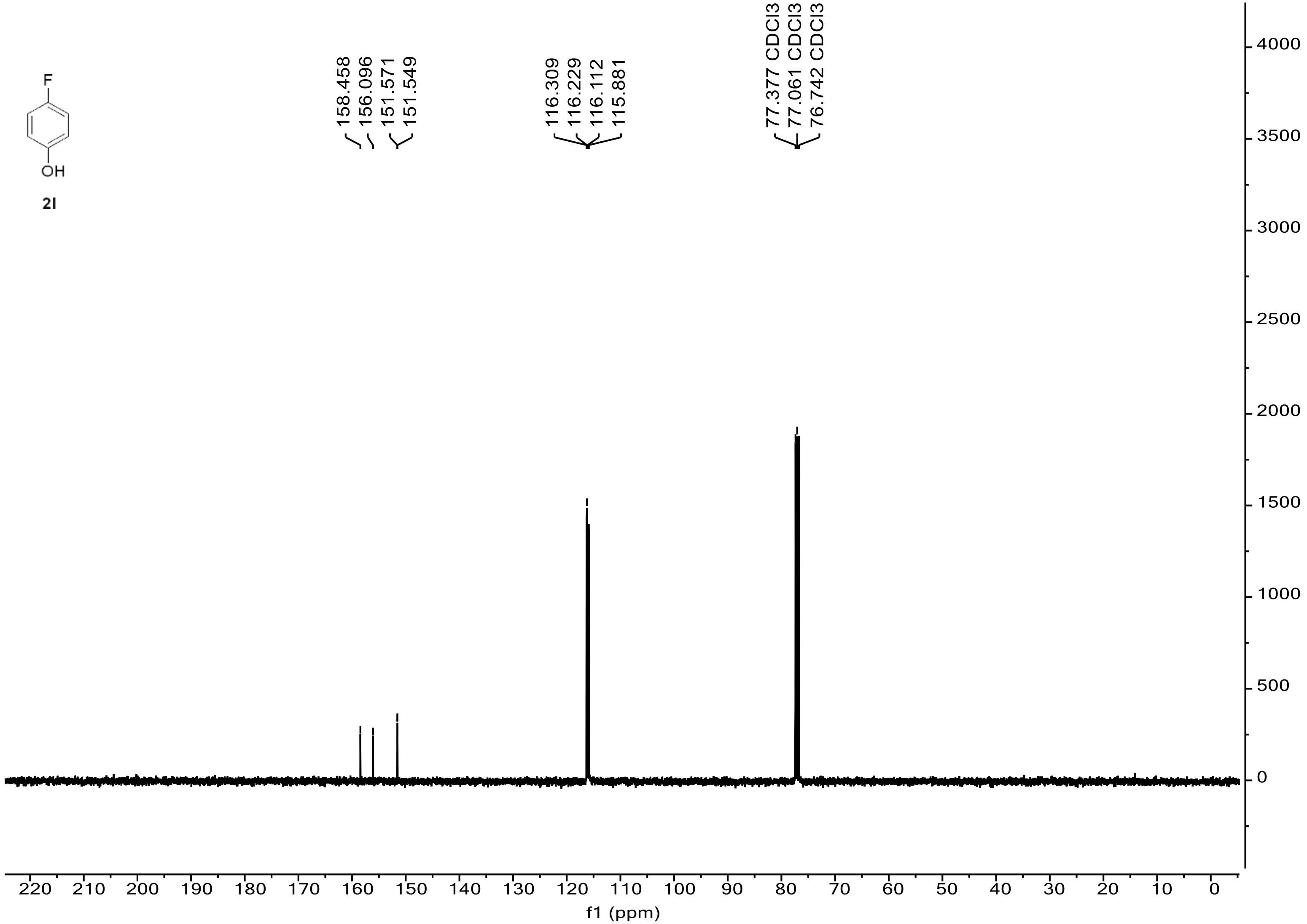

Supplementary Figure 51. ^{13}C NMR Spectrum for 2l

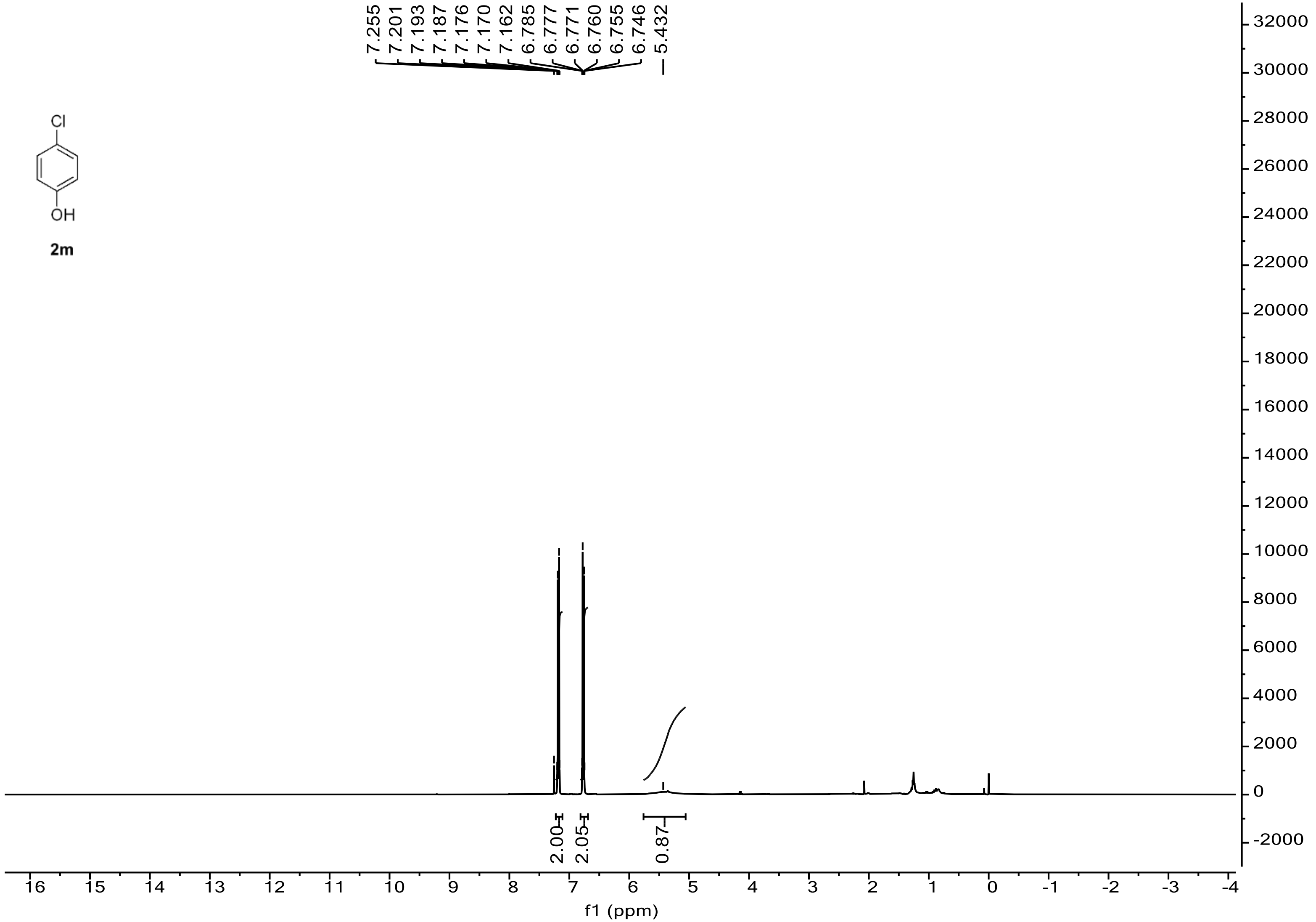

Supplementary Figure 52. ^{1}H NMR Spectrum for 2m

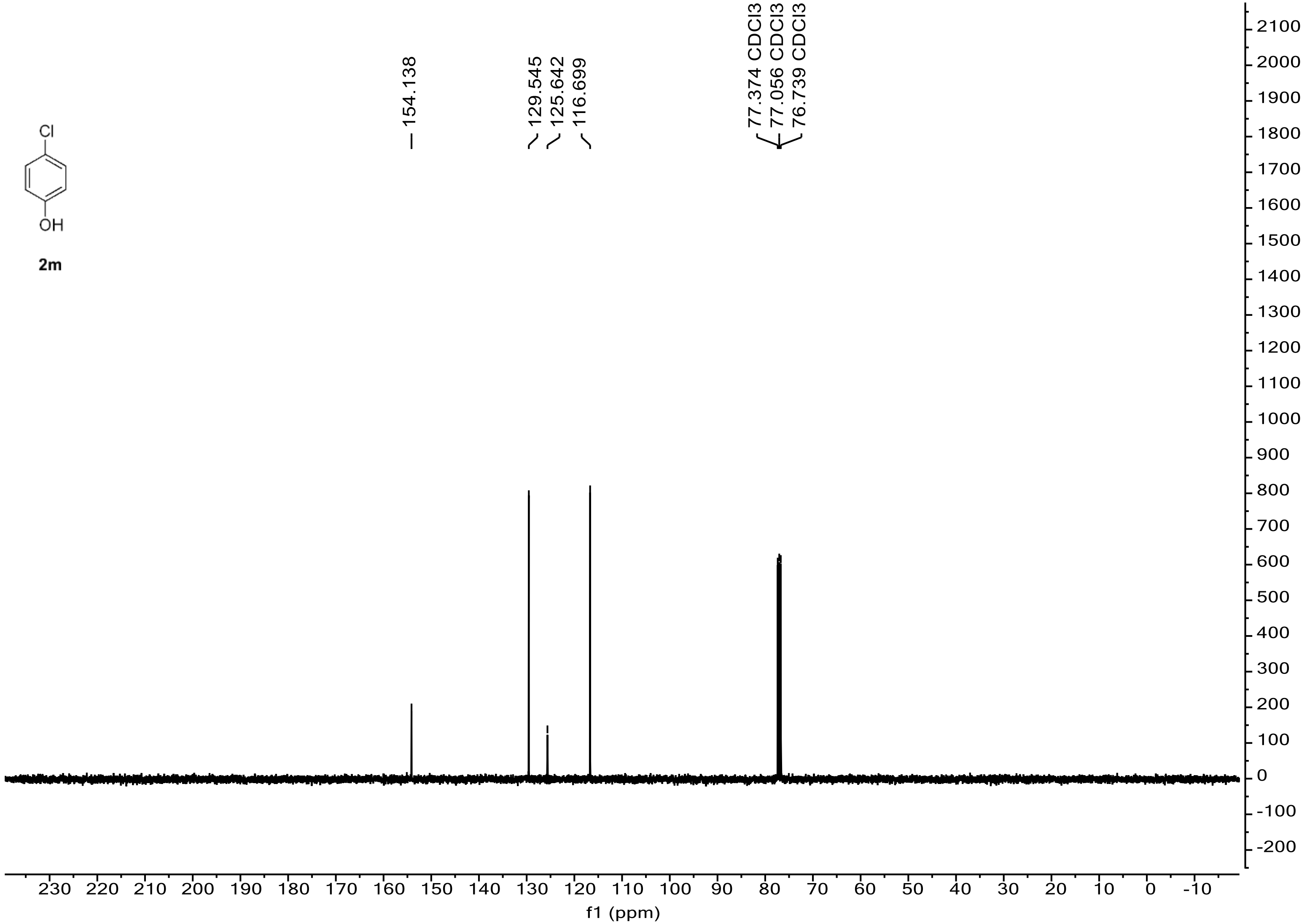

Supplementary Figure 53. ^{13}C NMR Spectrum for 2m

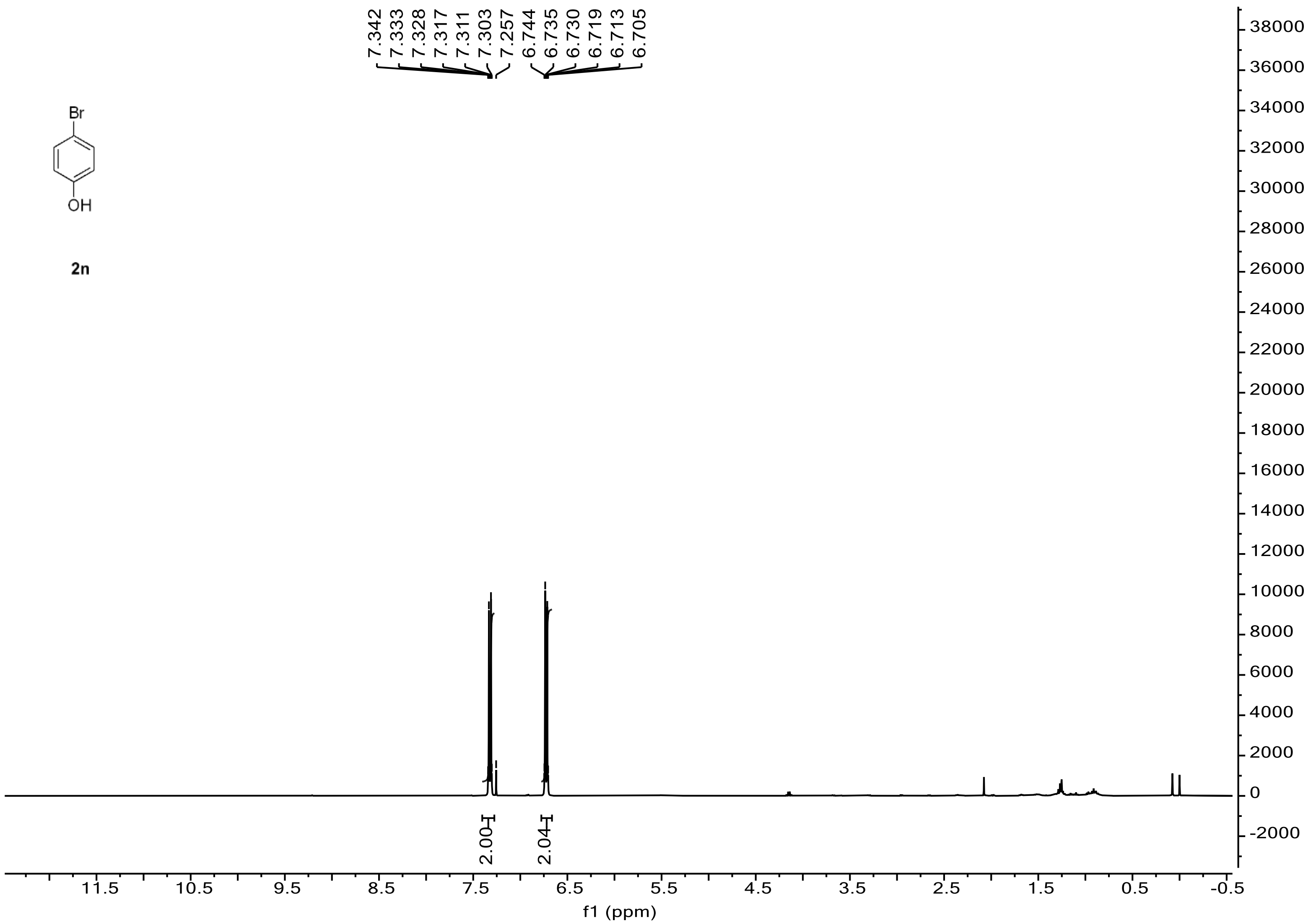

Supplementary Figure 54. ^{1}H NMR Spectrum for 2n

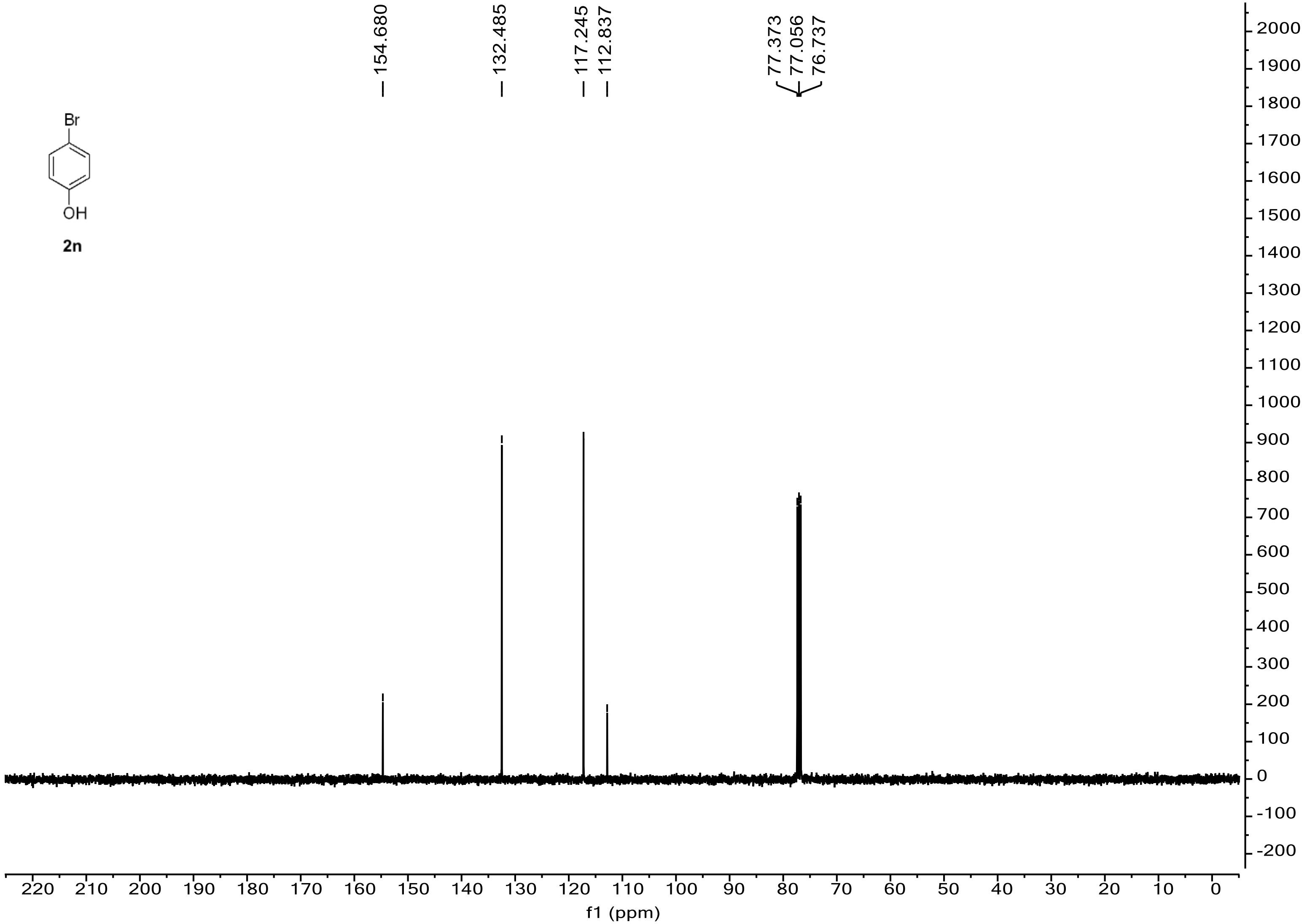

Supplementary Figure 55. ^{13}C NMR Spectrum for 2n

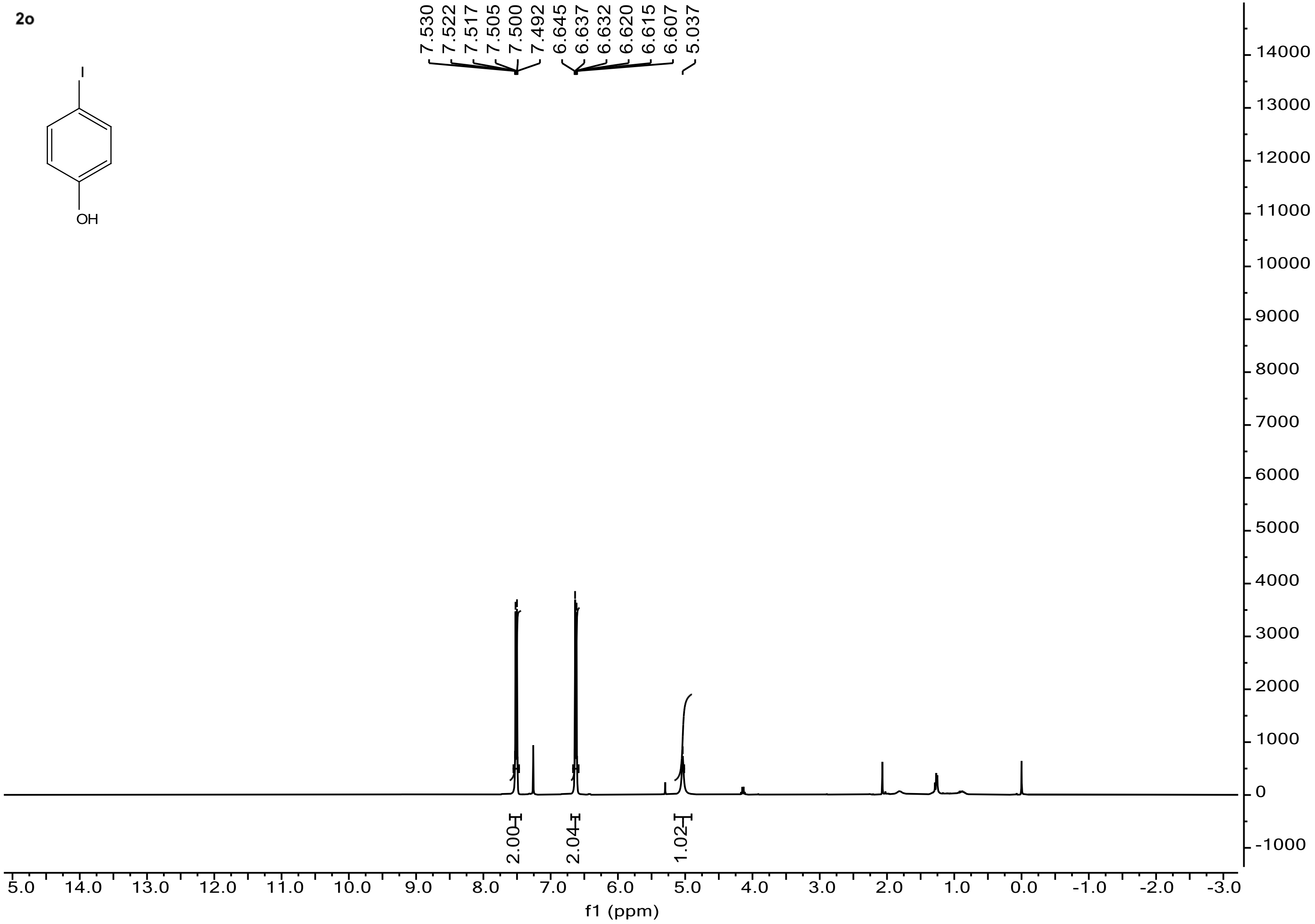

Supplementary Figure 56. ^{1}H NMR Spectrum for 2o

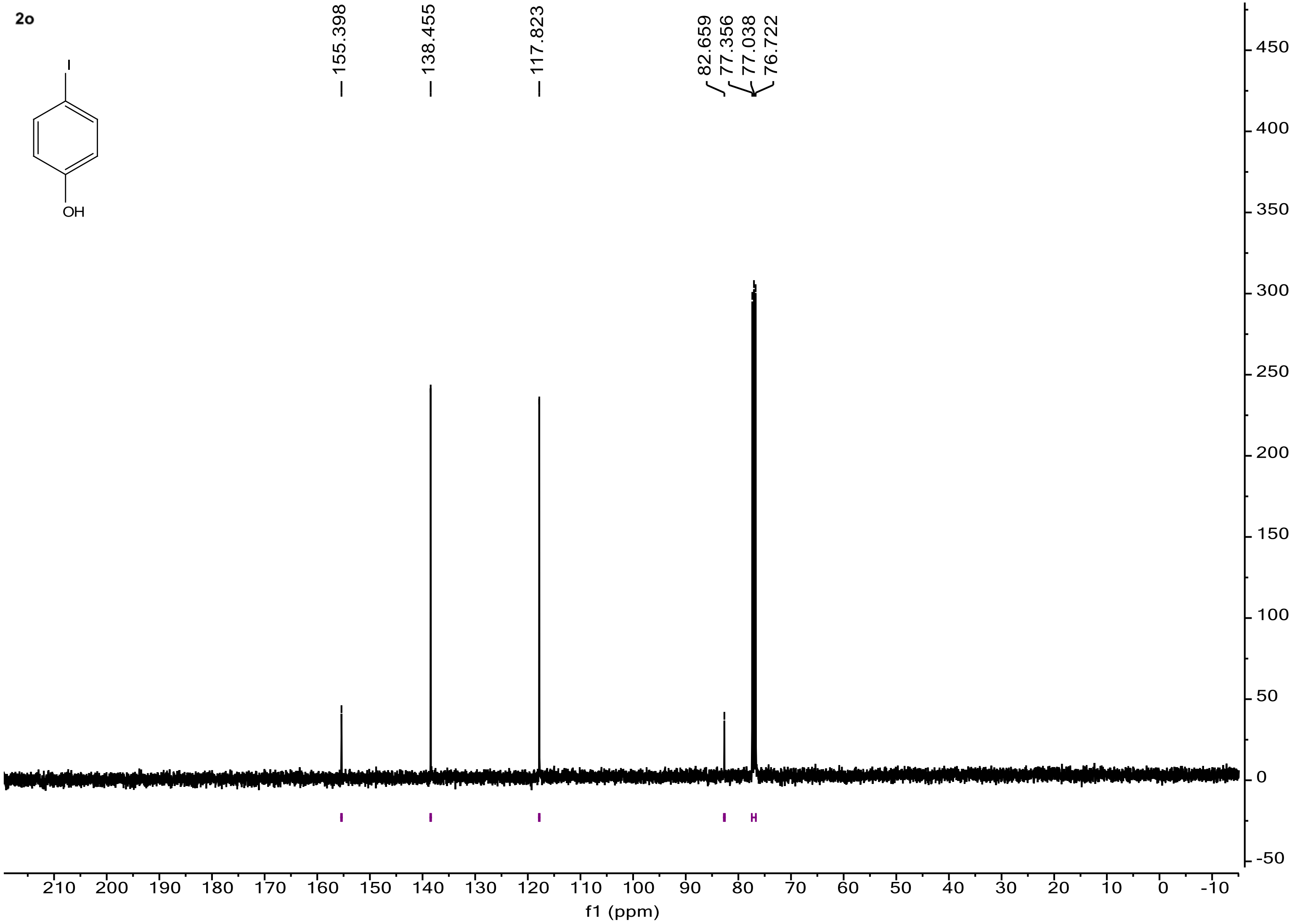

Supplementary Figure 57. ^{13}C NMR Spectrum for 2o

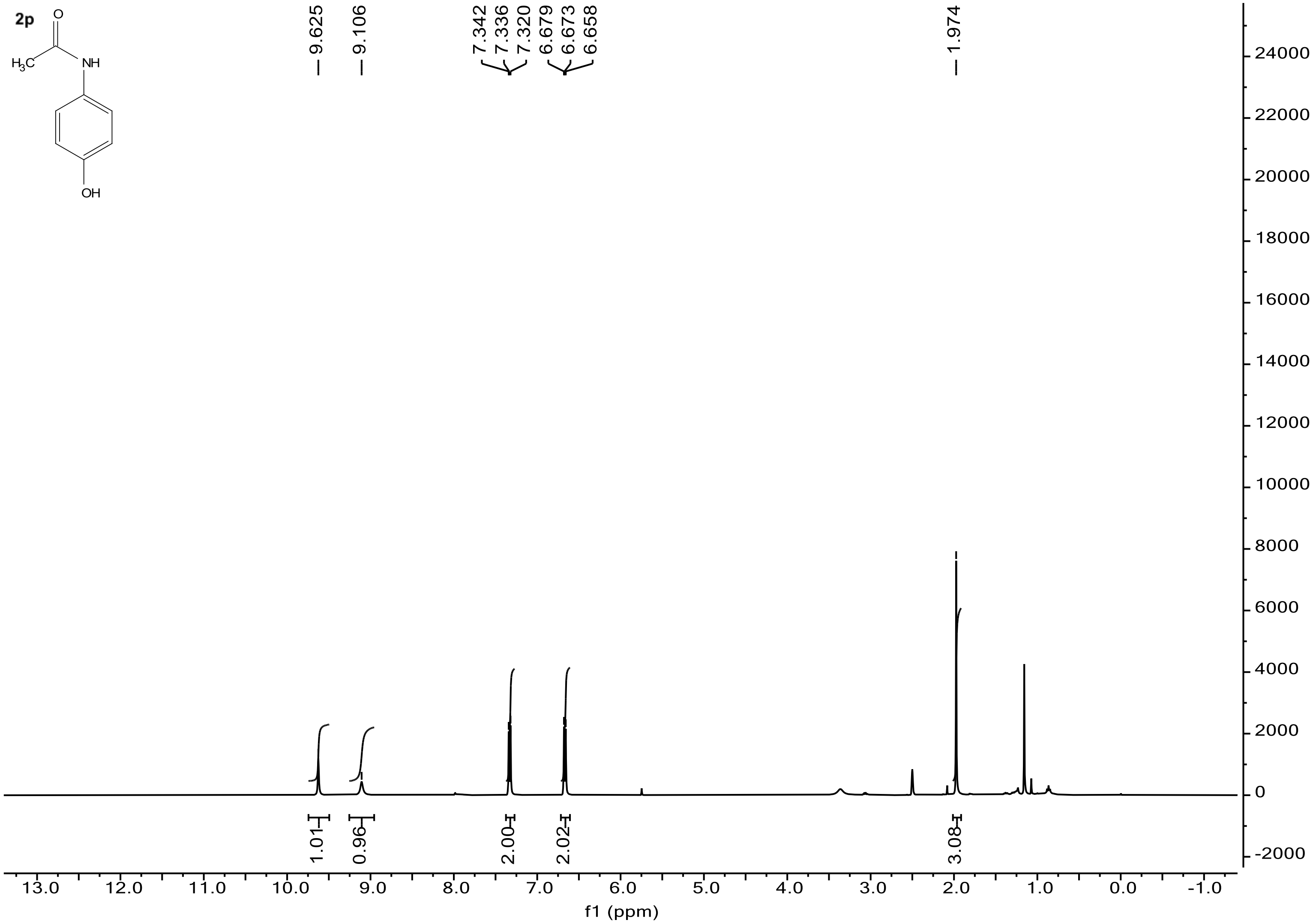

Supplementary Figure 58. ^{1}H NMR Spectrum for 2p

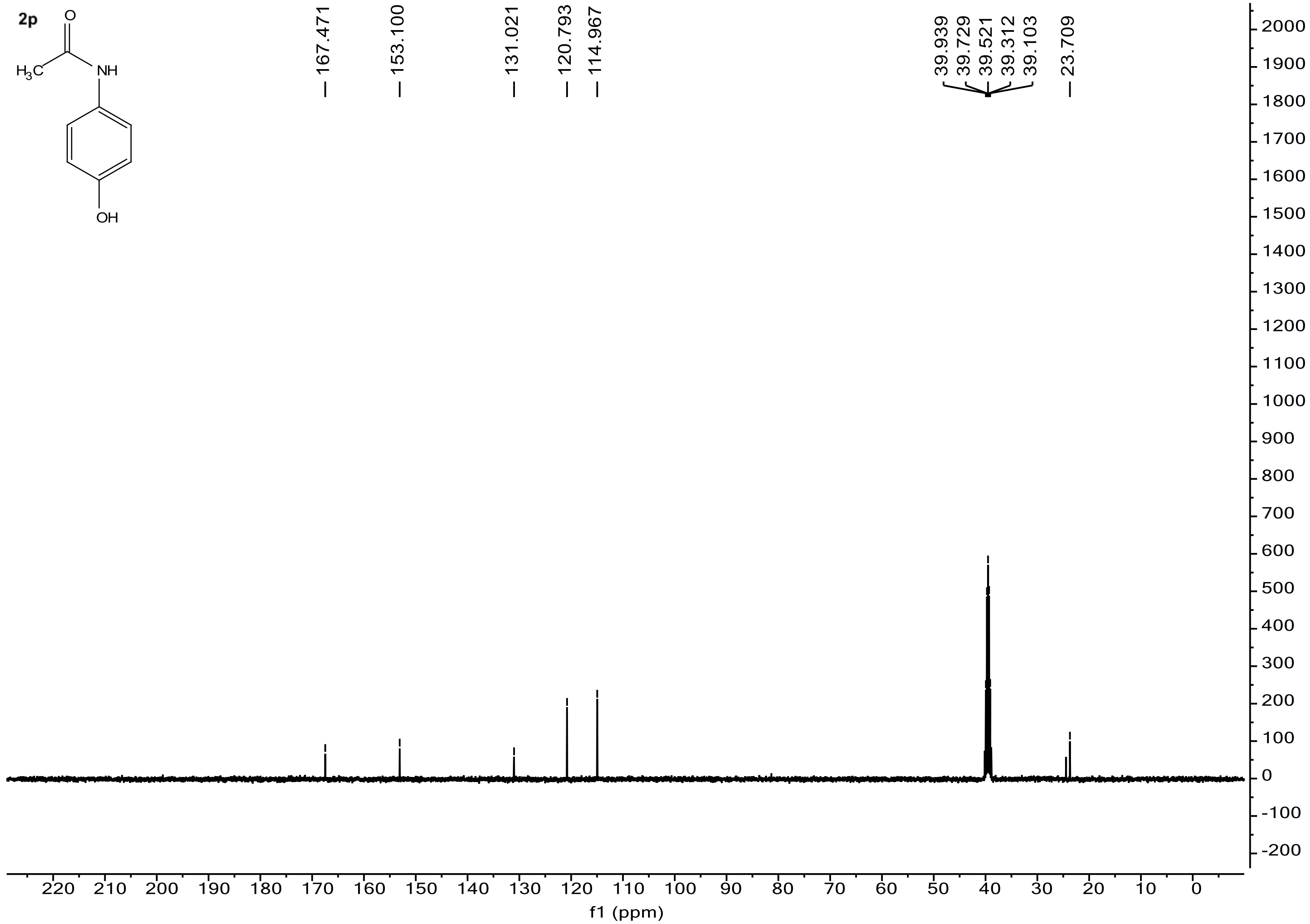

Supplementary Figure 59. ^{1}H NMR Spectrum for 2p

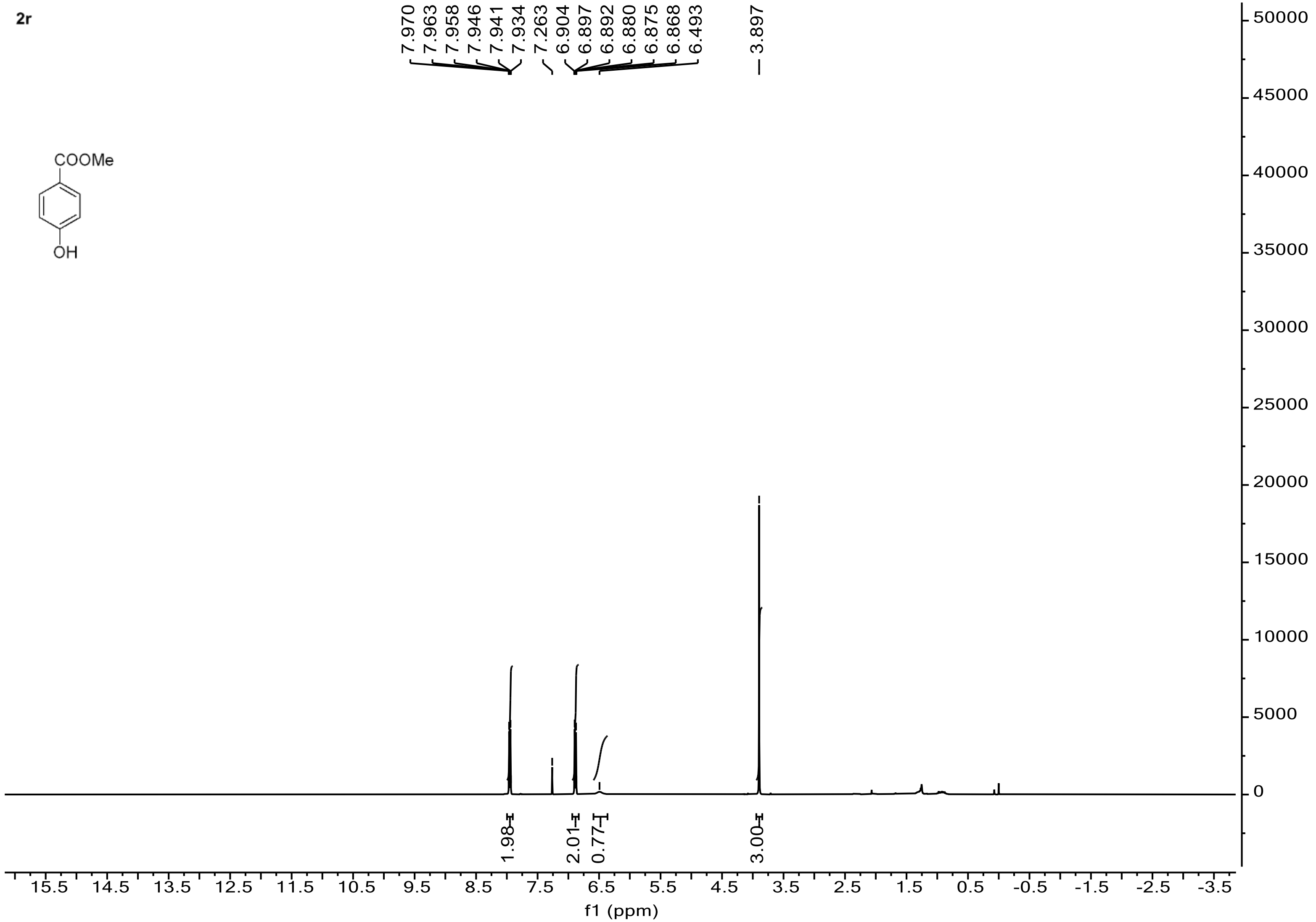

Supplementary Figure 60. ^{1}H NMR Spectrum for 2r

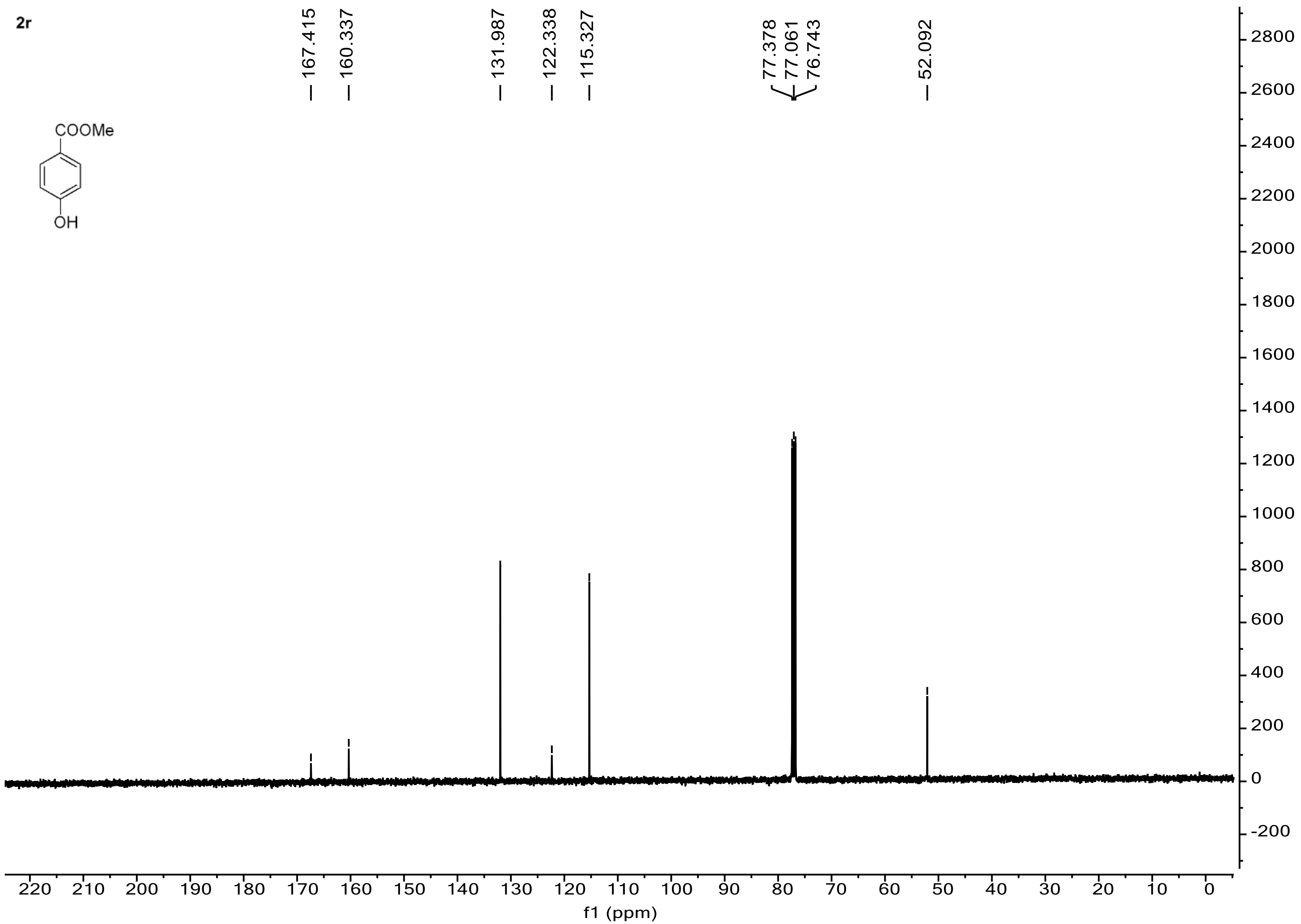

Supplementary Figure 61. ^{13}C NMR Spectrum for 2r

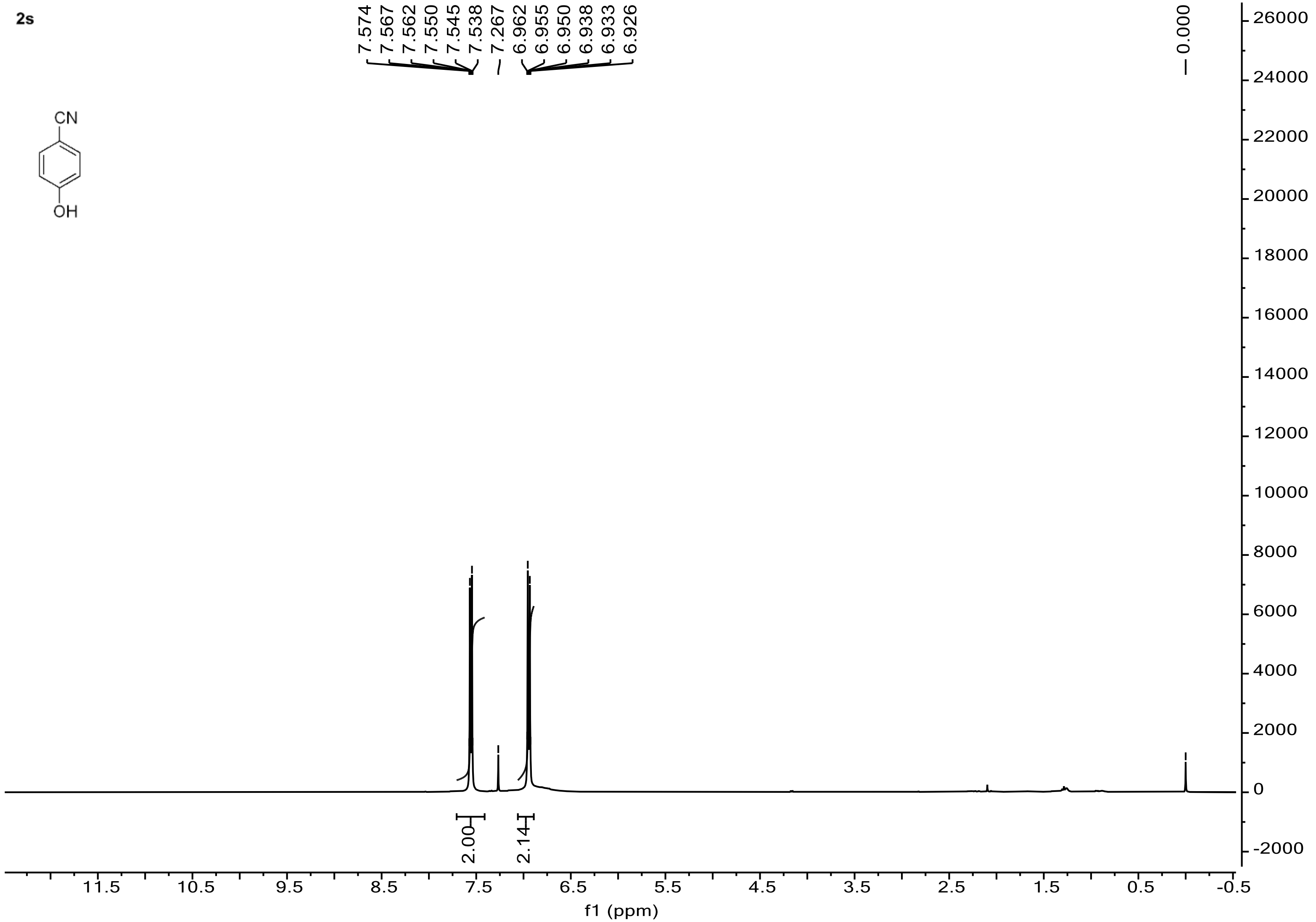

Supplementary Figure 62. ^{1}H NMR Spectrum for 2s

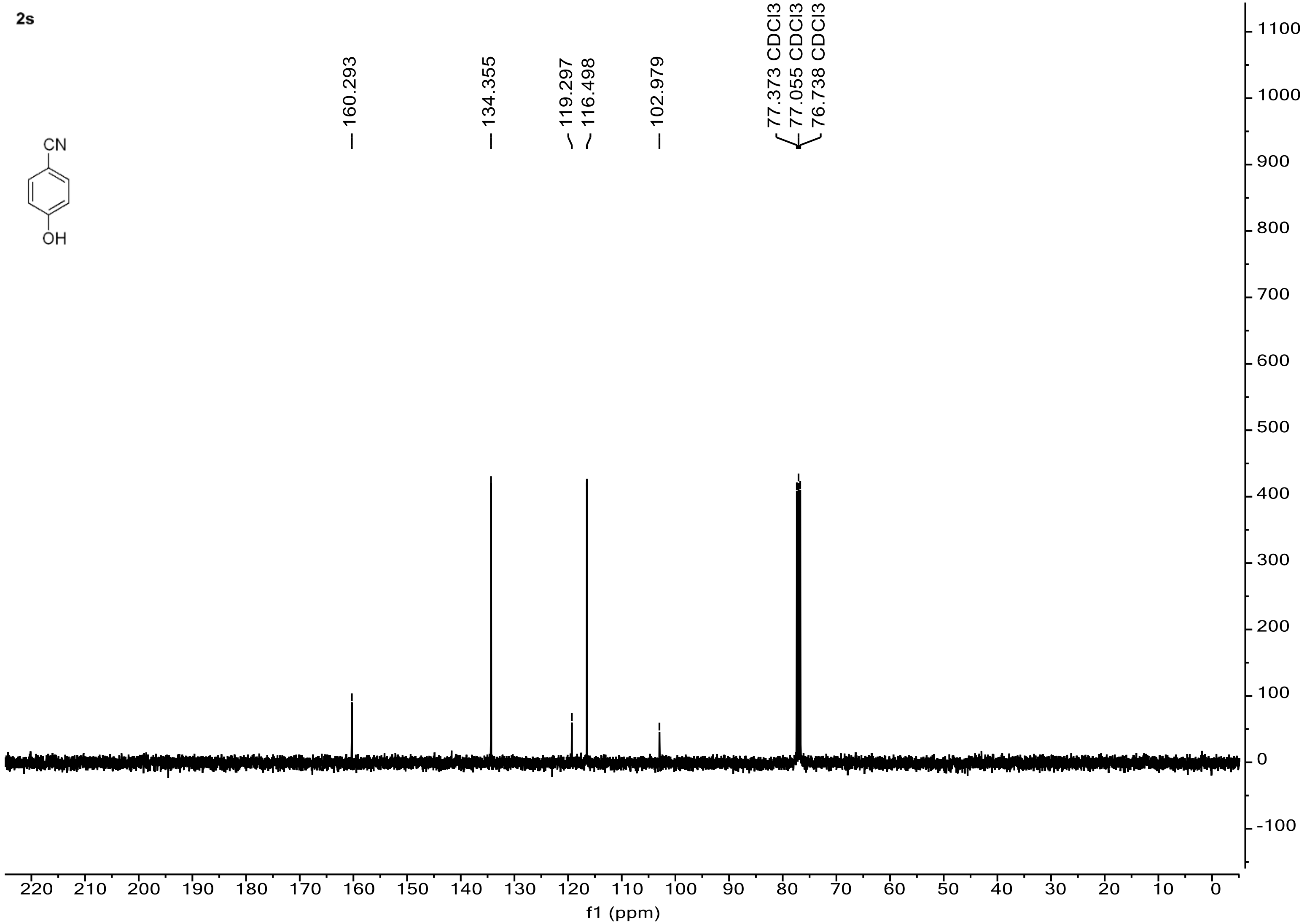

Supplementary Figure 63. ^{13}C NMR Spectrum for 2s

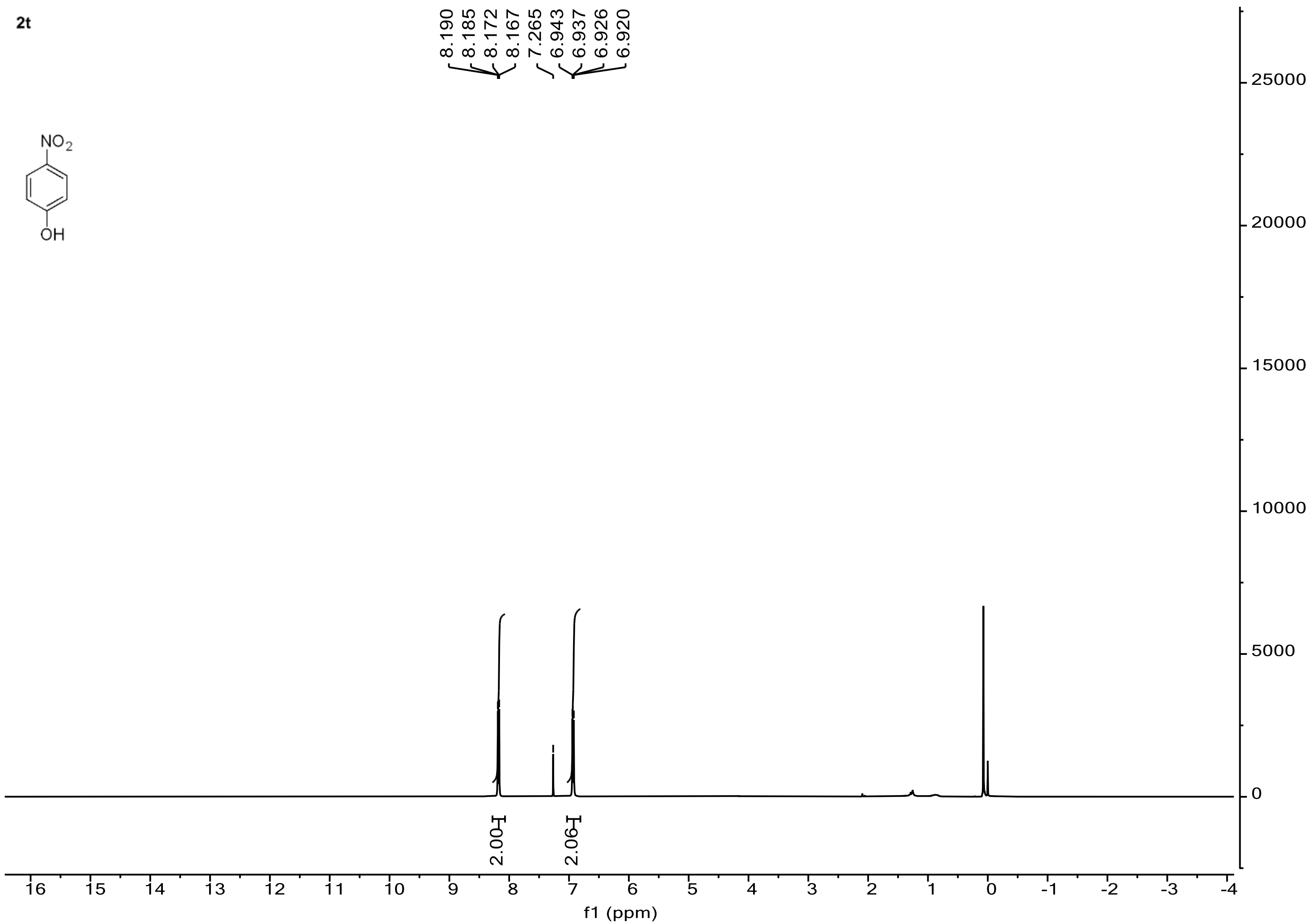

Supplementary Figure 64. ^{1}H NMR Spectrum for 2t

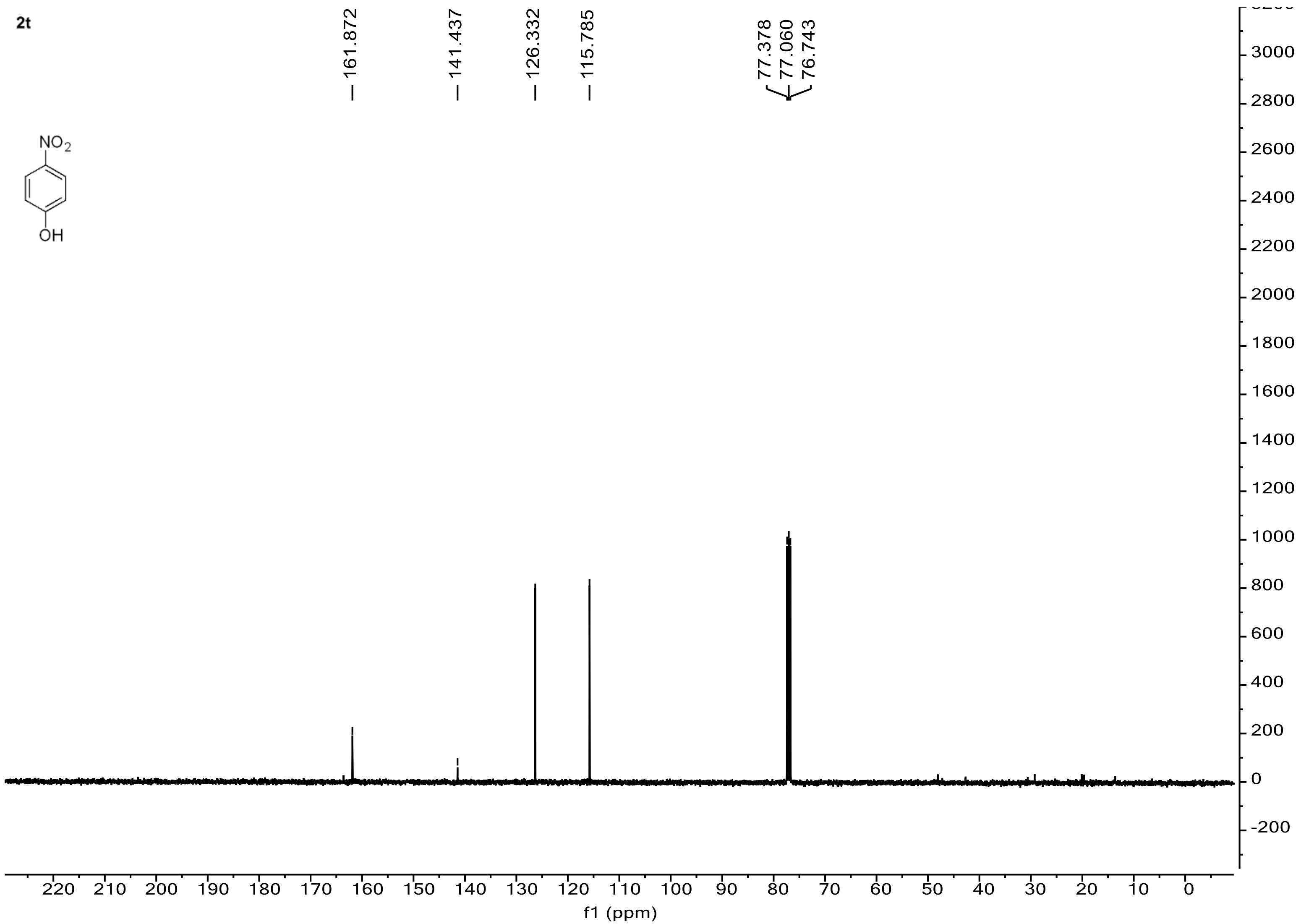

Supplementary Figure 65. ^{13}C NMR Spectrum for 2t

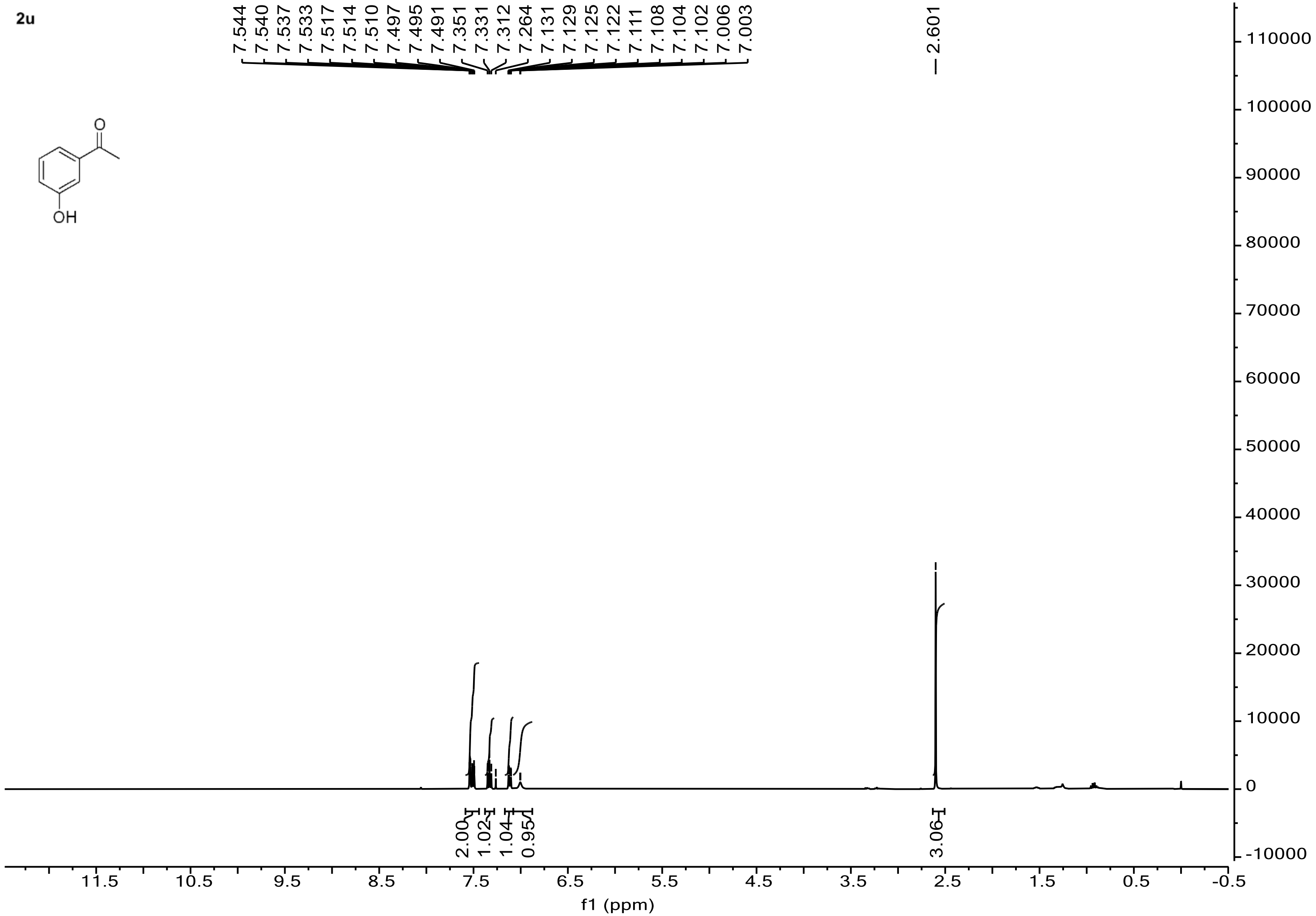

Supplementary Figure 66. ^{1}H NMR Spectrum for 2u

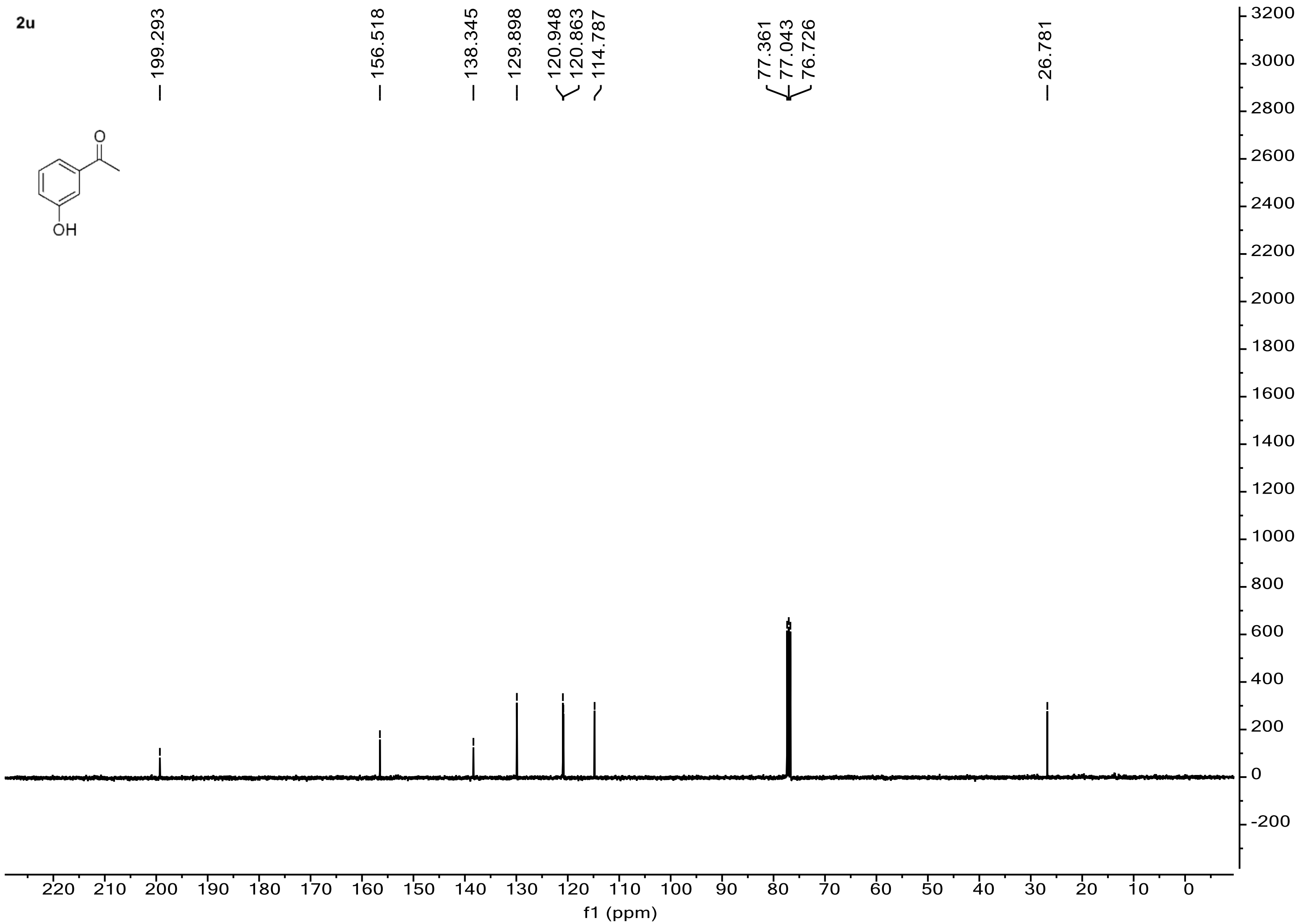

Supplementary Figure 67. ^{13}C NMR Spectrum for 2u

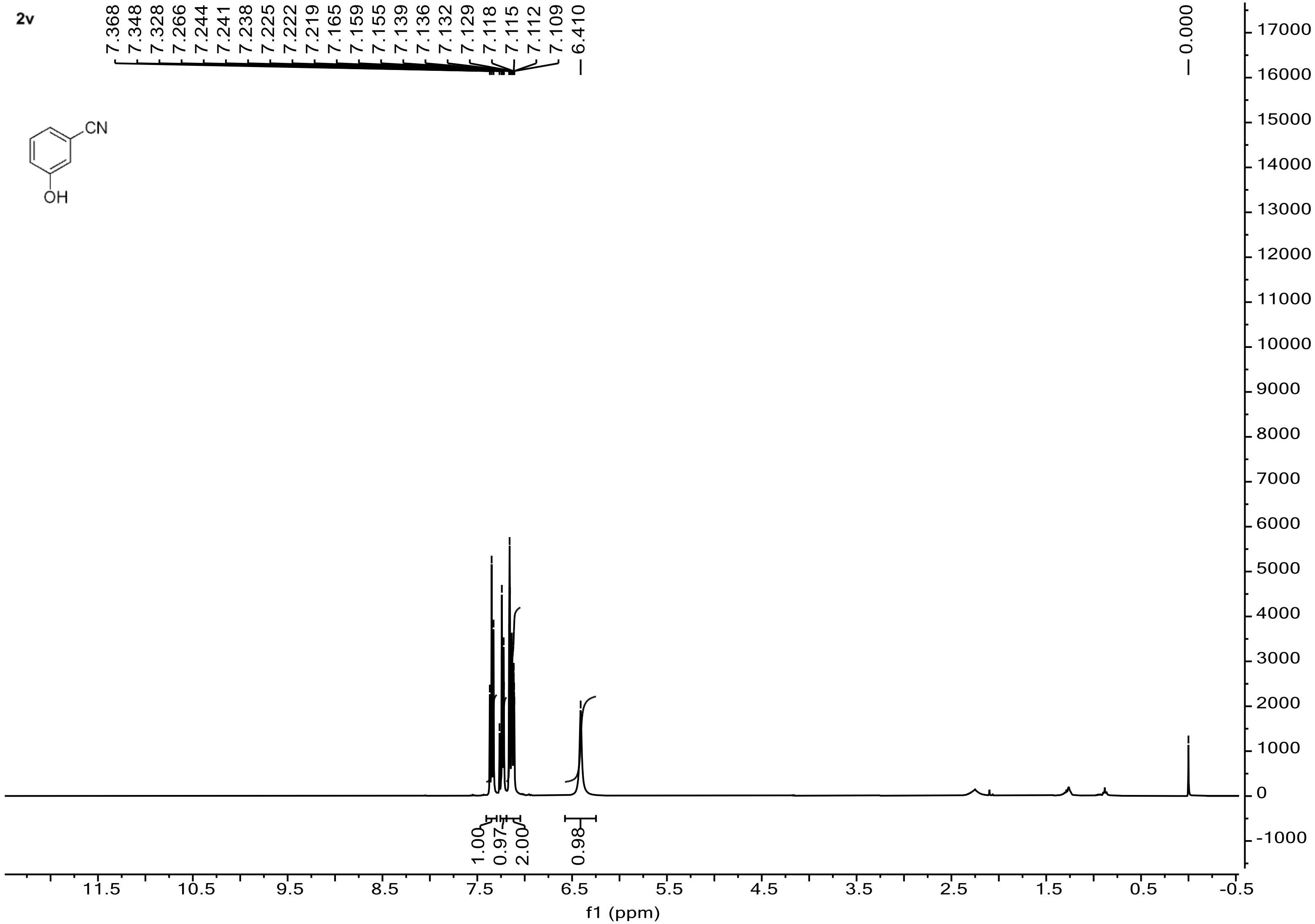

Supplementary Figure 68. ^{1}H NMR Spectrum for 2v

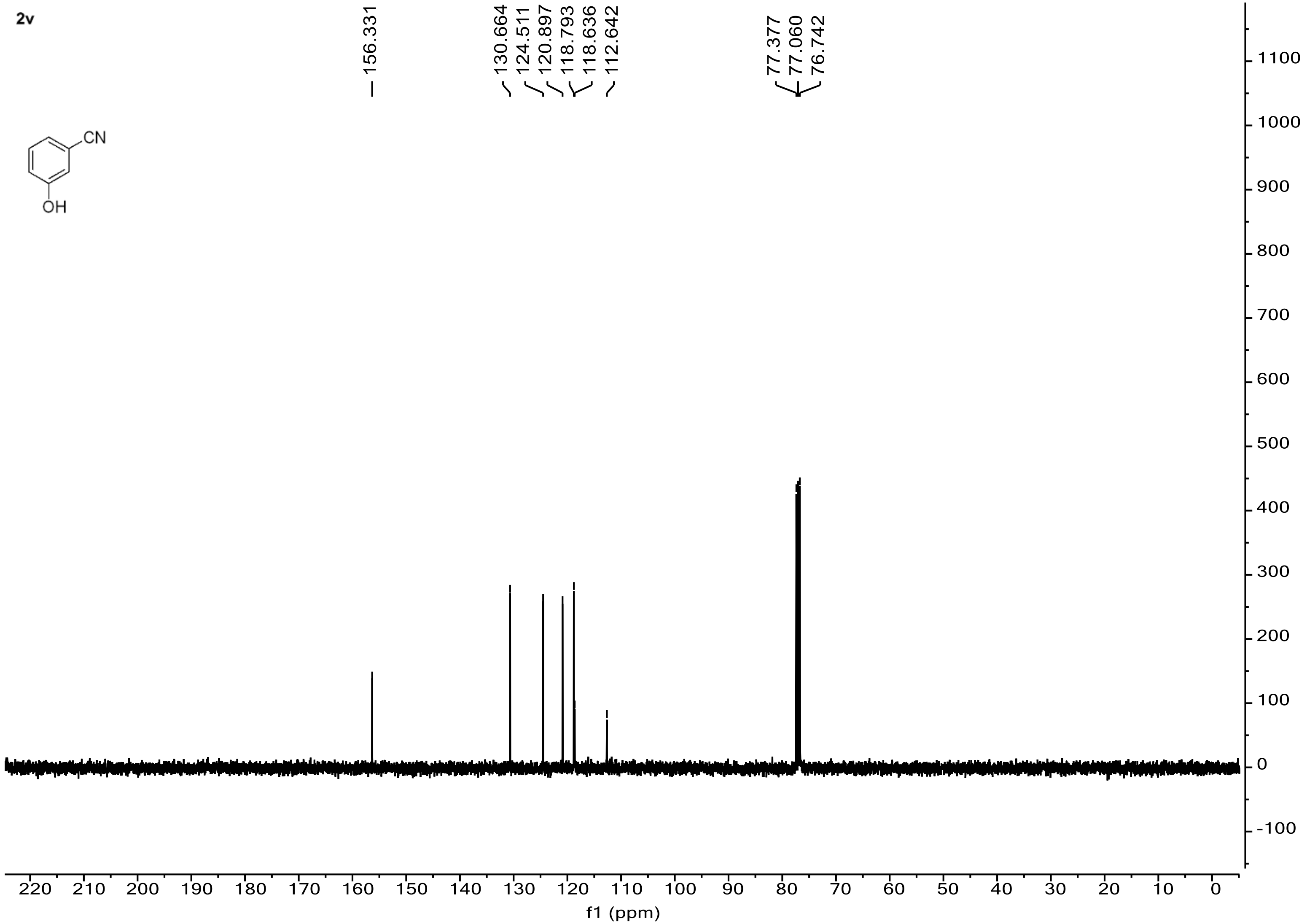

Supplementary Figure 69. ^{13}C NMR Spectrum for 2v

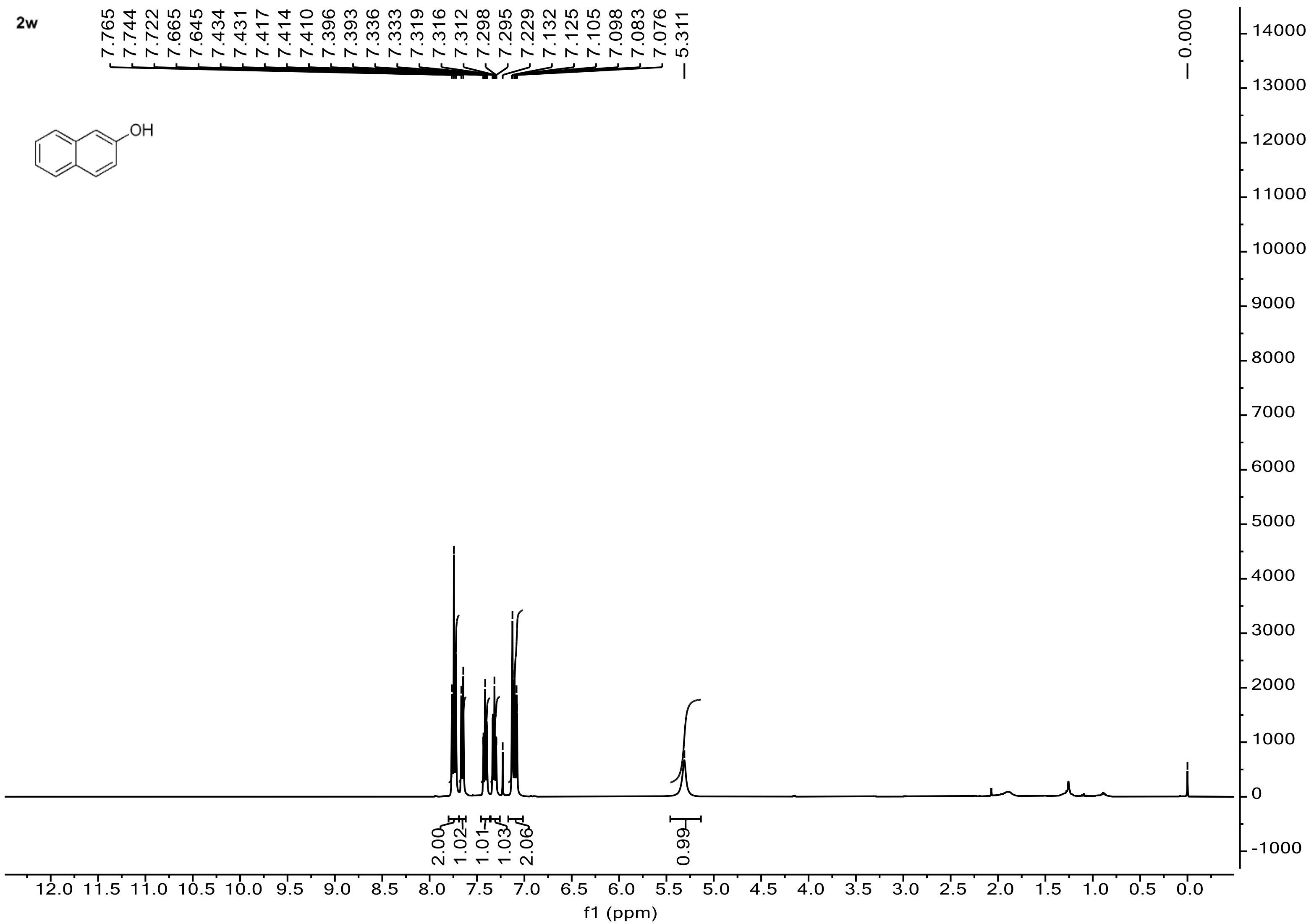

Supplementary Figure 70. ^{1}H NMR Spectrum for 2w

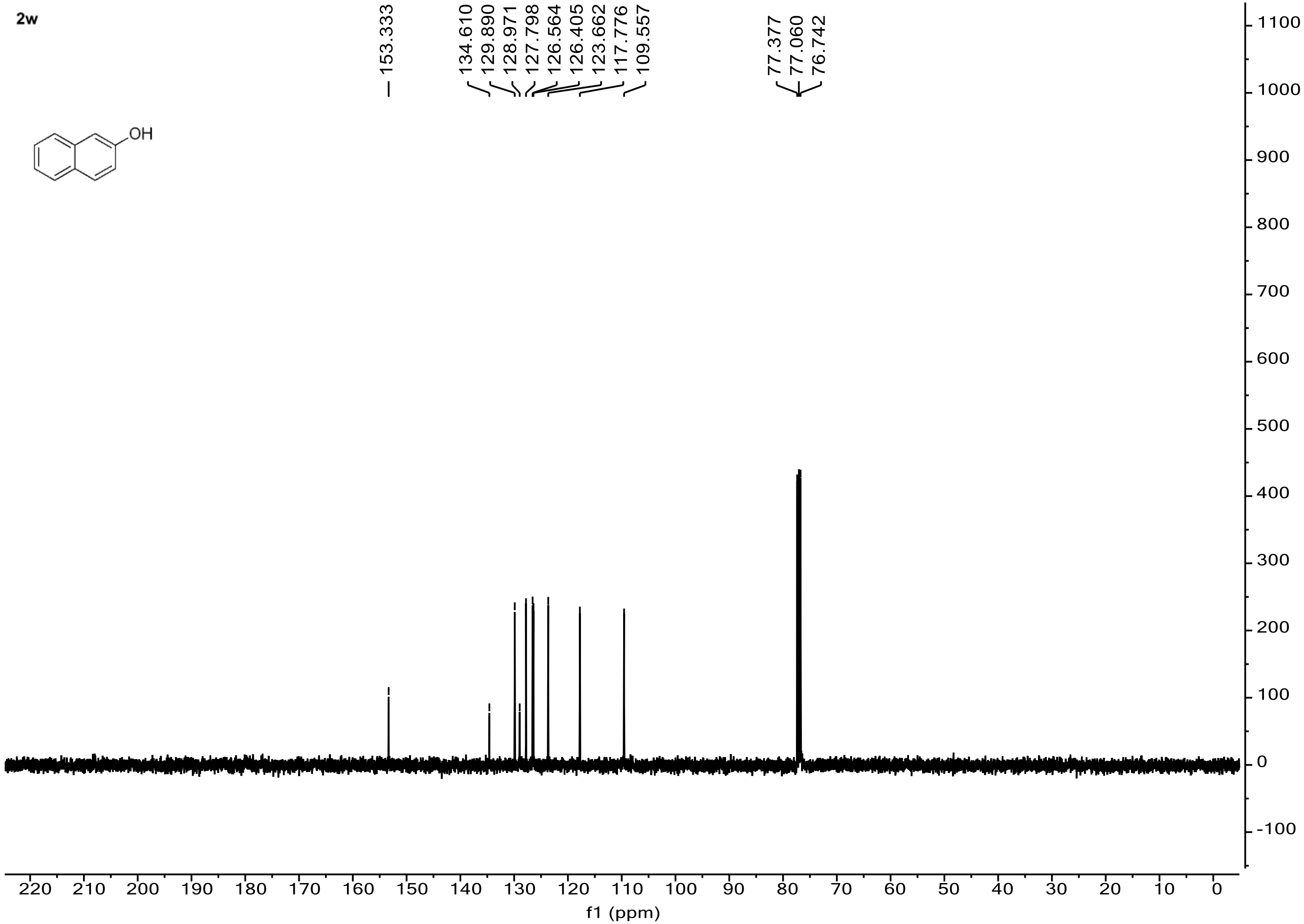

Supplementary Figure 71. ^{13}C NMR Spectrum for 2w

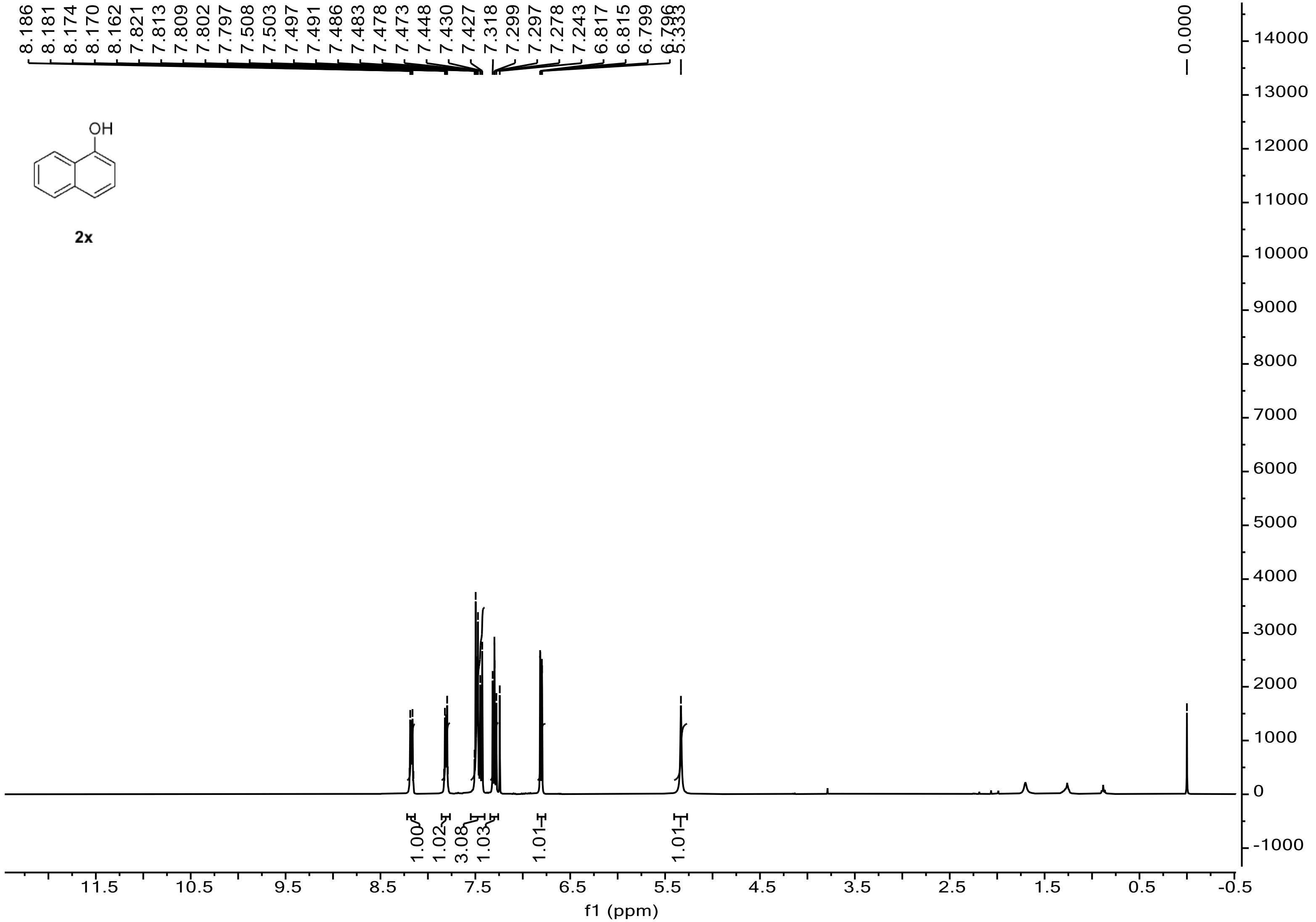

Supplementary Figure 72. ^{1}H NMR Spectrum for 2x

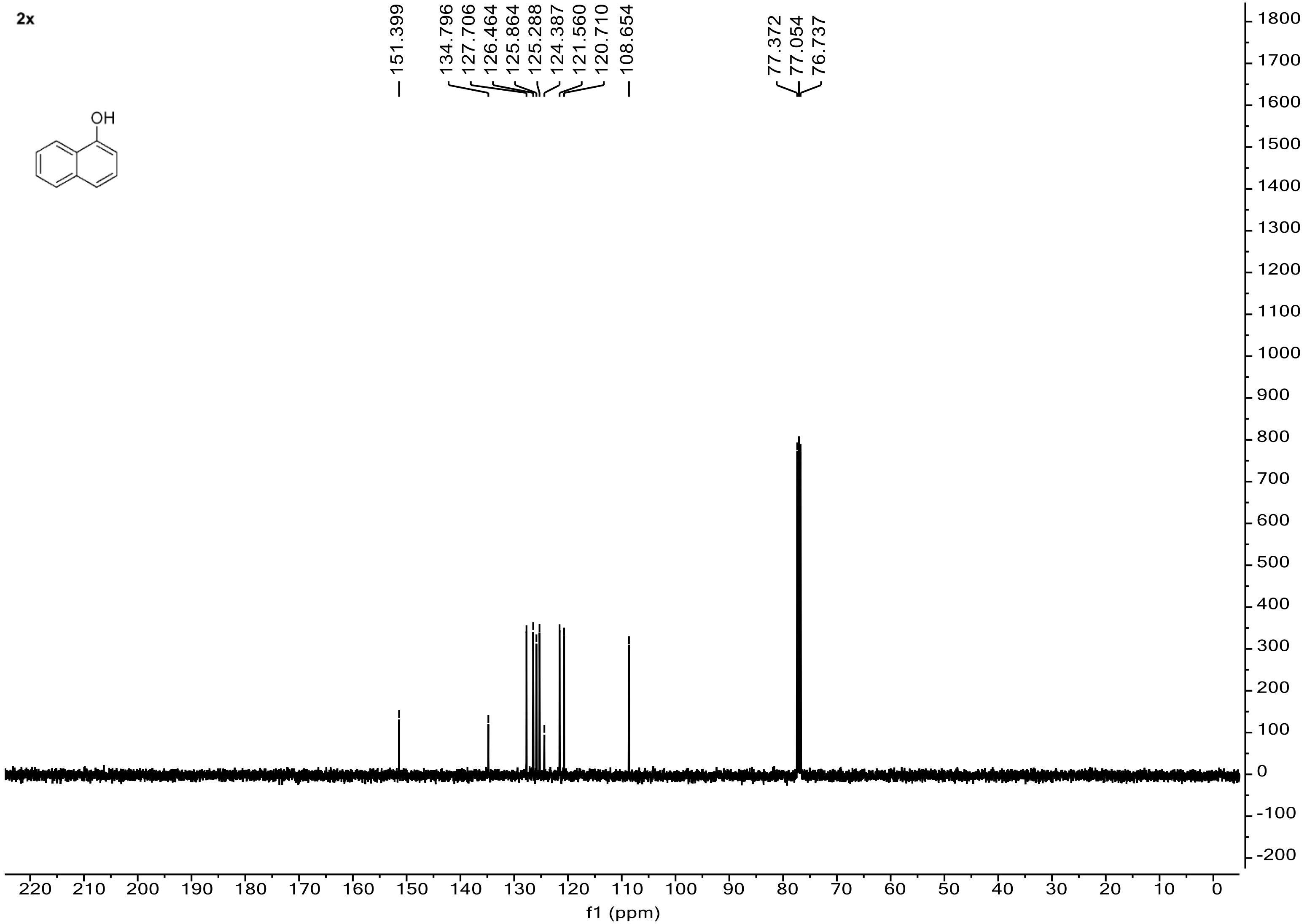

Supplementary Figure 73. ^{13}C NMR Spectrum for 2x

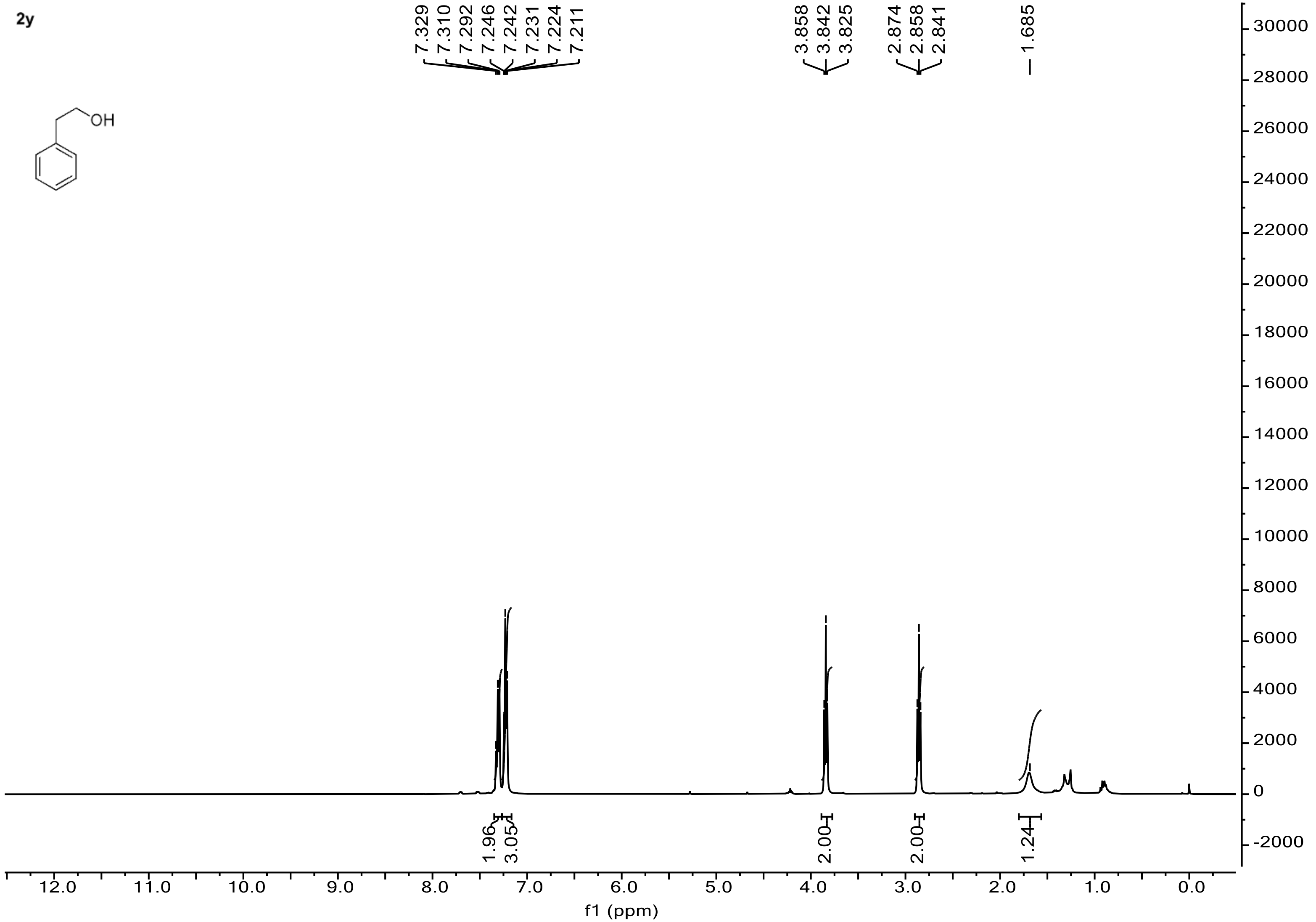

Supplementary Figure 74. ^{1}H NMR Spectrum for 2y

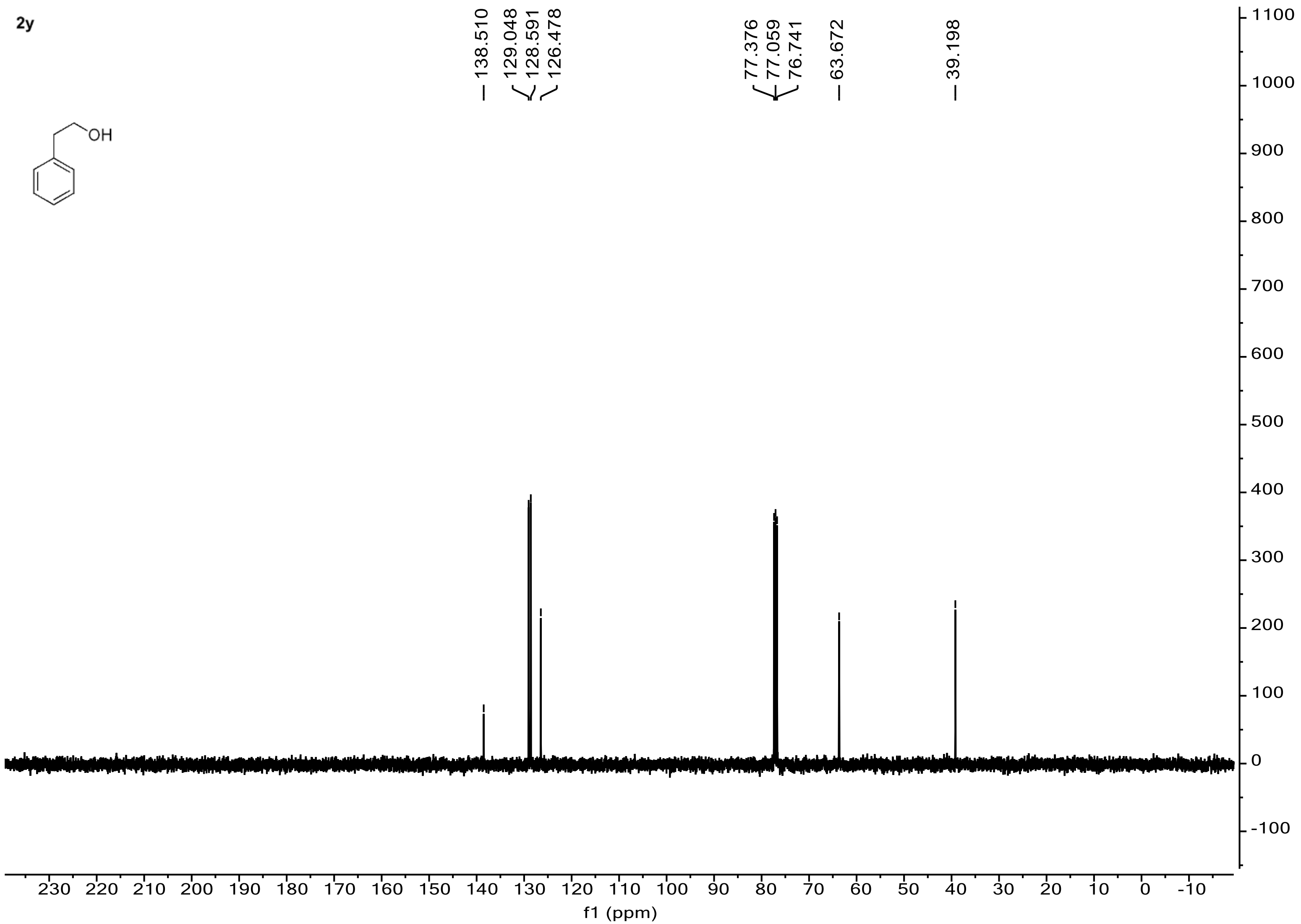

Supplementary Figure 75. ^{13}C NMR Spectrum for 2y

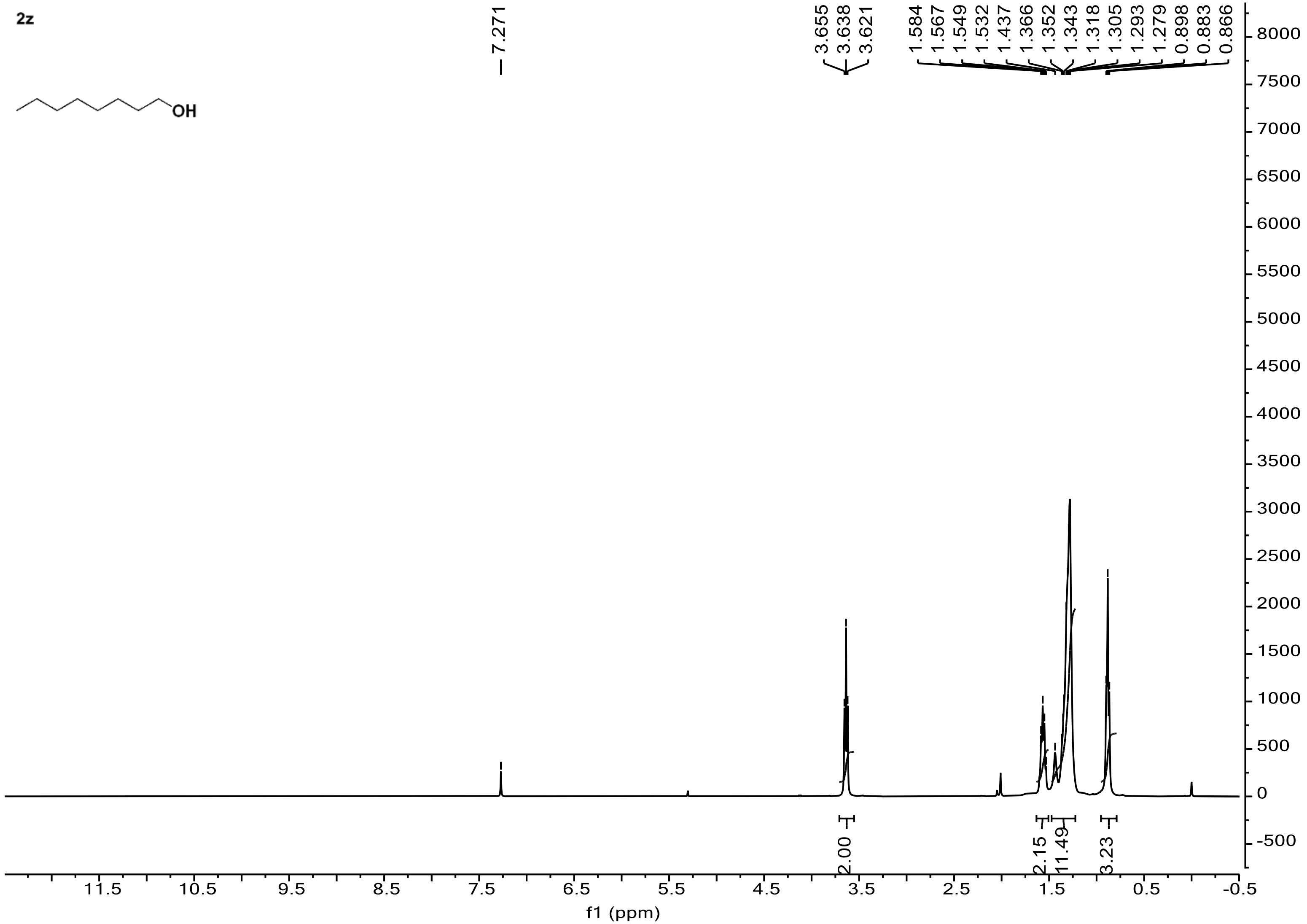

Supplementary Figure 76. ^{1}H NMR Spectrum for 2z

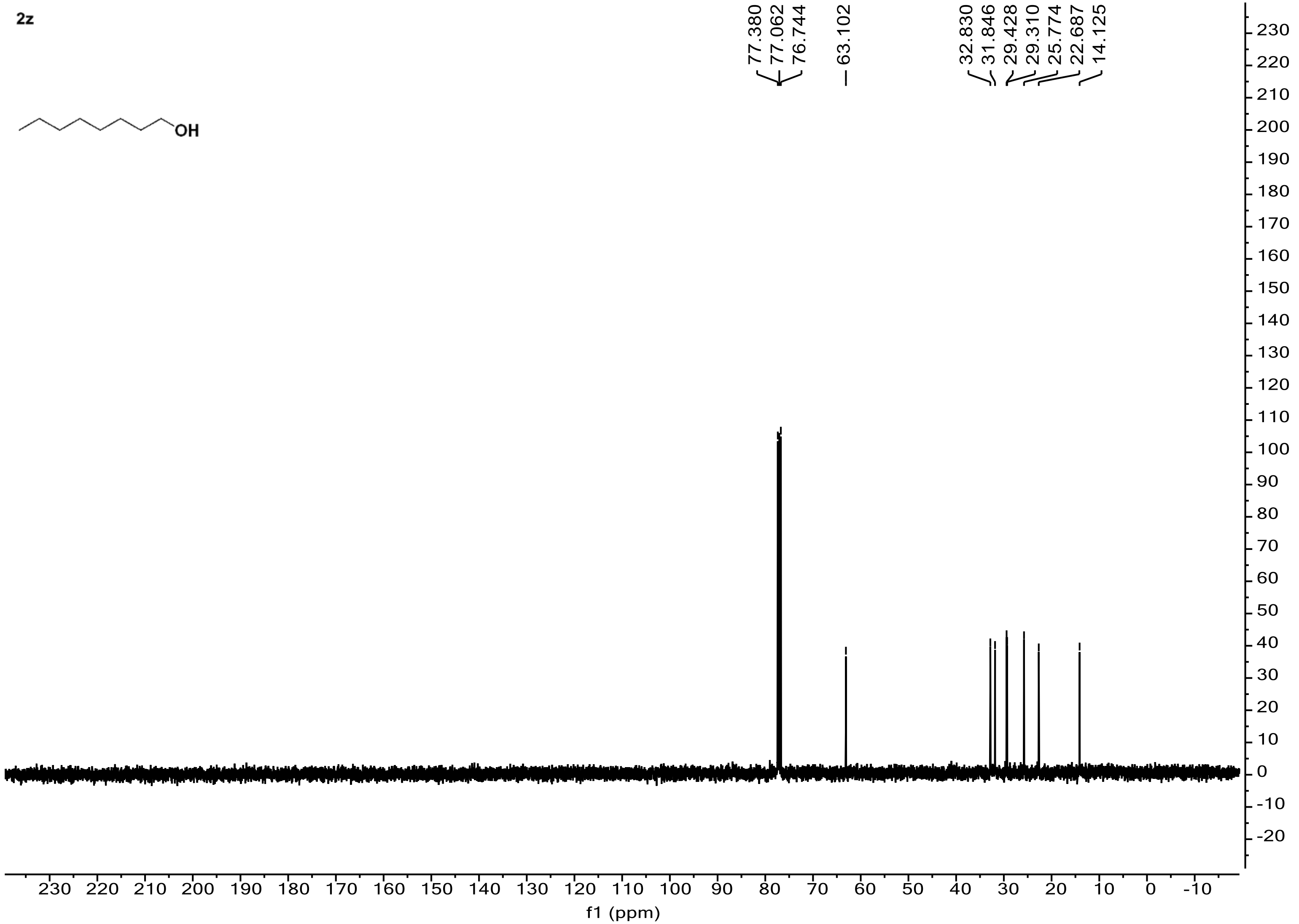

Supplementary Figure 77. ^{13}C NMR Spectrum for 2z

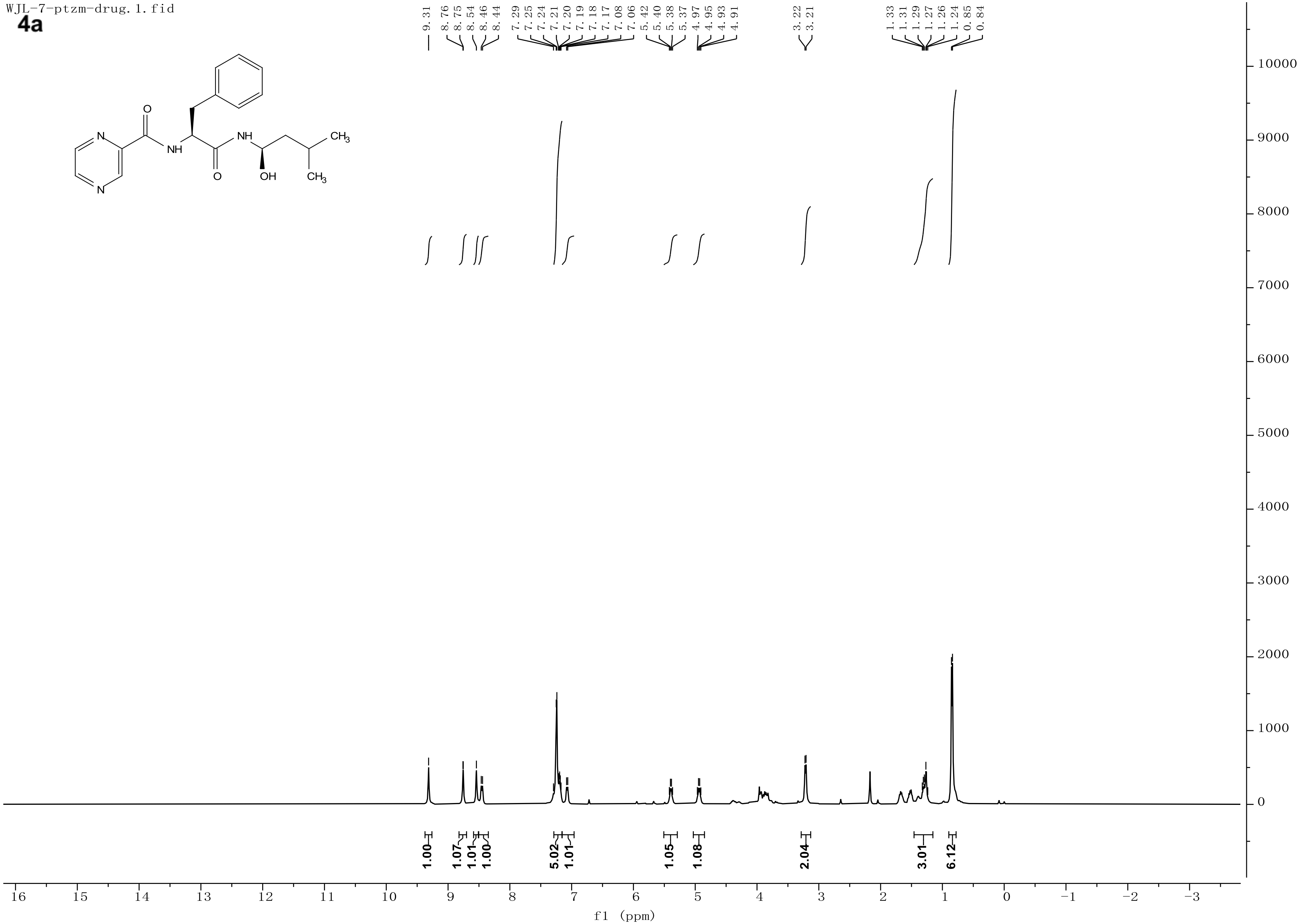

Supplementary Figure 78. ^{1}H NMR Spectrum for 4a

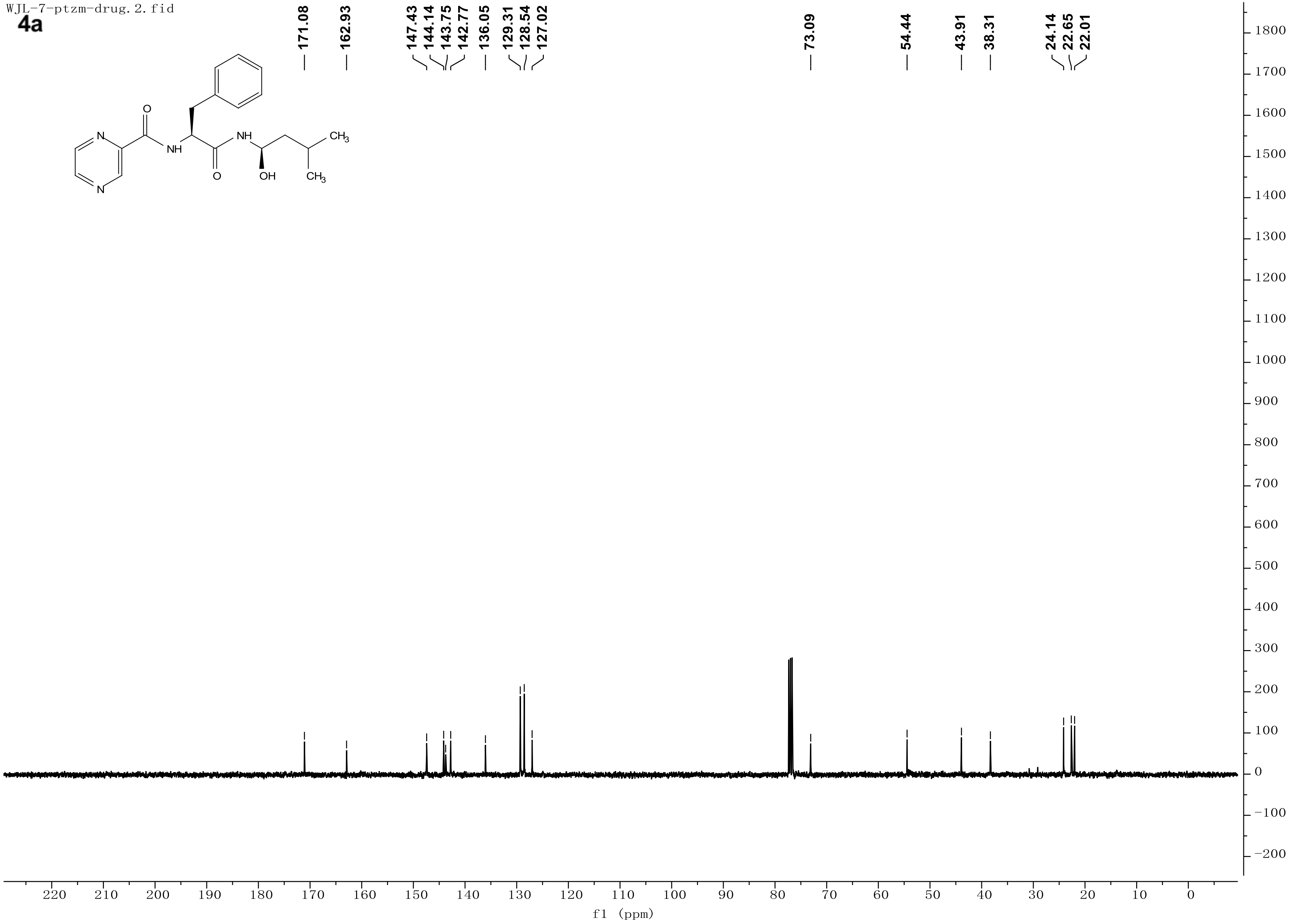

Supplementary Figure 79. ^{13}C NMR Spectrum for 4a

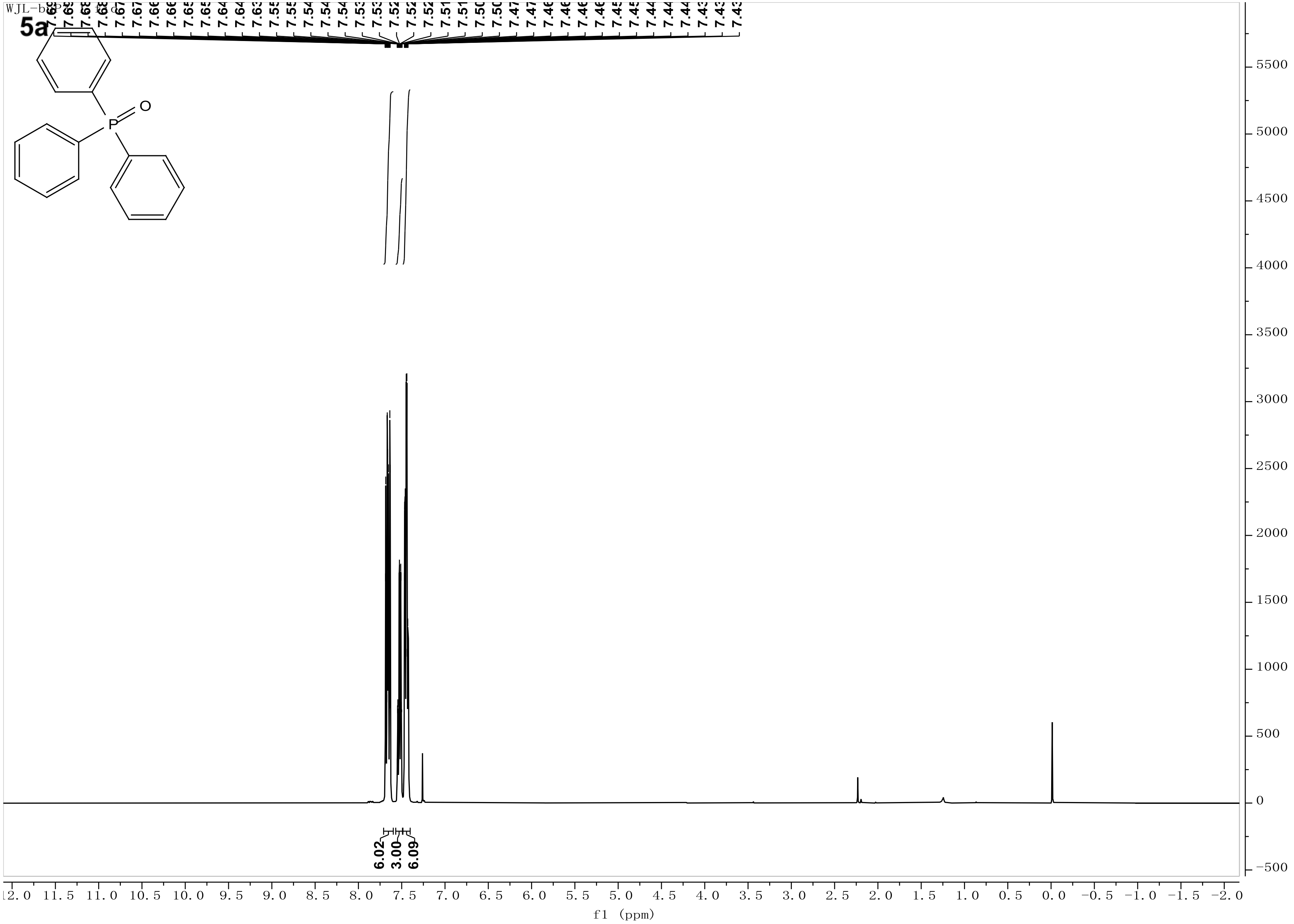

Supplementary Figure 80. ^{1}H NMR Spectrum for 5a

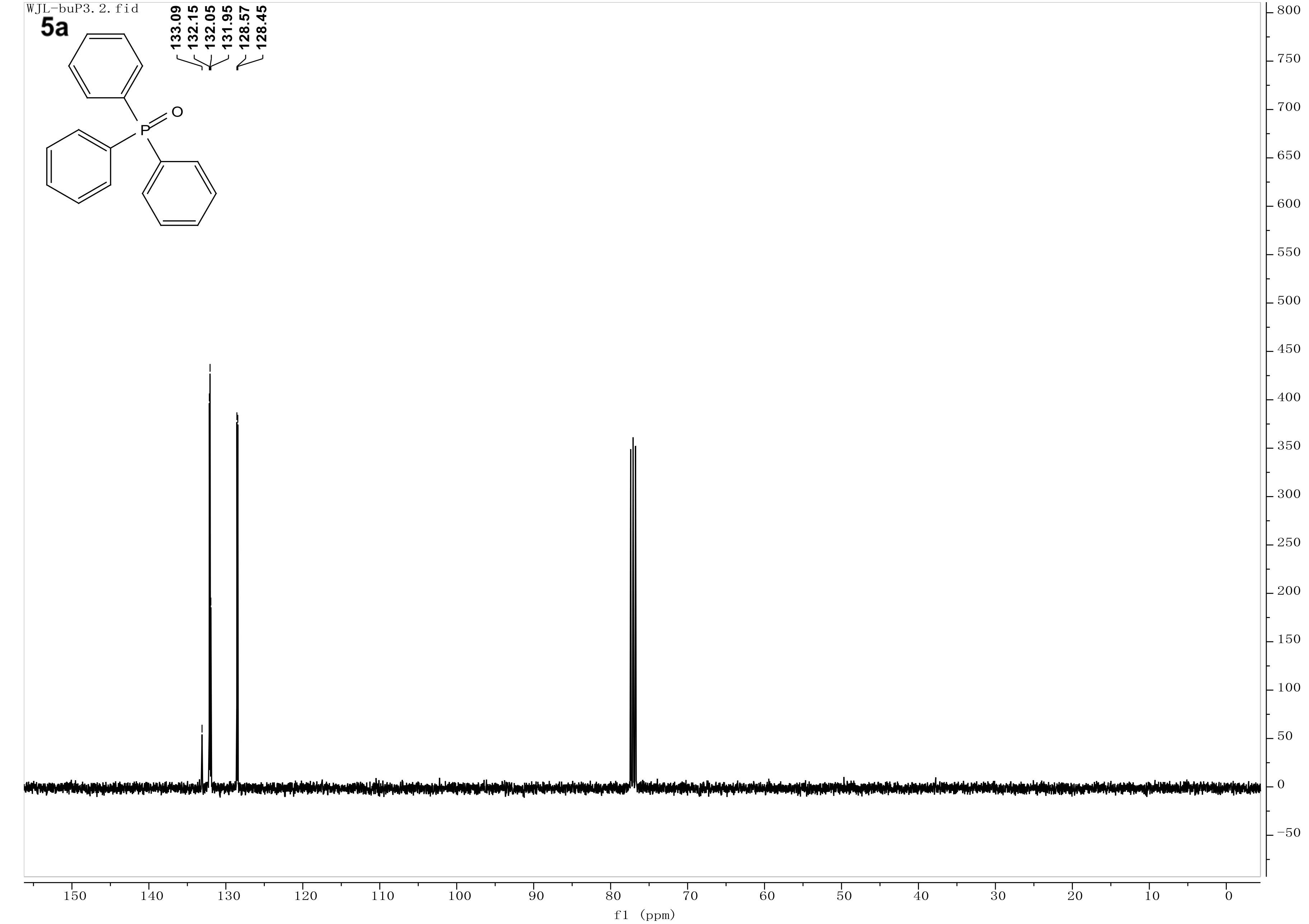

Supplementary Figure 81. ^{13}C NMR Spectrum for 5a

6a

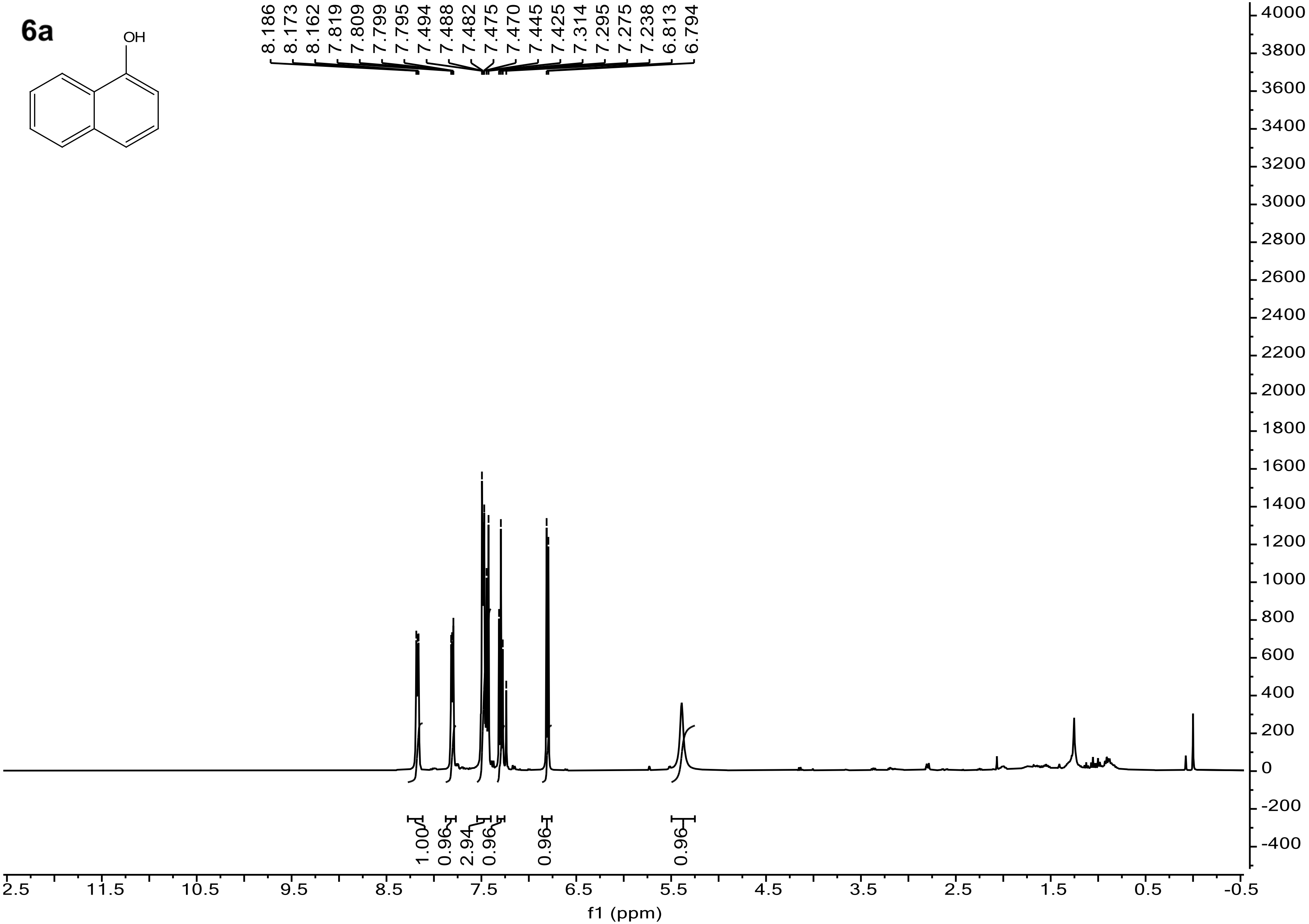

Supplementary Figure 82. ^{1}H NMR Spectrum for 6a

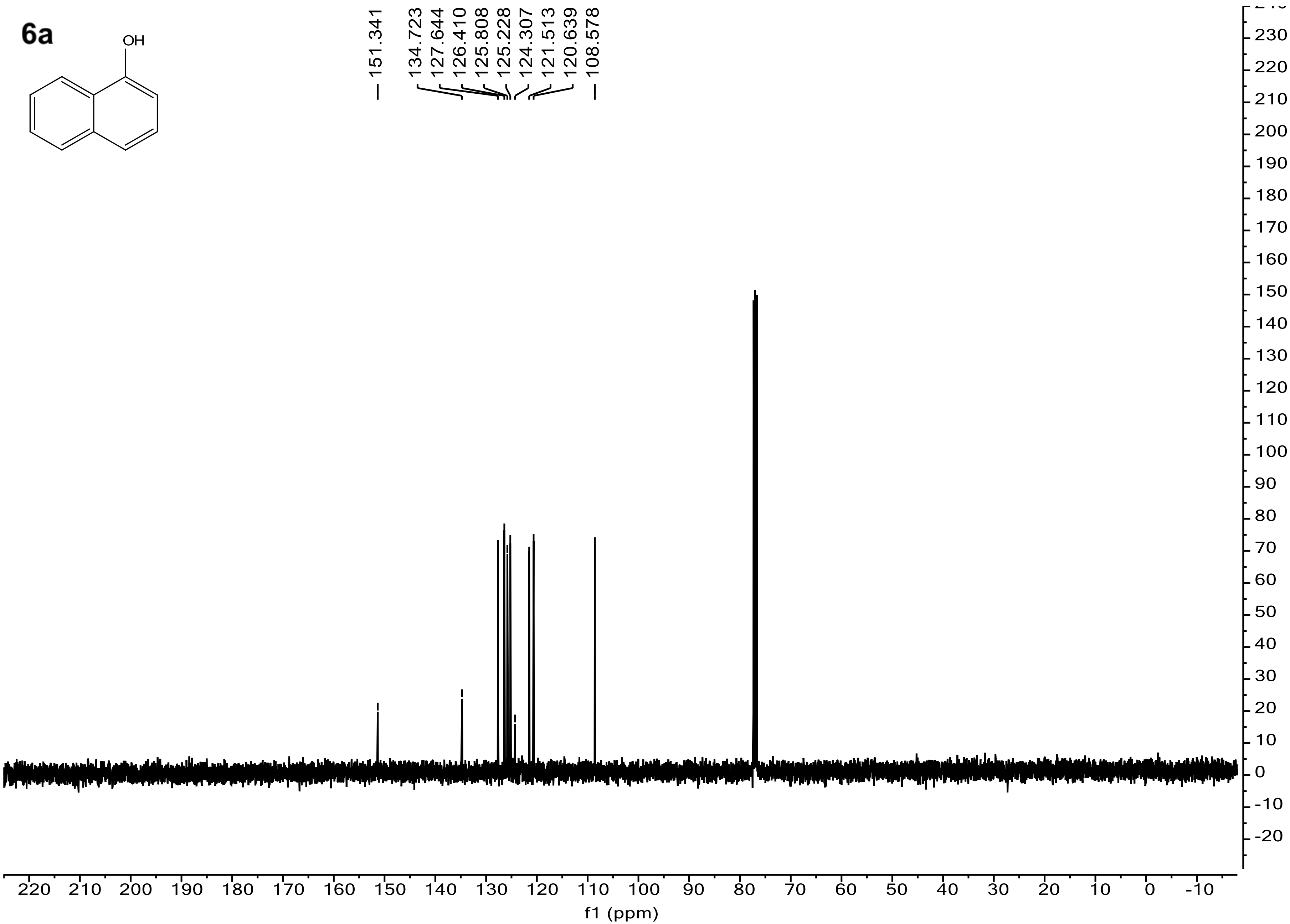

Supplementary Figure 83. ^{13}C NMR Spectrum for 6a

(G) Author Contributions

J.W., and N.J., conceived and designed the experiments; J.W. and J. M. carried out most of experiments; J.W., J.M., C.Z., Y. L., and N.J. analyzed data; J.W., Y. L. and N.J., wrote the paper; N.J. directed the project.

(H) References

1. Y.-Q. Zou, J.-R. Chen, X.-P. Liu, L.-Q. Lu, R. L. Davis, K. A. Jørgensen, W.-J. Xiao, Highly Efficient Aerobic Oxidative Hydroxylation of Arylboronic Acids: Photoredox Catalysis Using Visible Light. *Angew. Chem. Int. Ed.* **51**, 784-788 (2012).
2. T. Furuya, H. M. Kaiser, T. Ritter, Palladium-Mediated Fluorination of Arylboronic Acids. *Angew. Chem. Int. Ed.* **47**, 5993-5996 (2008).
3. S. Patnaik, A. D. Sadow, Interconverting Lanthanum Hydride and Borohydride Catalysts for C=O Reduction and C-O Bond Cleavage. *Angew. Chem. Int. Ed.* **58**, 2505-2509 (2019).
4. R. Wang, Y. Tang, M. Xu, C. Meng, F. Li, Transfer Hydrogenation of Aldehydes and Ketones with Isopropanol under Neutral Conditions Catalyzed by a Metal−Ligand Bifunctional Catalyst [Cp*Ir(2,2′-bpyO)(H_2O)]. *J. Org. Chem.* **83**, 2274-2281 (2018).
5. Y. Zhang, M. Dai, Z. Yuan, Methods for the detection of reactive oxygen species. *Anal. Methods*, **10**, 4625-4638 (2018).
6. S. K.Singh, S. M. Husain, A Redox-Based Superoxide Genera-tion System Using Quinone/Quinone Reductase. *ChemBioChem*, **19**, 1657-1663 (2018).
7. D. I. Bugaenkoa, A. V. Karchava, Electron Donor-Acceptor Complex Initiated Photochemical Phosphorus Arylation with Diaryliodonium Salts toward the Synthesis of Phosphine Oxides. *Adv. Synth. Catal.* **365**, 1893-1900 (2023).
8. J. M. Paolillo et. al. Anaerobic Hydroxylation of C(sp^3)–H Bonds Enabled by the Synergistic Nature of Photoexcited Nitroarenes. *J. Am. Chem. Soc.* **145**, 2794-2799 (2023).
9. Fulmer et al. NMR Chemical Shifts of Trace Impurities: Common Laboratory Solvents, Organics, and Gases in Deuterated Solvents Relevant to the Organometallic Chemist. *Organometallics*, **29**, 2176-2179 (2010).
10. M. H. Shaw, J. Twilton, David W. C. MacMillan, *Photoredox Catalysis in Organic Chemistry. J. Org. Chem.* **81**, 6898. (2016)
11. H. Hou, X. Zeng, X. Zhang, Production of Hydrogen Peroxide by Photocatalytic Processes. *Angew. Chem. Int. Ed.* **59**, 17356-17376 (2020).
12. N. A. Romero, D. A. Nicewicz, Organic Photoredox Catalysis. *Chem. Rev.* **116**, 10075-10166 (2016).
13. C. K. Prier, D. A. Rankic, D. W. C. MacMillan. Visible Light Photoredox Catalysis with Transition Metal Complexes: Applications in Organic Synthesis. *Chem. Rev.* **113**, 5322-5363 (2013).
14. T. Morofuji, G. Ikarashi, N. Kano, Photocatalytic C−H Amination of Aromatics Overcoming Redox Potential Limitations. *Org. Lett.* **22**, 2822-2827 (2020).
15. H. G. Roth, N. A. Romero, D. A. Nicewicz, Experimental and Calculated Electrochemical Potentials of Common Organic Molecules for Applications to Single-Electron Redox Chemistry. *Synlett*, **27**, 714-723 (2016).
16. P. Kohls, D. Jadhav, G. Pandey, O. Reiser, Visible Light Photoredox Catalysis: Generation and Addition of N-Aryltetrahydroisoquinoline-Derived R-Amino Radicals to Michael Acceptors. *Org. Lett.* **14**, 672-675 (2012).
17. S. Maity, M. Zhu, R. S. Shinabery, N. Zheng, Intermolecular [3+2] Cycloaddition of Cyclopropylamines with Olefins by Visible-Light Photocatalysis. *Angew. Chem. Int. Ed.* **51**, 222-226 (2012).
18. C. Wu, K. Jung, Y. Ma, W. Liu, C. Boyer, Unravelling an oxygen-mediated reductive quenching pathway for photopolymerisation under long wavelengths. *Nat. Commun.* **12**, 478 (2021).
19. L. Furst, J. M. R. Narayanam, C. R. J. Stephenson. Total Synthesis of (+)-Gliocladin C Enabled by Visible-Light Photoredox Catalysis. *Angew. Chem. Int. Ed.* **50**, 9655-9659 (2011).

20. N. Mandare et. al. Unravelling the photophysics of triphenylamine and diphenylamine dyes: a comprehensive investigation with ortho-, meta- and para-amido substituted derivatives. *New J. Chem.* **44**, 19061-19075 (2020).
21. J. M. R. Narayanam, J. W. Tucker, C. R. J. Stephenson. Electron-Transfer Photoredox Catalysis: Development of a Tin-Free Reductive Dehalogenation Reaction. *J. Am. Chem. Soc.* **131**, 8756-8757 (2009).
22. M. A. Cismesia, T. P. Yoon, Characterizing chain processes in visible light photoredox catalysis. *Chem. Sci.* **6**, 5426-5434 (2015).
23. S. Biswas, P. Chandu, S. Garai, D. Sureshkumar, Diastereoselective Hydroacylation of Cyclopropenes by Visible-Light Photocatalysis. *Org. Lett.* **25**, 7863-7867 (2023).
24. J. Hu, J. Wang, T. H. Nguyen, N. Zheng, The chemistry of amine radical cations produced by visible light photoredox catalysis. *Beilstein J. Org. Chem.* **9**, 1977 (2013).
25. Y. Yamamoto, S. Kodama, A. Nomoto, A. Ogawa, Innovative green oxidation of amines to imines under atmospheric oxygen. *Org. Biomol. Chem.* **20**, 9503-9521 (2022).
